# Hydrogels in the Immune Context: In Vivo Applications for Modulating Immune Responses in Cancer Therapy

**DOI:** 10.3390/gels11110889

**Published:** 2025-11-04

**Authors:** Mara R. Lanis, Sujin Kim, Jonathan P. Schneck

**Affiliations:** 1 Department of Biomedical Engineering, Johns Hopkins University School of Medicine, Baltimore, MD 21218, USA; mlanis1@jh.edu (M.R.L.); skim544@jh.edu (S.K.); 2 Johns Hopkins Translational ImmunoEngineering Center, Johns Hopkins University School of Medicine, Baltimore, MD 21205, USA; 3 Institute for Cell Engineering, Johns Hopkins University School of Medicine, Baltimore, MD 21205, USA; 4 Department of Pathology, Johns Hopkins University School of Medicine, Baltimore, MD 21205, USA; 5 Department of Medicine, Johns Hopkins University School of Medicine, Baltimore, MD 21205, USA; 6 Department of Oncology, Johns Hopkins University School of Medicine, Baltimore, MD 21205, USA; 7 Institute for NanoBioTechnology, Johns Hopkins University, Baltimore, MD 21218, USA

**Keywords:** hydrogels, cancer, immunotherapy, tumor microenvironment, drug release, T cells, dendritic cells, macrophages, in vivo

## Abstract

In response to growing clinical demands for more targeted and effective immunotherapies to treat cancer, biomaterial-based strategies have emerged as powerful tools for locally regulating immune responses. Among these, hydrogels, a class of biocompatible and tunable polymeric networks, are increasingly being leveraged for their high versatility and adaptability for creating tailored immune environments. By enabling controlled delivery of immune cues and direct cellular engineering, hydrogels utilized in vivo can precisely regulate both innate and adaptive immune responses while minimizing systemic toxicity. In this review, we outline essential hydrogel design features necessary for in vivo functionality including injectability, degradation kinetics, and immune-specific functionalization. Building on these principles, we explore how hydrogels have been employed to enhance T cell activation and dendritic cell maturation and guide macrophage reprogramming. Beyond cellular modulation, we further examine the use of hydrogels for cytokine and immunoregulatory agent delivery, tumor microenvironment remodeling, and the creation of tertiary-like lymphoid structures. Finally, we review recently completed and ongoing clinical trials of hydrogels in the cancer immunotherapy space. Together, these insights underscore the growing potential of in vivo hydrogel systems as immuno-interactive platforms capable of reshaping immune responses across diverse disease contexts.

## 1. Introduction

The immune system plays a central role in maintaining homeostasis and responding to a wide array of physiological disruptions, including infection, cancer, and tissue injury. Its capacity to distinguish self from non-self, initiate and resolve inflammation, and coordinate complex multicellular interactions makes it a powerful, yet challenging, therapeutic target [1]. In recent decades, advances in the field of immunotherapy have transformed the treatment landscape for many diseases, particularly in oncology. Broadly, cancer immunotherapies act by reawakening the immune system’s ability to recognize and eliminate malignant cells. Some of the most widely studied immunotherapies include immune checkpoint inhibitors (ICIs) such as anti–PD-1 (aPD-1) and anti–CTLA-4 antibodies, which relieve inhibitory signaling pathways that restrain T cell activation and adoptive cell therapies (ACT), which supply patients with ex vivo–expanded, tumor-reactive lymphocytes to enhance cytotoxic responses [2,3]. Other strategies, such as cancer vaccines and monoclonal antibody therapies, have aimed to influence the immune response by improving antigen presentation or by directly targeting tumor cells for immune cell-mediated cytotoxicity, respectively [4]. ICIs and aACT in particular have shown great promise, yielding durable responses in select patient populations [2,3,5].

Unfortunately, despite these successes, many patients have been unable to derive lasting benefit from these treatments due to their inadequate control of the immune response. Current immunotherapy treatments can often give rise to systemic toxicities, have short-lived efficacy, and show suboptimal performance in immunosuppressive tumor microenvironments (TMEs) [4,6,7,8,9,10,11]. These shortcomings reflect a broader need for approaches that can modulate immune activity with greater control and specificity to promote improved patient responses. Given the immune system’s dynamic and context-dependent nature, strategies are required that can localize treatment to sites of disease and promote physiologically relevant responses in vivo.

In response to these challenges, researchers have recently turned their attention to biomaterials that can interface with the immune system in more controlled and programmable ways than traditional systemic therapies. Hydrogels, in particular, have emerged as promising tools in the fields of immunotherapy and cellular engineering, due to their high biocompatibility and modifiable physical characteristics [12]. Their soft, three-dimensional, tissue-like network structure is optimal for integrating mechanical support with biochemical functionality, while their tunable properties such as stiffness, surface chemistry, and molecular functionalization allows for precise modulation of cell behavior and coordination of complex immune responses [13,14,15]. Moreover, hydrogels have the additional benefit of allowing for targeted and site-specific therapies. Experimental treatments using hydrogels have been shown to improve therapeutic efficacy, while reducing systemic toxicity through localized and prolonged delivery of cytokines, chemokines, or antigens directly to the affected tissues [16,17,18]. Their modular design also allows for co-delivery of multiple immunomodulatory agents within a single platform, providing opportunities for synergistic and sequential stimulation in the local tissue environment [19,20,21].

In the context of this review, we focus on in vivo hydrogels that have been designed to function within living tissues, distinct from in vitro or ex vivo applications. These systems cover a broad spectrum, from injectable formulations that solidify at the target site to stimuli-responsive systems. First, we outline key hydrogel design principles, including biocompatibility, biodegradability, mechanical properties, controlled release, and immune-specific functionalization, along with considerations for translation to in vivo applications. Building on these foundational elements, we then examine strategies for modulating adaptive immunity, including approaches that promote in situ activation and functional programming. Next, we highlight methods for engaging the innate immune system, focusing on dendritic cell (DC) activation, cross-presentation, and macrophage polarization. Finally, beyond direct cellular modulation, we review hydrogels designed for targeted delivery of immunoregulatory molecules, interception of circulating tumor cells (CTCs), and reprogramming features of the TME to overcome immunosuppression.

## 2. Hydrogel Design Principles

In this section, we present several key principles for the design and application of hydrogels in the in vivo manipulation of immune responses. These include biocompatibility and biodegradability (Section 2.1), degradation kinetics (Section 2.2), and mechanical and structural properties (Section 2.3). In addition, we review controlled and spatiotemporal release (Section 2.4), injectability and in vivo gelation (Section 2.5), immune-specific functionalization (Section 2.6), and finally, design considerations for in vivo translation (Section 2.7). Together, these areas encompass the most significant considerations for adapting hydrogels to novel immunological applications.

### 2.1. Biocompatibility and Biodegradability

Biocompatibility and biodegradability are the fundamental elements in the design of hydrogels for in vivo immunomodulatory applications. Insufficient biocompatibility of a hydrogel can trigger unwanted immune activation, as the body recognizes it as foreign, leading to local inflammation, macrophage-mediated foreign body reactions, and subsequent fibrotic encapsulation [22,23,24,25]. These processes hinder the integration and function of the hydrogel, ultimately diminishing its therapeutic efficacy. Such immune-mediated risks are particularly concerning in immunologically sensitive settings such as cancer immunotherapy or autoimmune diseases, where even subtle inflammatory shifts can disrupt therapeutic outcomes. In these contexts, the immunological balance between tolerogenic and tumor-targeted immunity should be maintained. As local inflammation can undermine this balance, its precise control is essential.

Material-intrinsic properties, particularly the hydrogel’s base polymer chemistry and degradation behavior, are key determinants of such local immune responses. If a material remains in the body for an extended period, it induces ongoing immune surveillance while overly rapid degradation may release immunostimulatory byproducts or result in premature loss of scaffold structure and its intended function [26,27,28]. Immunologically inert fragments promote immune quiescence, while bioactive degradation products can act as damage-associated molecular patterns (DAMPs), activating innate immune cell receptors such as Toll-like receptors (TLRs) and initiating inflammatory cascades [29,30,31]. For instance, although hyaluronic acid (HA) is widely regarded as a biocompatible natural hydrogel and is enzymatically degradable, its degradation into low molecular weight (<500 kDa) fragments can act as endogenous DAMPs that engage TLR2 and TLR4 on DCs and macrophages, triggering NF-κB–dependent signaling and inducing pro-inflammatory cytokines, chemokines, and growth factors [32,33]. Similarly, alginate, another widely used natural hydrogel, can elicit pro-inflammatory responses depending on its purity and its mannuronic acid to guluronic acid monomer ratio [34,35]. Residual contaminants such as endotoxins or polyphenols in crude alginate have been linked to macrophage activation and secretion of pro-inflammatory cytokine, whereas highly purified alginate elicits minimal immune responses [35,36].

On the other hand, synthetic polymers like polyethylene glycol (PEG) are often described as immune-inert due to their low protein adsorption and minimal recognition by innate immune receptors [37,38,39]. PEG-based hydrogels, particularly those modified with bioactive motifs such as RGD, have been shown to reduce macrophage adhesion and fibrous encapsulation following subcutaneous implantation, thereby attenuating the foreign-body response (FBR) [40]. However, PEG is inherently non-degradable unless specifically engineered with cleavable linkers [41,42]. This characteristic raises concerns about its long-term persistence in dynamic tissues, where eventual clearance or remodeling is desirable. Moreover, repeated exposure to PEGylated materials has been associated with the formation of anti-PEG antibodies, which can lead to altered pharmacokinetics or hypersensitivity reactions [43,44]. A comparison of natural and synthetic hydrogels in terms of biocompatibility, biodegradability, degradation by-products, and polymer backbones is summarized in Table 1.

These mechanistic insights underscore the key trade-offs between natural and synthetic hydrogels. Natural polymers, especially HA and gelatin closely mimic the extracellular matrix (ECM) and are readily degraded by endogenous enzymes, facilitating cell infiltration and tissue remodeling [45,46]. However, their immunogenicity can vary with batch purity and the nature of their degradation products, sometimes leading to unpredictable immune responses [47,48,49]. In contrast, synthetic materials like PEG and PVA offer high tunability in composition, structure, and degradability, resulting in consistent immune profiles when properly engineered [50]. However, if not engineered for degradation, their prolonged persistence in tissue may result in low-grade inflammation or fibrotic encapsulation [24,51].

Application context should inform material selection. In certain contexts, such as autoimmune disease or tissue regeneration, controlled release of bioactive degradation fragments can engage the immune system, fostering tolerance or supporting regenerative processes [52,53]. In contrast, for cancer immunotherapy, where hydrogels typically serve as immune depots, minimizing unintended immune activation is paramount [52,54]. In these settings, synthetic, bioinert, and slow-degrading materials like PEG are often favored for their predictable and inert profiles [55]. Carefully purified and tailored natural materials such as HA or alginate can also be advantageous when their immunological effects are well understood and appropriately managed [56].

Ultimately, the rational design of hydrogels demands attention not only to the initial immune compatibility of the material, but also to how its properties and biological interactions evolve as it degrades and interfaces with host tissues over time.

### 2.2. Degradation Kinetics

The rate of hydrogel degradation in vivo governs the shape of the immune responses, particularly in time sensitive therapeutic contexts. Immune responses are inherently dynamic, progressing from acute activation through resolution and, in some cases, into chronic phases. For a hydrogel-based system to effectively modulate immunity, its degradation profile and thus the release of therapeutic payloads must be carefully tuned to match the desired duration and dynamics of immune modulation. If a hydrogel degrades too rapidly, it may cause a sudden burst release of encapsulated agents, overwhelming local tissues and potentially provoking excessive immune activation, exhaustion, or off-target effects [57,58]. On the other hand, a material that degrades too slowly may persist beyond its therapeutic window, risking chronic immune surveillance, fibrotic encapsulation, or impaired integration [22,59].

Rapidly degrading hydrogels are valuable in applications requiring transient immune engagement, as they deliver a short, but potent stimulatory signal sufficient to initiate robust immune priming while ensuring timely clearance to restore tissue homeostasis. Acute settings such as vaccine priming often benefit from these characteristics. For instance, in a murine A20 B-cell lymphoma model, a dextran-based hydrogel engineered to degrade within ~2 days maximized Th1 cytokine production (IL-2, IL-12, TNF-α, IFN-γ), enhanced both CD8^+^ and CD4^+^ cytotoxic T-cell activity, and achieved ~40% long-term tumor protection compared to ~10% survival with slower-degrading counterparts. These results demonstrate that carefully tuned degradation kinetics can synchronize APC activation and antigen presentation, thereby promoting robust T-cell priming [60]. Moreover, the degradation profile mirrors the natural kinetics of acute immune responses, fostering timely activation while avoiding sustained antigen exposure that can shift the balance toward tolerance, exhaustion, or chronic suppression [61,62].

Conversely, slow-degrading hydrogels are preferable for sustained immune modulation in chronic disease contexts, such as tumor immunotherapy or long-term management of autoimmune conditions. These applications may require degradation over weeks to months to maintain continuous immune engagement without repeated interventions. Such materials can persist in vivo and gradually release therapeutic payloads over this period. Specifically, a PEG-b-poly(L-alanine) injectable polypeptide hydrogel sustained the release of tumor antigens, GM-CSF, and checkpoint inhibitor antibodies aPD-1 and anti-CTLA-4 antibody over 1–2 weeks, maintaining local T-cell activation within the TME while reducing systemic exposure [19]. Similarly, slow-degrading hydrogel systems can be designed to provide sustained local release of tolerogenic factors, using polymer chemistries that support controlled and predictable breakdown, thereby helping to maintain immune homeostasis at sites of inflammation [63]. Although prolonged residence of hydrogels can increase the likelihood of fibrosis or foreign body reactions, these concerns can be mitigated by engineering degradation kinetics for predictable clearance and by thorough biocompatibility evaluation.

To overcome the limitations of static degradation rates, stimuli-responsive hydrogels have emerged as sophisticated platforms for dynamic and feedback-controlled immune modulation. These systems are engineered to undergo selective degradation in response to specific pathological cues. For instance, Zhu et al. developed a matrix metalloproteinase-2 (MMP-2) sensitive hydrogel that remained stable in normal tissue, but rapidly degraded in the presence of elevated MMP-2 activity, an enzyme involved in ECM remodeling and overexpressed in many tumors [64]. This design enabled synchronized release of its therapeutic cargos, including Bufalin, IR820, anti-PD-L1 (aPD-L1) antibody in parallel with immune cell infiltration. In a melanoma model, this protease-triggered release led to markedly enhanced intratumoral CD8^+^ T cell infiltration and suppressed tumor growth. Similarly, pH-responsive hydrogels exploit acid-labile linkers to respond to the acidic TME. Gu et al. designed an injectable hydrogel that was stable at physiological pH (~7.4) but rapidly dissolved in mildly acidic conditions (~pH 6.5) of tumors, confining the release of doxorubicin (DOX) and the BRD4 inhibitor JQ1 to the tumor site [65]. This targeted delivery induced immunogenic cell death (ICD) of cancer cells and mitigated adaptive immune resistance through localized PD-L1 inhibition. Moreover, reactive oxygen species (ROS)-responsive hydrogels incorporate oxidation-sensitive bonds that cleave in an oxidative inflammatory environment. Wang et al. reported a hydrogel that degraded in response to elevated ROS levels in the tumor, triggering the local release of the STING agonist DMXAA (5,6-dimethylxanthenone-4-acetic acid) and gemcitabine [66]. In a pancreatic cancer model, this ROS-triggered delivery boosted innate immune activation and promoted cytotoxic T lymphocyte (CTL) infiltration, amplifying the anti-tumor immune response.

Collectively, these examples highlight how stimuli-responsive hydrogels can synchronize degradation and payload release with pathological cues to maximize therapeutic impact. While these strategies enable uniquely tailored, disease responsive therapies, challenges still remain such as ensuring specificity for the pathological environment, achieving complete and safe degradation, and accounting for the variability in pathological stimuli across between patients and tissue sites. Continued advances in biomaterial chemistry, coupled with deeper insights into in vivo immune landscapes, will drive the next generation of adaptive hydrogel systems.

### 2.3. Mechanical and Structural Properties

The mechanical and structural properties of hydrogels including stiffness, mesh size, and viscoelasticity, play a central role in regulating immune cell infiltration and polarization in vivo. Immune cells, particularly macrophages and DCs, are inherently mechanosensitive and interpret these physical cues through mechanotransduction pathways that influence their morphology, cytoskeletal tension, gene expression, and functional phenotype.

Building on this framework, the ranges from soft and stiff hydrogels differently shape the immunomodulatory behavior. Defining a universal threshold for what constitutes a “soft” versus “stiff” hydrogel is not feasible, since the values vary substantially depending on the polymer system, crosslinking chemistry, and measurement mode. Instead, studies that operationally defined stiffness conditions within specific materials contexts reveal how immune responses are tuned. For example, Zhuang et al. formulated methacrylate-gelatin (GelMA) hydrogels spanning compressive moduli of ~2 (with 5% GelMa), 10 kPa (with 10% GelMA), and 29 kPa (with 15% GelMA) [67]. They found that macrophages on stiffer (29 kPa) GelMA displayed greater spreading, stronger actin cytoskeletal organization, and upregulation of pro-inflammatory cytokines (TNF-α, IL-6) and inducible nitric oxide synthase (iNOS), consistent with an M1-like phenotype. In vivo, subcutaneous implantation showed that stiffer GelMA induced fibrotic capsules with a higher fraction of M1 macrophages, whereas softer variants promoted greater macrophage infiltration but reduced capsule thickness, indicating a more pro-healing response. A more recent study by Butenko et al. directly examined wound healing with GelMA hydrogels of differing crosslinking density [68]. Lightly crosslinked (lo-GelMA, ~3 kPa) scaffolds supported robust cellular infiltration, facilitated macrophage phagocytic uptake of the material, and reduced scar formation. In contrast, highly crosslinked (hi-GelMA, ~150 kPa) scaffolds elicited sustained macrophage pro-inflammatory activation, enrichment of oxidative and fusion-prone subpopulations, and enhanced pro-fibrotic fibroblast signaling, collectively resembling a foreign-body-like response.

In the TME, stiffness exerts a distinct and immunosuppressive influence. Guenther et al. cultured myeloid progenitors on collagen- or Matrigel-coated silicone hydrogels spanning 0.2–64 kPa and found that soft condition (0.2–2 kPa) favored conventional DC (cDC) differentiation (CD80^+^, CD86^+^, Ikaros^+^), which enhanced CD4^+^ T cell proliferation and IL-2/IFN-γ secretion [69]. In contrast, stiff condition (16–64 kPa) promoted populations expressing plasmacytoid DC (pDC) associated markers (CD317, Siglec-H, TLR7), although these lacked full pDC functionality, and increased CD14^+^/CD16^+^ macrophage-like subsets with elevated phagocytic activity. Extending to 3D systems, alginate–collagen gels stiffened to ~10–20 kPa reduced CD86^+^ cDCs and enriched CD14^+^ populations relative to soft controls (<1 kPa), while dextran-based gels showed similar trends to the silicone hydrogels; softer gels (2 mM) supported CD11c^+^/CD80^+^ cDCs, and stiffer gels (9 mM) enriched CD14^+^ and TLR7^+^ subsets. Most importantly, in co-cultures with melanoma, colon, breast, lung, and pancreatic cancer cells, higher stiffness suppressed cDC development and, depending on cancer type, also limited pDCs, while consistently driving accumulation of CD206^+^ tumor-associated macrophages (TAMs) that secreted VEGFA, MMPs, and TGF-β. Collectively, these findings demonstrate that, unlike in tissue repair where stiffness primarily governs fibrosis and regenerative macrophage balance, tumor-associated stiffening redirects myeloid cells toward TAM programs that support tumor progression and dampen antitumor immunity.

**Figure 1:**
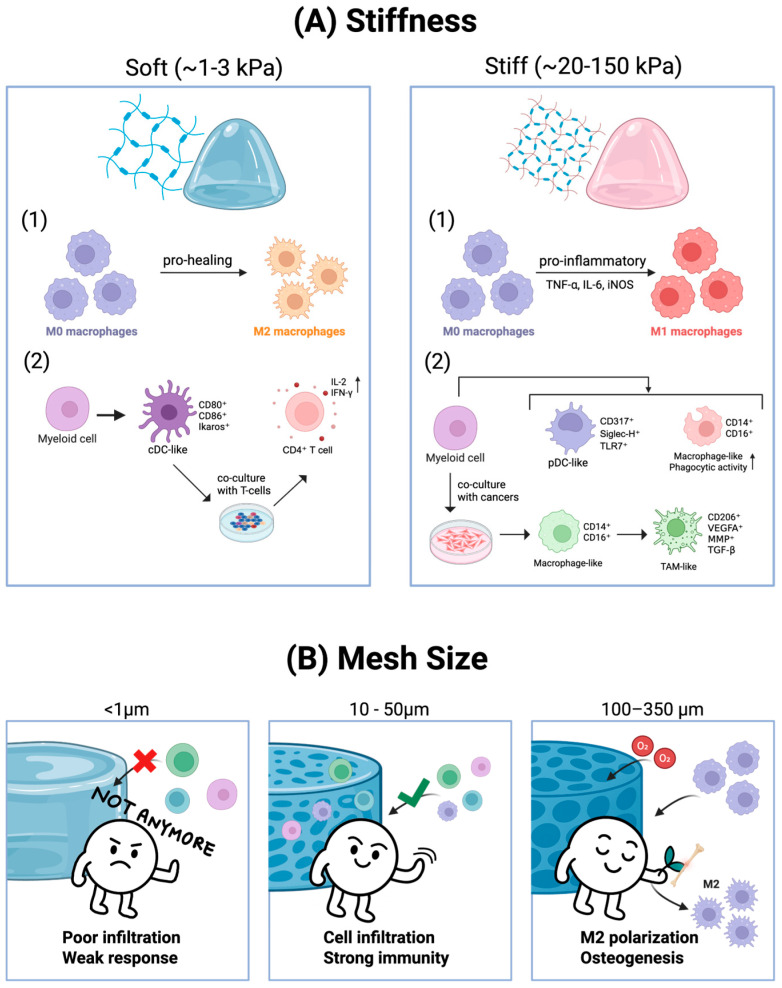
(**A**) Comparison of the effects of soft versus stiff hydrogels on cellular differentiation in (1) tissue engineering contexts and (2) tumor microenvironments. Soft: myeloid cells differentiate into cDC-like cells, promoting CD4^+^ T-cell proliferation and IL-2/IFN-γ secretion. Stiff: myeloid cells differentiate into pDC- and macrophage-like subsets with higher phagocytic activity. Co-cultures with tumor cells induces TAM-like populations supporting tumor progression. (**B**) Representation of different mesh sizes and their distinct interactions with immune cells, arranged from left to right: <1 µm, 10–50 µm, and 100–350 µm. Created in BioRender. Lanis, M. (2025) https://BioRender.com/blngrxz (accessed on 29 October 2025).

Beyond stiffness, mesh size dictates not only the degree of immune cell infiltration but also the quality of the ensuing immune response. Submicron or nanosized pore structures (<1 µm) are physically unfavorable for immune cell invasion and migration, thereby limiting activation and resulting in poor adaptive responses [70,71,72]. By contrast, micron-scale voids (10–35 µm) in MAP hydrogels enabled robust infiltration of CD11b^+^ myeloid cells and vascular-supporting cells, translating into accelerated wound healing and tissue regeneration in vivo [73]. Similarly, MAP-gel vaccines (10–50 µm pores) permitted antigen uptake by DCs and myeloid cells, which trafficked to draining lymph nodes (dLNs) and drove durable antibody responses [74]. Extending to even larger pores, pore-forming alginate hydrogels (~230 µm) loaded with GM-CSF conjugated to gold nanoparticles supported infiltration of >5 × 10^6^ cells by day 3, including >4 × 10^6^ CD11c^+^ DCs, with infiltration remaining substantial at ~2.3 × 10^6^ DCs on day 5, ~0.9 × 10^6^ on day 10, and ~0.7 × 10^6^ on day 14, thereby establishing a sustained local niche for antigen-presenting cells [75]. At the opposite extreme, regenerative scaffolds with pores exceeding 100 µm promote immune infiltration and bias macrophages toward pro-regenerative polarization, with optimal pore sizes of 100–350 µm supporting osteogenesis [76,77]. Consistently, Mukasheva et al. summarized that a pore size range of 100–400 µm is generally considered appropriate to ensure nutrient diffusion, angiogenesis, and skin regeneration, while also supporting cell attachment, proliferation, and ECM deposition in bone [78]. However, the study also reported that pores larger than 400 µm do not provide additional benefits and, in interconnected scaffolds, may even introduce structural weakness. An overview of how hydrogel stiffness and mesh size influence immune responses is illustrated in Figure 1.

While stiffness represents the static elastic modulus of a hydrogel, viscoelasticity and stress relaxation describe its time dependent mechanical behavior and how the material dissipates stress under sustained deformation [79,80,81]. These properties are critical because they determine how easily immune cells, such as macrophages and T cells, can spread and actively remodel their surrounding ECM during migration and interaction with other cells. In a fast stress-relaxing hydrogel, the network more readily yields and reorganizes in response to cellular forces, enabling macrophages to spread, extend protrusions, and adopt elongated morphologies. This environment facilitates macrophage polarization toward an M2-like, pro-regenerative phenotype, accompanied by enhanced secretion of TGF-β1, recruitment of mesenchymal stem cells, and improved osteogenesis in vivo [82,83,84]. Experimental evidence further supports the central role of stress relaxation. Chaudhuri et al. demonstrated this principle in ionically crosslinked alginate hydrogels by holding the initial elastic modulus (≈9 or 17 kPa) constant while independently tuning stress-relaxation rate. This was achieved by lowering alginate molecular weight and introducing 5 kDa PEG spacers to alter network connectivity, followed by adjusting calcium crosslinking to restore stiffness. These modifications shifted the relaxation half-time (τ_1_/_2_) from ~1 h to ~1 min without altering modulus. By decoupling relaxation from stiffness, they showed that faster-relaxing matrices promoted greater immune cell spreading, migration, and activation under otherwise identical mechanical conditions [84].

Collectively, these findings underscore that stiffness, mesh size, and viscoelasticity are not merely passive material properties but active regulators of immune cell behavior. Rational modulation of these parameters enables precise control over immune cell infiltration, activation, and polarization, offering a powerful design lever for immunomodulatory hydrogel platforms.

### 2.4. Controlled and Spatiotemporal Release

The effectiveness of immune modulation depends heavily on both the timing and localization of delivery cues. Immunological events such as antigen presentation, tolerance induction, and effector cell expansion occur over distinct temporal and spatial landscapes. For example, delivering a tumor antigen through peptide/protein vaccines, nucleic acid platforms, cell-based vaccines, or biomaterial depots followed by subsequent administration of a checkpoint inhibitor can improve T cell priming and prevent premature exhaustion [85,86,87]. Likewise, restricting immunostimulatory signals to the TME, rather than delivering them systemically, can reduce off-target inflammation and improve safety. Hydrogels are particularly useful for achieving such precise spatiotemporal control, thanks to their tunable degradation kinetics and modular architecture.

By adjusting their degradation and diffusion properties, hydrogels can provide sustained release of cytokines, antigens, or antibodies over days to weeks, maintaining therapeutic levels locally without repeated dosing. This prolonged delivery is especially significant for agents with short half-lives or dose-limiting toxicities. For instance, to overcome the rapid clearance and transient activity of cGAMP, Cheng et al. encapsulated cGAMP nanoparticles with DOX in a thermoresponsive silk-fibroin hydrogel that forms β-sheet–stabilized depots at 37 °C, enabling sustained, pH-responsive release for ~7 days and prolonged intratumoral retention (60% DOX and ~10% cGAMP at 48 h) [88]. This formulation upregulated type-I interferon genes in tumors and dLNs, and in postsurgical 4T1 models, achieved 25% disease-free survival at 70 days when combined with aPD-1 or OX40L.

Segovia et al. embedded siRNA-loaded nanoparticles in a PAMAM-dextran hydrogel and implanted it near luciferase-expressing breast tumors [89]. This localized delivery achieved ~70% gene knockdown over six days, compared to only ~20% with direct nanoparticle injection. This spatial confinement not only enhances efficacy, but also reduces toxicities. Specifically, Harui et al. showed that peri-tumoral delivery of anti-CTLA-4 using a HA-based hydrogel enabled sustained local release and achieved equivalent tumor control with one-sixth the dose in MC-38 tumor-bearing mice [90]. To illustrate the versatility of hydrogel platforms, Yang et al. developed a hydrogel co-loaded with DCs and pH-sensitive DOX/CpG nanoparticles, which was injected intratumorally into B16-bearing mice [91]. The formulation showed sustained in vivo fluorescence of DOX for up to seven days, whereas free nanoparticles were rapidly cleared. Mice treated with the hydrogel maintained stable body weight, suggesting lower systemic toxicity. In addition to continuous release, hydrogels can also be engineered for pulsatile or on-demand release, using stimuli-responsive linkages sensitive to ultrasound, heat, or pH. These strategies allow immunomodulators to be released in synchrony with immune dynamics, which can enhance T cell expansion and memory formation [92,93,94].

Notably, these temporally and spatially patterned deliveries not only provoked strong local immune activation but also drove systemic antitumor responses. By sustaining immune stimulation at the tumor site, hydrogel platforms promote efficient antigen uptake, DC activation, and downstream T cell priming. In summary, by controlling when and where immunomodulators are released, hydrogel platforms offer a rational strategy to shift the TME from immunosuppressive to immunostimulatory while improving both efficacy and safety of in vivo cancer immunotherapy.

### 2.5. Injectability and In Vivo Gelation

**Figure 2:**
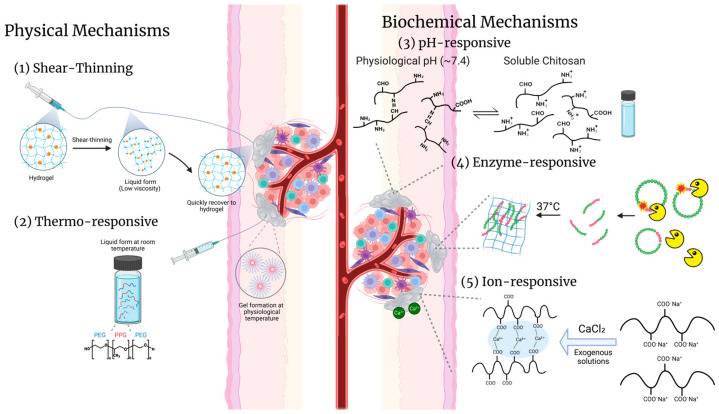
Schematic representation of delivery strategies for in vivo gelling hydrogels, grouped by mechanism. Physical mechanisms, including (1) shear-thinning and (2) thermo-responsive, are shown on the left, while biochemical mechanisms, including (3) pH-responsive, (4) enzyme-responsive, and (5) ion-responsive, are shown on the right. Created in BioRender. Lanis, M. (2025) https://BioRender.com/r4bm12r (accessed on 29 October 2025).

A central challenge in modern biomaterials design is achieving safe and targeted delivery of therapeutic agents. Injectable hydrogels that undergo in situ gelation, forming their gel structure under physiological conditions at specific sites such as lymph nodes, tumors, or inflamed tissues, have addressed this challenge by enabling minimally invasive administration and localized retention. The other approach involves implantable hydrogels, which are engineered as pre-formed scaffolds surgically placed into target tissues. This strategy provides precise structural control, long-term stability, and tunable degradation, but inherently requires invasive implantation, which can provoke inflammation and limits adaptability for repeated dosing [65,75]. In contrast, injectable hydrogels can be delivered via syringe, minimizing tissue trauma, reducing recovery time, and facilitating repeated dosing if needed [75]. Upon injection, the hydrogel forms a localized depot at the target site, retaining immunomodulators locally and limiting their diffusion into systemic circulation. This combination of injectability and site-specific gelation offers an effective patient-friendly strategy for immune modulation. The different in vivo gelling mechanisms are illustrated in Figure 2.

#### 2.5.1. Physical Injectability Mechanisms

The physical properties that enable injectability without compromising gel formation are central to the performance of these systems. Among the diverse approaches, two design strategies are most widely adopted: (1) shear-thinning hydrogels, which temporarily reduce viscosity under applied stress and rapidly recover their structure afterward, and (2) thermoresponsive hydrogels, which undergo solution-to-gel transitions upon exposure to physiological temperature. Both approaches eliminate the need for in situ chemical crosslinking, simplifying formulation and improving biocompatibility, while allowing precise, localized delivery of therapeutic payloads.

Shear-thinning hydrogels are formulated with physically crosslinked polymer networks that dissociate under applied shear stress and rapidly reassemble when the stress is removed. This property allows the hydrogel to pass through narrow-gauge needles while minimizing patient discomfort, procedural risk, and recovery time. Upon injection, the material flows as a liquid but quickly recovers to a gel-like state at the target site, forming a localized depot. A representative example is the gelatin–Laponite nanocomposite hydrogel developed by Falcone et al., which was loaded with the chemotherapeutic DOX and the ICI antibody aPD-1. These supramolecular networks disassemble under shear and rapidly reassemble at the target site once the shear is removed, enabling self-healing and precise local injection. Rheological analysis showed storage moduli (G′) in the range of ~200–400 Pa, with G′ consistently higher than the loss modulus (G″), meaning that the hydrogel behaves primarily as an elastic solid rather than a viscous liquid under small deformations. The hydrogel exhibited pronounced shear-thinning, as viscosity decreased by nearly two orders of magnitude, from ~10^3^ Pa·s at low shear rates to ~10 Pa·s at high shear rates. Under cyclic deformation tests alternating between 1% and 100% strain, the network structure broke down at high strain but rapidly recovered its baseline storage modulus (~200–300 Pa) within seconds once the strain was removed, indicating robust self-healing. In vivo, this drug-eluting shear-thinning hydrogel enabled pH-responsive release of DOX and sustained release of aPD-1, resulting in significantly reduced tumor volumes, higher apoptotic indices, increased CD8^+^ T cell infiltration, and improved survival in a mouse hepatocellular carcinoma (HCC) model [95].

Thermogelling polymers utilize temperature induced phase transition to achieve injectability. These systems are liquid at room temperature and undergo gelation upon warming to physiological temperature. Many thermoresponsive polymers exhibit a lower critical solution temperature, above which polymer chains dehydrate and form physical crosslinks through increased polymer–polymer interactions. For example, poloxamer 407 (P407) at 18–23% (*w*/*w*), which is a range commonly used in pharmaceutical applications, remains liquid at ~20–25 °C but rapidly gels at 37 °C [96]. Notably, the gelation temperature can be easily tuned by adjusting polymer concentration. At higher concentrations (25–30%), it can drop to as low as 10–14 °C [97]. In a CT27 colon cancer mouse model, Crux et al. used a 25% P407 formulation for CTLA-4 antibody delivery, achieving significant tumor growth inhibition without cytotoxicity [96]. Similarly, Chen et al. developed thermosensitive PEG–PLGA–PEG triblock copolymers for sustained Herceptin delivery, suppressing HER2^+^ breast tumor progression [98]. These studies demonstrate how thermogelling systems integrate minimally invasive administration with sustained, localized release of biologics for cancer immunotherapy.

Importantly, both shear-thinning and thermosensitive strategies allow for minimally invasive hydrogel delivery without requiring external crosslinkers or initiating chemical reactions in vivo, which simplifies formulation and preserves the bioactivity of encapsulated therapeutics. Once administered, these hydrogels remain localized at the injection site, forming a viscoelastic depot that retains therapeutic agents locally and supports sustained, site-specific release.

#### 2.5.2. In Vivo Gelation via Biochemical Triggers

In addition to physically triggered mechanisms, in vivo hydrogel gelation can also be achieved through biochemical cues present in the target tissue microenvironment. These systems rely on endogenous triggers such as pH shifts, ionic gradients, or enzymatic activity to initiate sol–gel transitions after injection. Their ability to undergo gelation specifically at pathological sites, without the need for external stimuli or surgical intervention, makes them particularly valuable in inflamed, ischemic, or tumor tissues. For instance, pH- and ion-responsive hydrogels can exploit the acidic or hypertonic conditions of diseased tissues, while enzyme-sensitive systems respond to local protease activity. These mechanisms improve the precision of hydrogel localization and therapeutic retention, further broadening the scope of injectable platforms for targeted delivery.

Some injectable hydrogels are designed to gel in response to physiological or pathological pH changes. These systems exploit ionizable functional groups (e.g., carboxyl or amine moieties) that undergo protonation or deprotonation depending on local pH. This change can either induce phase transition gelation or activate functional group reactivity that leads to covalent network formation. For example, chitosan is a polyamine that is soluble under mildly acidic conditions, allowing injection as a liquid. Upon exposure to physiological pH (~7.4), its amine groups deprotonate, resulting in gelation in vivo [99]. In another system, N-carboxyethyl chitosan and dibenzaldehyde-terminated PEG form a dynamic imine-linked hydrogel at neutral pH. The rapid sol–gel transition is driven by the reaction between free amine groups on the chitosan and aldehyde groups on the dibenzaldehyde-terminated PEG [100]. In addition, these pH-sensitive hydrogels can be formulated to degrade more rapidly in acidic environments, such as inflamed or tumor tissues. This property accelerates drug release at disease sites [101,102]. Overall, the underlying mechanism relies on endogenous pH gradients to trigger in situ gelation and localized therapeutic delivery, without the need for external stimuli.

A more cell responsive form of biochemical triggering involves hydrogels that respond to enzymatic activity, particularly MMPs present in the tissue. While most MMP-sensitive hydrogels are designed for enzyme-triggered degradation to facilitate drug release, some systems use MMP activity as a direct trigger for gelation. In these designs, the hydrogel’s crosslinks or backbone incorporate peptide sequences cleavable by MMPs, so that the material’s gel state can be locally remodeled by cell-secreted enzymes [103]. For instance, Yang et al. designed a peptide-based pro-gelator containing an MMP-9 cleavage site. Upon enzymatic cleavage, the liberated segment self-assembled into a supramolecular hydrogel [104]. Furthermore, Carlini et al. developed cyclic peptide progelators that remain inert in solution until cleaved by MMP-2, MMP-9, or elastase. Once cleaved, the linearized peptides rapidly formed viscoelastic networks capable of supporting localized gel formation [105]. Extending beyond proteases, Coulter et al. demonstrated a peptoid–peptide pro-gelator in which phosphotyrosine serves as an enzymatic switch. Upon dephosphorylation by endogenous phosphatases after subcutaneous injection, the liberated molecule self-assembles into a fibrous hydrogel network within ~30 s, with gel growth continuing over ~90 min. The hybrid peptoid/peptide design enhanced stability via D-residues and peptoid blocks. Also, this hydrogel acted as a long-acting drug depot as the antiretroviral zidovudine was covalently conjugated to the pro-gelator via an ester bond, enabling controlled hydrolytic release for over 30 days in rats [106]. This enzymatically instructed assembly not only eliminated the need for chemical initiators or exogenous crosslinkers but also provided sustained therapeutic exposure, reduced burst release, and improved systemic pharmacokinetics compared to free drug administration.

In contrast to pH- and enzyme-responsive systems, where biochemical cues alone can directly trigger sol–gel transitions in vivo, ionic crosslinking generally cannot rely solely on endogenous ions to achieve robust hydrogel formation. Alginate, a classical example, is an anionic polysaccharide that undergoes rapid gelation upon encountering divalent cations like Ca^2+^ [107]. However, free Ca^2+^ in the extracellular fluid is typically maintained at ~0.9–1.2 mM, which is insufficient for forming stable networks [108,109]. Experimental studies have shown that gels formed at 10 mM CaCl_2_ are weak and may dissolve under physiological conditions, whereas stable scaffolds with optimal properties are often obtained only at much higher concentrations, up to 500 mM [110,111]. Consequently, most ion-responsive strategies, particularly calcium-responsive hydrogels, achieve in situ gelation through either the co-administration of exogenous Ca^2+^ solutions, or the incorporation of internal Ca^2+^ reservoirs such as CaCO_3_/GDL systems. Ferreira et al. demonstrated that alginate-based injectable hydrogels crosslinked with co-injected CaCl_2_ could locally deliver the monoclonal antibody bevacizumab, resulting in significant suppression of angiogenesis and improved intratumoral retention [112]. Similarly, Chao et al. reported alginate composites that rapidly formed hydrogels upon the addition of Ca^2+^ and served as sustained-release depots for oxaliplatin and the immune adjuvant R837, which synergized with systemic aPD-1 therapy to promote durable antitumor immunity. This ionotropic gelation mechanism leverages simple ionic interactions to form networks in vivo, thereby avoiding radical initiators or chemical crosslinkers [113]. Alternatively, other polysaccharides can also undergo in vivo gelation through ionic interactions. For instance, low-methoxyl pectins form gels via calcium-mediated crosslinks, following the ‘egg-box’ model, in the presence of divalent cations such as Ca^2+^ [114]. Similarly, κ-carrageenan forms strong, rigid gels when exposed to potassium ions, which stabilize junction zones between helical chains [115]. In addition, polyelectrolyte complex gels can be generated in situ by co-injecting oppositely charged polymers, such as cationic chitosan and anionic alginate, which then spontaneously assemble into stable networks through electrostatic interactions [116,117,118].

Beyond pH, protease, and ionic-triggered gelation, other biochemical stimuli have also been exploited to create smart in situ-forming hydrogels. For instance, redox-responsive systems incorporate disulfide bonds or ROS-cleavable linkages that respond to in vivo reducing or oxidative conditions, such as high glutathione levels inside cells or inflammatory ROS [119,120]. Glucose-responsive hydrogels have also been developed, often utilizing phenylboronic acid-diol interactions or enzyme-mediated feedback (e.g., glucose oxidase yielding local pH changes) to trigger gel swelling or drug release in the presence of elevated glucose [121,122,123]. While these are outside the main categories, they highlight the rich variety of biochemical triggers that researchers have harnessed to achieve in situ gelation and responsive drug delivery in vivo. Together with physically triggered injectability mechanisms, these approaches broaden the toolbox for designing injectable hydrogels that solidify or adapt under specific biological conditions.

### 2.6. Immune Specific Functionalization

While the physical properties and in vivo gelation behavior of hydrogels critically influence immune cell infiltration and material retention, these passive features alone are sometimes insufficient to achieve specific, robust, or cell-type selective immunomodulatory effects. To enhance immune engagement, many hydrogel systems have been functionalized with immunologically active ligands to promote selective cell interactions, receptor-mediated signaling, and in some cases, mimic antigen presentation. Such functionalization transforms hydrogels from inert delivery depots into instructive microenvironments capable of directing immune cell behavior at the molecular level.

Immune cells including macrophages, DCs, and T lymphocytes express integrin receptors that recognize specific biochemical motifs within the ECM. For example, the Arg–Gly–Asp (RGD) sequence, present in fibronectin, vitronectin, and osteopontin, engages a subset of β1 and β3 integrins to modulate immune cell activation. In DCs, RGD binding to αvβ3 integrins promotes maturation, marked by increased expression of major histocompatibility complex (MHC) class II and CD86 molecules on the cell surface, along with elevated IL-12 secretion [124]. The laminin-derived pentapeptide IKVAV (Ile-Lys-Val-Ala-Val), found in laminin-1, interacts with integrins such as α_2_β_1_ on macrophages and has been shown to influence their polarization [125]. These responses are highly sensitive to the type, density, and spatial presentation of matrix ligands, highlighting the immune system’s responsiveness to subtle microenvironmental cues.

For instance, RGD-functionalized hydrogels translate this integrin-mediated sensitivity into selective immune modulation. The RGD hydrogels selectively recruited reparative macrophage subsets (CD206^+^) while scrambled RDG controls preferentially recruited DCs. Beyond shifting lineage balance, RGD motifs also altered CX3CR1^+^ myeloid cell migration, skewed cytokine profiles toward chemotactic and reparative factors (VEGF, MCP-1, MIP-1α/β), and constrained macrophage polyfunctionality into defined effector and regulatory outputs, collectively fostering a pro-regenerative and pro-angiogenic environment [126].

Beyond adhesive motifs, hydrogels have also been directly functionalized with immunoregulatory cytokines. Beskid et al. developed PEG-4MAL hydrogels covalently tethered with IL-10 (PEG-IL10) to create a tolerogenic delivery platform for DCs. Incorporation of IL-10 extended DC viability compared with unmodified gels and preserved an immature, immunosuppressive phenotype even after exposure to strong inflammatory stimuli such as LPS or TNF-α/IFN-γ. DCs maintained in PEG-IL10 hydrogels resisted upregulation of the costimulatory marker CD86, demonstrating protection from maturation. In coculture assays, PEG-IL10–conditioned DCs induced significantly higher frequencies of CD25^+^FoxP3^+^ regulatory T cells (Tregs) and increased PD-1 expression on CD4^+^ and CD8^+^ T cells. Moreover, cytokine profiling revealed elevated IL-10 in supernatants and reduced proinflammatory responses, indicating that the hydrogel niche not only stabilized tolerogenic DCs but also reprogrammed surrounding lymphocytes toward an immunosuppressive state [127]. A more complex example is the use of microcavity-containing hydrogel microparticles (nanovials) functionalized with EABR-mediated vesicles that display full-length membrane antigens (e.g., NYESO1–pMHCI or HER2) embedded in native lipid bilayers. By more closely replicating the molecular organization of the immunological synapse than recombinant soluble ligands, these vesicle-coated nanovials increased the capture of NYESO1-specific 1G4 T cells from ~25% to over 50% of nanovials and enhanced their IFN-γ secretion nearly two-fold. Similarly, for HER2 genetically modified T cells, vesicle-coated nanovials boosted functional activation from ~9% to ~25% IFN-γ^+^ cells, representing more than a two-fold improvement over recombinant HER2 controls [128].

**Figure 3:**
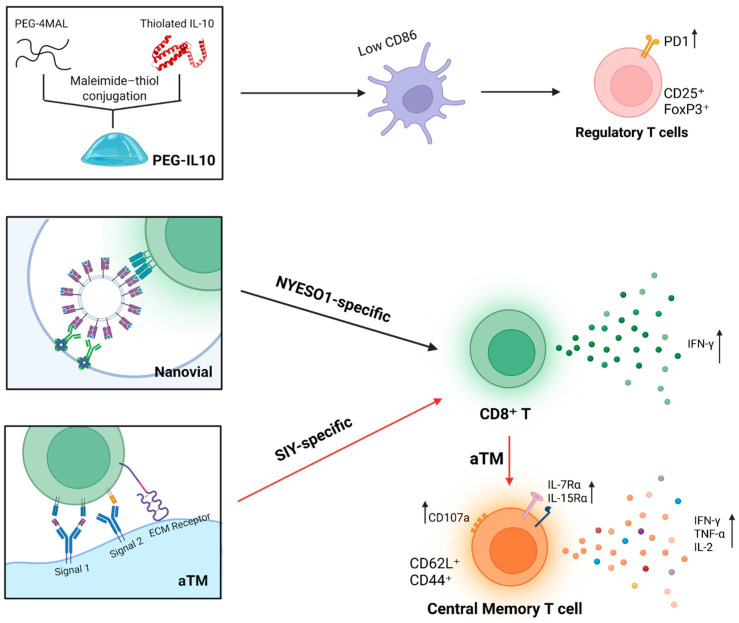
Functionalized hydrogels with distinct ligands interacting with different immune cell types. From top to bottom, PEG-4MAL hydrogel functionalized with IL-10; microcavity-containing hydrogel microparticles functionalized with EABR-mediated vesicles displaying full-length membrane antigens; and HA hydrogel functionalized with immune-modulatory ligands serving as an artificial T-cell–stimulating matrix. Created in BioRender. Lanis, M. (2025) https://BioRender.com/phg7oxo (accessed on 29 October 2025).

Another strategy involves direct conjugation of immune-activating ligands to hydrogel matrices to mimic APC signals. Hickey et al. engineered an artificial T-cell stimulating matrix (aTM) by tethering T cell activating molecules, peptide–MHC (Signal 1) and anti-CD28 (Signal 2), to a soft HA hydrogel. This design combines ECM-mediated cues with localized receptor engagement to optimize mechanosensitive T cell receptor (TCR) signaling. In preclinical models, aTMs expanded SIY-specific CD8^+^ T cells more than fourfold compared to conventional stimulation and promoted features associated with a central-memory phenotype (CD62L^+^CD44^+^) along with increased IL-7Rα and IL-15Rα expression. aTM cultures also generated multifunctional effector cells co-producing IFN-γ, TNF-α, and CD107a (with IL-2 production observed in related HA hydrogel conditions). Adoptive transfer of these cells into B16-SIY melanoma-bearing mice significantly delayed tumor growth and resulted in 66% survival at day 40 [129]. The structures of PEG-IL10 hydrogels, nanovials, and aTMs, and their interactions with different immune cells, are illustrated in Figure 3.

These examples illustrate not only the breadth of immune-specific functionalization strategies but also their capacity to precisely tune interactions with distinct immune cell subsets and activation profiles. These cases represent only a selection of the diverse strategies reported, which also include cytokine-tethering, immune checkpoint ligand display, and multivalent co-stimulatory architectures. Collectively, these findings underscore a core design principle: by integrating immunologically active ligands into hydrogel matrices, inert scaffolds can be transformed into instructive microenvironments capable of directing immune responses toward defined therapeutic goals.

### 2.7. Design Considerations for In Vivo Translation

Functionalized hydrogels are advancing beyond preclinical studies, with several formulations already tested in early clinical trials for cancer, fibrosis, and vaccine delivery. These achievements highlight their potential as versatile immune-modulating platforms. Still, broader clinical translation will require careful engineering beyond basic functionality. Key priorities include establishing reproducible large-scale manufacturing, ensuring safety and regulatory compliance, and tailoring hydrogel properties to the biological and mechanical features of the target tissue microenvironment.

Sterilization represents a particular challenge, since hydrogel formulations must be processed without compromising ligand integrity or bioactivity. Many immunoactive components, including growth factors and antibodies, are sensitive to high heat or ionizing radiation from methods such as gamma irradiation, electron beam (e-beam), filtration, or autoclaving, which are typically required for clinical advancement [130]. One study reported that 30–35 kGy e-beam and gamma sterilization did not significantly alter the swelling ratio, mechanical properties, or tribological performance of a cartilage-like hydrogel. However, minor chemical changes were still observed under e-beam, and because this finding was limited to a specific hydrogel type, sterilization-induced alterations remain a continuing barrier for translation [131].

In parallel, scalable manufacturing requires consistent ligand density, crosslinking degree, and mechanical properties across production batches through rigorous process control. The feasibility of large-scale production also depends on securing a stable supply of clinical-grade raw materials and ensuring compatibility with Good Manufacturing Practice protocols. During scale-up, it is critical that reaction parameters and material homogeneity be tightly maintained to preserve reproducibility across batches [132,133,134,135].

Beyond manufacturing, regulatory classification introduces further obstacles. Hydrogels containing biologically active agents such as antibodies, peptides, or cytokines may be regulated as drug–device combination products rather than standalone medical devices, imposing dual requirements for pharmaceutical and device evaluation [136,137]. Endotoxin contamination, referring to lipopolysaccharides and other bacterial products capable of eliciting strong immune responses during manufacturing, should be minimized and remain below regulatory thresholds (e.g., ≤5 EU/kg for injectable products), typically verified by Limulus Amebocyte Lysate assays [138,139].

Additional impurities, including residual bacterial expression products and host cell proteins (HCPs), also require strict removal during manufacturing. For example, Jawa et al. demonstrated that even high HCP levels (up to 4000 ppm) can raise concerns during purification process development, underscoring the importance of impurity control. Preclinical safety testing should therefore encompass both local and systemic immune responses, degradation product biocompatibility, and risks of chronic inflammation or off-target activation [140]. As highlighted by Rezaei et al., hydrogels are expected to minimize persistent immune activation and reduce foreign body reactions that might otherwise progress to fibrous encapsulation or chronic inflammation [141].

Finally, the functional design of hydrogels should be matched to the mechanical, biochemical, and immunological context of the target tissue. In skin applications, hydrogels require sufficient strength to withstand shear and stretching while incorporating strategies to traverse the stratum corneum, such as microneedles, permeation enhancers, or nanoscale carriers, and can exploit the abundance of resident antigen-presenting cells [45,142]. Tumor-associated environments, by contrast, feature increased ECM stiffness, elevated protease activity, hypoxia, and profound immunosuppression; hydrogels for these sites benefit from protease-responsive degradability while maintaining structural integrity for sustained release [45]. Mucosal tissues, with rapid epithelial turnover, continuous mucus secretion, and dynamic microbiomes, demand rapid gelation, long-term hydration retention, and strong mucoadhesion [143,144]. In each of these contexts, immune cell composition and activation profiles are distinct, meaning that hydrogel design must account not only for tissue mechanics but also for the ensemble of immune signals present at the site—such as Langerhans cells in skin, suppressive myeloid subsets in tumors, or tolerance-inducing pathways at mucosal barriers [45,144]. Ultimately, successful translation will depend not only on material design and manufacturing control but also on integration with clinical delivery modalities, patient variability, and long-term safety monitoring.

## 3. Cellular Modulation

The immune system operates as a tightly regulated network that must respond rapidly to threats, while avoiding excessive or misdirected responses that could damage healthy tissues. This balance is maintained through tightly coordinated signaling pathways, spatial organization of immune niches, and feedback mechanisms that modulate cell activation, proliferation, and eventual downregulation [1]. In the context of cancer, malignant cells evolve mechanisms to evade or suppress cellular immune responses by exploiting the existing regulatory pathways, thereby allowing tumors to persist and progress within an otherwise immunocompetent host [145]. As a result, immunotherapies must strategically harness distinct immune cell populations to counteract tumor immune escape mechanisms. Here, we discuss hydrogel strategies designed to influence T cells (Section 3.1), DCs (Section 3.2), and macrophages (Section 3.3), highlighting how these approaches enhance immune activation, persistence, and functional programming within the tumor context.

### 3.1. T Cells

T lymphocytes, particularly cytotoxic CD8^+^ and helper CD4^+^ T cells, are central orchestrators of effective anti-tumor immunity. In order to carry out their effector functions, these cells depend on a multistep activation process that governs their expansion, differentiation, and functional programming. In brief, T cells require three key signals for activation. Signal 1 is delivered through engagement of the TCR, which binds to disease-specific peptides presented on MHC molecules on the surface of APCs. Notably, CD8^+^ T cells respond only to peptides presented on MHC class I molecules, while CD4^+^ T cells are restricted to peptides presented on MHC class II. Signal 2 involves co-stimulatory molecules, such as CD28 on T cells binding to CD80/CD86 on APCs, which amplify the intracellular activation signaling cascade. T cells that receive Signal 1 in the absence of Signal 2 are known to become anergic, due to incomplete activation of downstream transcriptional programs necessary for effector differentiation. Signal 3 is provided by cytokines in the microenvironment, which shape T cell fate by influencing their overall proliferation, survival, and effector differentiation [146]. Lymphocytes primarily receive all three of these signals in a secondary lymphoid organ such as the lymph nodes and spleen where the highly organized tissue environment facilitates effective APC and T cell interactions [147,148]. Upon receipt of these activating signals, T cells proliferate, traffic to target tissues, and carry out effector functions such as direct tumor cell killing, cytokine secretion, and support other immune cells [149].

#### 3.1.1. Delivery of Activated T Cells

Given the critical role of T cells in orchestrating anti-tumor responses, ACT has emerged as a clinically powerful approach that harnesses and amplifies these cells for therapeutic use. In ACT, T cells are isolated, expanded, and sometimes engineered ex vivo, and reinfused into the patient to enhance tumor targeting and destruction. ACT has demonstrated remarkable clinical efficacy in hematologic malignancies and as of August 2025, there are eight FDA-approved T cell products available [150]. At present, four standard ACT treatment strategies exist including transfer of CTLs, tumor-infiltrating lymphocytes (TILs), T cell receptor-modified T cells, and chimeric antigen receptor (CAR) T cells [151]. Of these, CAR T cell therapies have received the most FDA approvals and the most attention in hydrogel-based delivery research [150,152,153,154,155,156,157]. CAR T cells are genetically engineered to express synthetic receptors that combine an extracellular antigen-binding domain, typically derived from a monoclonal antibody, with intracellular T cell signaling domains that activate the T cell upon antigen engagement. Unlike TCRs, which recognize antigenic peptides presented by MHC molecules, CARs allow T cells to recognize surface antigens on tumor cells in an MHC-independent manner, bypassing challenges associated with rare-tumor antigen presentation and the scarcity of antigen-specific T cells. Unfortunately the broader application of ACT, particularly in solid tumors, remains limited by barriers such as poor persistence and the immunosuppressive TME [158]. Issues pertaining to restricted tumor infiltration are particularly important in solid tumors, where intravenously infused T cells often fail to adequately traffic to or persist within the tumor bed, limiting their therapeutic efficacy.

Hydrogels offer a promising delivery strategy that can protect T cells during ACT administration, promote local retention and expansion, and provide a supportive microenvironment that enhances T cell survival and function. For example, dual delivery of CAR T cells with cytokines in a supporting hydrogel matrix has been used in multiple studies to improve longevity, expansion, and anti-tumor activity [152,154,159]. In one such approach, polymer-nanoparticle hydrogels (PNPs) were fabricated by combining PEG-polylactic acid nanoparticles with a solution of dodecyl-modified hydroxypropyl methylcellulose (HPMC-C12) [154]. This self-assembling hydrogel was found to non-specifically adhere to the pro-inflammatory cytokine IL-15 and thus inhibit its diffusion, while still allowing for cellular movement through its matrix. Researchers leveraged this property to enhance stimulation of B7H3-specific CAR T cells by co-administrating the cells and the cytokine via the PNP into a murine solid tumor model. Later analysis revealed enhanced CAR T cell proliferation and persistence at the tumor site, as well as greater proportions of CD8s and T stem cell memory subsets when compared to injection of hydrogel alone. Furthermore, mice treated with hydrogel-encapsulated CAR T cells and IL-15 exhibited significantly improved survival compared to those receiving the same treatment IV.

An additional benefit of hydrogel-mediated CAR T cell delivery is its potential to reduce processing time. Currently, manufacturing CAR T cell products for clinical use can take between three and six weeks, delaying treatment for patients with rapidly progressing disease. Accordingly, strategies designed to enhance expansion could help to shorten production timelines and enable more timely therapeutic intervention [160]. Jie et al. were able to affect overall proliferation of CARs by modulating the stiffness and adhesive ligand density of their nanofiber hydrogel, a technique also used with non-genetically modified T cells [129,157]. They additionally engineered their cells to secrete a PD-1 blocking scFV, which generated a >12-fold increase in proliferation over cells cultured in media when combined with the optimal stiffness and ligand density. In the end, this self-assembling scaffold reduced ex vivo culture time to just three days, with the resulting cells showing significant tumor suppression after subcutaneous injection in the matrix compared to standard CAR-T infusion.

Beyond CAR T cell delivery, hydrogels have been employed as delivery vehicles for naturally occurring T cell populations with intrinsic anti-tumor activity, such as TILs. One group used a microfluidic chip to encapsulate CD8^+^PD-1^+^ T cells in alginate gel droplets containing non-specific stimulating signals, creating a supportive environment that promoted rapid proliferation without inducing differentiation [161]. Although PD-1 is commonly associated with T cell exhaustion, its expression on TILs, particularly in melanoma, can indicate tumor reactivity and overall function can be recovered after culture with IL-2 [162,163]. To evaluate in vivo efficacy of the cells, mice were inoculated with B16-tumor cells on day 0 and subsequently treated on days 7, 12, and 17 with one of four regimens: gel anti-tumor T cell injections (GATI), T cells from a culture flask, gel-encapsulated adjuvant alone, or PBS. By day 35, GATI-treated mice exhibited 100% survival with no detectable tumors, while all other groups showed complete mortality.

Hydrogel-based delivery has also been combined with antigen-specific technologies to generate and administer autologous CTLs. Li et al. [164]. used nano-artificial antigen-presenting cells bearing pMHC-I and anti-CD28 in the presence of polarizing cytokines (IL-15, TGF-β) to expand tissue-resident memory-like CD8^+^ T cells, which have been linked to improved clinical outcomes [165,166,167]. These cells were then embedded in injectable hydrogels formed from acrylated HA and cross-linked with thiolated PEG. Upon administration in a murine tumor model, the hydrogel supported potent local and systemic anti-tumor responses. Cui et al. used a similar strategy when they extracted effector memory T cells (CD8^+^CD62L^−^) from mice previously inoculated with B16-F10 [168]. These cells were then mixed with 25% *w*/*v* Pluronic F-127 and placed at 37 °C to gelate. After two rounds of treatment in a murine B16 tumor model, the T cell loaded hydrogel treatment group showed significantly lower tumor volumes and higher percentages of CD8^+^ T cells in the TME compared to controls.

Notably, while hydrogels have been successfully applied for the delivery of CAR T cells, TILs, and CTLs, there are currently no reports of their use in delivering TCR-engineered T cells. Given the parallels in manufacturing and delivery challenges across ACT modalities, adapting hydrogel systems for TCR-based therapies could represent an important avenue for future investigation.

#### 3.1.2. In Situ Activation

Hydrogel-based platforms have also been engineered to function as immunostimulatory niches capable of activating T cells directly in vivo. Rather than delivering large numbers of pre-expanded cells like in traditional ACT, these systems present activation cues within a localized scaffold that supports in situ T cell priming, avoiding complications associated with lengthy ex vivo stimulation. In vivo activation strategies using hydrogels generally fall into two categories: (1) systems designed to activate naïve T cells co-delivered into the scaffold and (2) platforms that deliver activating signals to the endogenous T cell population [92,169,170,171,172,173].

One example of in situ activation using co-delivered naïve T cells is the T cell-responsive macroporous hydrogel developed by Bhatta et al. [92]. This platform embeds anti-CD3/anti-CD28 Dynabeads within a degradable gel network composed of disulfide-crosslinked polymers. When T cells are loaded into macropores of the scaffold, they interact with the disulfide bonds, locally degrading the hydrogel and releasing the embedded Dynabeads into the macropores as well. As the Dynabeads are gradually released from the matrix, the T cells become stimulated and proliferate in a controlled manner in vivo. In a murine tumor model, hydrogel-treated mice exhibited antitumor responses comparable to those receiving intravenous Dynabeads and T cells, despite receiving a five-fold lower T cell dose. In a similar strategy, Livingston et al. activated adoptively transferred naïve T cells in situ using a customizable HA-based hydrogel covalently bound to activating Signals 1, 2, and 3 [169]. By tuning individual signal densities, the researchers were able to generate a formulation that produced fourfold more T cells than Dynabeads. Additionally, the versatility of the platform in presenting different ligands for Signals 1, 2, and 3 enabled antigen-specific expansion of rare murine T cells in vivo, as well as human T cells in vitro, highlighting its translational potential.

Beyond systems that co-deliver and activate naïve T cells, an increasing number of hydrogel-based platforms are being designed to engage endogenous T cells in situ and bypassing adoptive transfer altogether. These approaches rely on the localized presentation of immunostimulatory cues to prime and expand host T cells. In one such approach, “training court” hydrogel microspheres (MS-ITC) were injected directly into tumors, where they engaged TILs through surface-bound anti-CD3 and anti-CD28 antibodies while simultaneously releasing IL-7 and IL-15 to support T cell survival and expansion [172]. Taking this idea one step further, a separate group designed a spatially structured, biomimetic hydrogel scaffold capable of attracting T cells to the injection site rather than relying on pre-existing TILs [171]. By compartmentalizing its cargo, the scaffold enabled timed release of a chemoattractant to recruit T cells, followed by delayed release of anti-CD3 and anti-CD28 to drive their activation. In both of these systems, researchers were able to demonstrate enhanced anti-tumor responses in murine tumor models, as evidenced by reduced tumor growth.

Other groups have continued to build on this strategy by using injectable hydrogel formulations to deliver bispecific T cell engagers (BiTEs) for both activation and targeting. BiTEs are synthetic antibody constructs that simultaneously bind CD3 on T cells and a tumor-associated antigen on cancer cells, thereby directing polyclonal T cell populations to mediate tumor cell killing. Unfortunately, the clinical utility of BiTEs is often limited by their short in vivo half-life, which can reduce therapeutic efficacy and necessitate continuous infusion or repeated dosing [174]. Two recent hydrogel platforms have addressed this challenge by incorporating BiTEs into injectable scaffolds to recruit and activate T cells against tumor cells, while simultaneously extending the half-life of the BITE itself. One such system employed nonshrinkable, thermosensitive copolymers composed of PLGA and PEG (DTgel) to achieve controlled, sustained release of anti-CD3/anti-EGFR BiTEs [173]. Compared to intravenous administration, various formulations of DTgel prolonged BiTE half-life by 2- to 3.5-fold, and enhanced antitumor activity including increased T cell infiltration and reduced tumor volume in murine models. In a follow-up study, the same group developed D2gel, a simplified PEG-PLGA formulation that retained the functional benefits of DTgel while being easier to manufacture [170]. When loaded with anti-PSMA/CD3 BiTEs and administered subcutaneously, both DTgel and D2gel formulations significantly reduced tumor burden after 21 days relative to intravenous and subcutaneous injection. Moreover, tumor inhibition closely correlated with BiTE mean residence time, and gel-treated mice exhibited the greatest amounts of T cell infiltration in both the tumor and tumor-draining lymph nodes (TDLNs). Altogether these studies demonstrate that hydrogel-based delivery of immunomodulators can serve as a powerful alternative to cell transfer-based immunotherapies.

A particularly innovative application of in vivo endogenous T cell activation involves the direct programming of CAR-T cells within the body using hydrogel-based delivery. As discussed previously, while CAR T cell therapies have demonstrated exceptional efficacy in certain hematologic malignancies, the general approach is hindered by prolonged manufacturing times and poor infiltration into solid tumors [175,176]. By enabling in situ CAR expression through localized delivery, this hydrogel-based strategy offers a more rapid, scalable, and potentially more effective alternative to conventional ex vivo-engineered CAR T cell therapy. In a recent study, researchers engineered an injectable hydrogel to locally deliver folate receptor α (FRα)-targeting CAR plasmids to endogenous T cells without requiring ex vivo manipulation [177]. In brief, CAR plasmids under the control of a T cell-specific CD2 promoter were complexed with methoxy-PEG-b-poly(ε-caprolactone)-b-poly(ethylene imine) to form nanoparticles, which were further coated with anti-CD3-conjugated polyglutamic acid to enhance T cell-specific uptake. These targeted complexes were then mixed with α-cyclodextrin and Pluronic F-127, to generate a supramolecular hydrogel matrix capable of sustained, localized release of the nanocomplexes. Mice treated with CAR-encoding hydrogels adjacent to solid tumors exhibited significantly suppressed tumor growth and increased proportions of CAR-expressing T cells in the spleen, peripheral blood, and tumor compared to those receiving intravenously infused CAR T cells.

#### 3.1.3. Infiltration via Chemokine Gradients

While activation of endogenous T cells is an inventive approach that bypasses longstanding barriers associated with ex vivo expansion, the overall success of this strategy still depends on the effective recruitment of T cells to the tumor site. Unfortunately, solid tumors often present a hostile immune microenvironment with poor T cell infiltration, limiting the therapeutic impact of otherwise potent immune interventions. To address this challenge, chemokine-based strategies have emerged as a means to modulate T cell trafficking and improve their accumulation within tumors. Chemokines are a family of small signaling proteins that play a crucial role in directing the movement and localization of immune cells throughout the body. In the case of T cells, specific chemokine-receptor interactions orchestrate both their entry into secondary lymphoid organs for initial activation and their subsequent homing to peripheral tissues where they exert effector functions [178]. By recapitulating these natural chemotactic cues, hydrogel-based chemokine delivery platforms aim to recruit and retain functional TILs within tumors, thereby boosting immune-mediated tumor clearance.

Several studies have demonstrated the promise of chemokine-loaded hydrogel systems in reshaping T cell trafficking and enhancing anti-tumor immunity [171,179,180,181,182]. In one approach, an injectable alginate hydrogel functionalized with β-cyclodextrin was loaded with CCL25 to selectively recruit CCR9^+^CD8^+^ T cells, a subset shown to have potent anti-tumor activity [179,183]. To further reinforce anti-tumor responses, this system also incorporated the use of aPD-1 and aPD-L1 antibodies to promote increased interactions between migrating T cells and tumor cells. In B16-F10 melanoma models, CCL25 delivery significantly improved the proportion of infiltrating CD8^+^ and CCR9^+^CD8^+^ T cells on days 3, 6, and 9 following intratumoral hydrogel injection. In a separate study, researchers coated metal–organic nanoparticles with PEG diacrylate (PEGDA) hydrogel to load and sustainably release CCL19, a chemokine that recruits both naive and memory T cells [178,180]. The cores of the nanoparticles were additionally loaded with oxaliplatin for tumor cell killing, while the outer surface was functionalized with aPD-L1 for tumor targeting. Among the tested formulations, those with CCL19 embedded in the PEGDA layer led to enhanced infiltration of both CD4^+^ and CD8^+^ T cells into the TME and reduced T cell sequestration in the spleen compared to oxaliplatin-only or untreated controls.

Together, the results from these studies highlight the potential of chemokine-releasing hydrogels to actively reshape the tumor immune landscape by guiding the recruitment of desirable T cell subsets. Continued refinement of these delivery strategies may provide a powerful means of enhancing endogenous T cell responses and broadening the impact of immunotherapy across a wider range of tumor types.

#### 3.1.4. Exhaustion

Despite their robust effector capabilities, in the context of persistent antigen exposure and chronic inflammation, as commonly occurs in the TME, T cells can become functionally exhausted. This state is characterized by sustained expression of inhibitory receptors (e.g., PD-1, TIM-3, and/or LAG-3), diminished cytokine production, reduced proliferative capacity, and impaired cytolytic function [184]. While exhaustion represents an adaptive mechanism to limit immune-mediated tissue damage, it also severely compromises anti-tumor immunity and is a major obstacle to the success of T cell–based immunotherapies.

To reinvigorate exhausted T cells in the TME, several hydrogel-based platforms have been developed to modulate intrinsic T cell biology and/or extrinsic immunosuppressive signals. For instance, Zhang et al. focused on reducing hypoxia in the TME while simultaneously promoting mitochondrial biogenesis in TILS in order to reverse mitochondrial dysfunction, a key contributor to T cell exhaustion [185]. In their work, the authors designed a multi-component injectable hydrogel that formed in situ at the tumor site by crosslinking oxidized sodium alginate with tumor cell membrane vesicles (O-TMVs). The hydrogel architecture enabled controlled delivery of key components including axitinib, a VEGF receptor inhibitor to reduce hypoxia, embedded within the lipid bilayer of the O-TMVs and free anti-41BB antibody, to promote mitochondrial biogenesis, localized within hydrogel cavities. Hydrogels incorporating both treatments (O-TMV@AB) increased the proportion of cytotoxic and central memory CD8s, while significantly reducing PD-1 expression on T cells compared to saline, O-TMV, and O-TMV@A treatments. Similarly, another group developed a tumor lysate-based hydrogel co-loaded with nicotinamide riboside and GSK-3 inhibitor SB415286 to promote mitophagy in T cells [186]. This formulation achieved a reduction in the accumulation of depolarized mitochondria and mitochondrial ROS, leading to a marked decrease in PD-1 expression from 43.2% to 16.9%, increased proliferation, and increased INF-ɣ secretion compared to a PBS control.

In contrast to strategies that reprogram T cells biologically, one group developed a biomaterial-based approach to modulate T cell-tumor cell interactions through physical separation to prevent exhaustion [187]. Hydrogels composed of PEG and poly(ε-caprolactone), encapsulating gold nanorods and bearing different surface functional groups (-OH, -COOH, or -NH_2_) were used to create a biomimetic physical barrier (BPB) designed to transiently separate T cells from tumor cells, thereby shielding T cells from persistent antigen stimulation and premature exhaustion. The BPB allowed T cells to accumulate and once a sufficient population had built up, near-infrared light irradiation was used to dismantle the barrier enabling timed release of T cells for tumor engagement. This delayed engagement enhanced immediate tumor killing while also promoting systemic immune activation and durable memory responses.

### 3.2. Dendritic Cells

**Figure 4:**
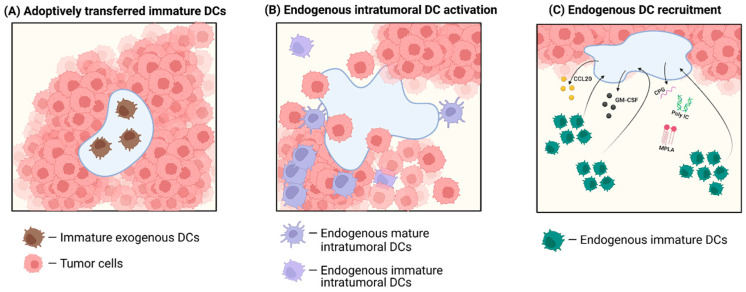
Delivery strategies for DC hydrogel vaccine: (**A**) Hydrogel containing immature exogenous DCs is injected intratumorally. (**B**) Hydrogel is designed to activate and interact with endogenous intratumoral DCs. (**C**) Hydrogel releases DC recruiting signals (CCL20, GM-CSF, CPG, Poly(I:C), or MPLA) to attract endogenous DCs from the surrounding area. Created in BioRender. https://BioRender.com/a35ir4v (accessed on 29 October 2025).

DCs are potent antigen-presenting cells that play a central role in initiating and regulating adaptive immune responses. They are uniquely equipped to acquire antigens in peripheral tissues, process them into peptides, and migrate to secondary lymphoid organs where they present the peptides to T cells. Activation of DCs is often initiated through pattern recognition receptors such as TLRs, which detect conserved molecular patterns from pathogens or damaged cells. Upon TLR engagement, DCs receive potent maturation signals that drive increased surface expression of MHC molecules, upregulation of co-stimulatory molecules such as CD80 and CD86, and secretion of cytokines that guide T cell polarization [188]. While the majority of cell-based immunotherapies have focused on enhancing T cell responses directly, some researchers have turned to manipulating DCs as an upstream strategy for initiating and shaping anti-tumor immunity. Targeting DCs offers the advantage of priming a broader and more diverse T cell response by efficiently presenting a variety of tumor antigens and coordinating the activation of multiple T cell subsets, potentially overcoming some limitations of therapies that act only on mature T cells. In the context of cancer, however, endogenous DCs are often functionally impaired or excluded from the TME, limiting their ability to effectively capture tumor antigens and prime T cells. Additionally, tumors frequently suppress DC maturation and function through immunosuppressive cytokines, metabolic constraints, and regulatory cell populations [188,189]. In light of these barriers, restoring or enhancing DC function using hydrogel-based techniques has emerged as a promising approach to reinvigorate adaptive immune responses and improve therapeutic outcomes in cancer (Figure 4).

#### 3.2.1. Immature DC Vaccines

Adoptive DC therapies, often referred to as DC vaccines, typically involve ex vivo antigen loading of autologous immune cells prior to reinfusion [190]. As of August 2025, the only FDA-approved DC vaccine on the market is Sipuleucel-T which has demonstrated modest clinical benefit in prostate cancer, extending progression-free survival by approximately four months [191]. In general, the broader implementation of DC vaccines has been limited by complex manufacturing logistics, the need for lengthy ex vivo manipulation, and poor trafficking of transferred cells after transfer [190].

Similarly to ACT strategies using naïve T cells, direct delivery of autologous immature DCs that have not been pre-loaded with antigen offers a means to locally increase the number of functional immune cells within or around the tumor, while bypassing the logistical and biological challenges associated with ex vivo manipulation and systemic reinfusion [91,192,193]. Yang et al. implemented this approach by co-loading a peptide nanofibrous hydrogel with immature bone marrow-derived DCs (BMDCs), PD-1 blocking antibodies, and tumor lysate [192]. This method, both with or without PD-1 blockade, was shown to increase the frequency of CD86^+^ and MHCII^+^ DCs in the dLNs compared to antigen-pulsed DCs or hydrogels encapsulating antigen alone over the course of three rounds of vaccination. Additionally, the system enhanced downstream T cell responses, increasing the number of IFN-γ-producing CD8^+^ T cells in the dLNs, spleens, and tumor. Taking a similar yet distinct approach, another group developed a cyclodextrin-PEG hydrogel platform that contained dying tumor cells loaded with nanoadjuvants [193] and immature BMDCs. Following injection, the dying tumor cells released tumor antigens while the nanoadjuvant promoted DC maturation. This strategy resulted in enhanced DC migration to the dLNs and increased the frequency and cytotoxic activity of CD8^+^ and CD4^+^ IFN-γ-producing T cells. Additionally, mice treated with this system exhibited slowed tumor growth and improved survival compared to controls. Notably, both of these studies also demonstrated activation of endogenous DCs, a key feature for sustaining long-term antitumor immunity [192,193].

#### 3.2.2. Tumor-Resident DC Maturation

Although DC-based vaccines have shown promise, adoptive transfer of DCs faces challenges such as poor migration to lymph nodes, limited longevity in vivo, and suboptimal interactions with T cells compared to endogenous lymph-node-resident DCs [194,195,196]. Consequently, several strategies have instead focused on activating DCs already present in the TME to leverage their natural trafficking, localization, and antigen-scavenging capabilities [186,197,198,199]. One such approach employed a PEG-crosslinked melittin–peptide hydrogel (UF@MRP) which not only induced local tumor cell death itself, but also simultaneously delivered encapsulated cytokines, DAMPs, and tumor neoantigens collected from the secretions of irradiated tumor cells [197]. When injected intratumorally and combined with aPD-1 this platform enhanced DC maturation and delayed tumor growth in multiple therapeutic tumor models. Furthermore, combining UF@MRP with systemic aPD-1 treatment more than doubled TIL infiltration with infiltrating T cells exhibiting significantly higher motility compared to those in PBS-treated controls. In another study, Gu et al. designed an in situ-forming hydrogel composed of a fusion protein crosslinked with pectin (TL-pectin Gel) [198]. The fusion protein combined trichosanthin, which promotes DC maturation, and IL-2, which supports T cell activity. After delivery of the hydrogel to a post-surgical tumor site, TL-pectin Gel induced a 6.5-fold increase in DC maturation and reduced tumor growth by more than 80%.

Together, these findings highlight the potential of hydrogel-based systems to locally activate DCs and address several limitations associated with DC vaccines. However, it should be noted that their effectiveness ultimately depends on tumor-resident DCs, which often exhibit impaired antigen processing and presentation—an obstacle that future designs will need to overcome [200].

#### 3.2.3. Endogenous DC Recruitment

In response to issues associated with tumor-resident DCs, other researchers have explored strategies to recruit DCs from outside the TME into the hydrogel, where they can be activated and driven toward full maturation [19,193,201,202,203,204,205,206]. Once recruited, these DCs can encounter tumor-associated antigens and receive activation signals within the same environment, enabling a coordinated process of antigen uptake, maturation, and migration to dLNs for T cell priming. The most common approach to promoting DC recruitment is the incorporation of cytokines to establish a local signaling environment that draws DCs toward the material. Several different cytokines have been explored for this purpose, including CCL20, CCL21, FMS-like tyrosine kinase 3 ligand (FLT3L), and granulocyte–macrophage colony-stimulating factor (GM-CSF) [204,206,207,208].

Chemokine CCL20 acts by binding to its receptor, CCR6, on immature DCs, thereby initiating cellular chemotactic migration along the established gradient [209]. One group was able to leverage CCL20’s recruitment ability by incorporating it into an in situ crosslinking hydrogel composed of PEG and dextran [204,207]. This system effectively recruited immature DCs, which were simultaneously exposed to pathogen-mimicking microparticles delivering IL-10 siRNA, pDNA, and the TLR agonist CpG oligodeoxynucleotide (CpG-ODN). In this formulation, the IL-10 siRNA suppressed production of the immunosuppressive cytokine known to hinder DC maturation, the pDNA provided tumor-specific antigen, and the CpG-ODN acted to stimulate DC activation. This multi-pronged approach enabled coordinated recruitment, activation, and maturation of DCs within a single scaffold. Through a series of in vitro assays, the researchers found that this particular combination of signals increased CD86 and CD80 expression, enhanced coexpression of CD86 and MHC II, and elevated secretion of pro-inflammatory cytokines INF-γ, IL-12p70, and TNF-α. Furthermore, in a prophylactic B cell lymphoma model, the combination treatment was able to improve median survival by ≥10 days compared to all other groups underscoring the therapeutic potential of this approach. However, it is important to note that chemokines can also exert pro-tumor effects, such as promoting angiogenesis or recruiting immunosuppressive cell populations [209]. CCL20 specifically has been implicated in driving cancer progression in HCC and colorectal cancer, emphasizing the need for careful selection, dosing, and temporal control when incorporating chemokines into therapeutic designs [210,211].

Another frequently employed strategy is the use of GM-CSF to bolster DC recruitment. While GM-CSF is best known for its role in DC differentiation and maturation in vitro, it has also been used in vivo to attract DCs from the surrounding tissue into hydrogel depots [19,202,205,206,212,213,214]. Several studies have leveraged GM-CSF within hydrogel platforms to recruit and activate DCs for cancer vaccination, with some systems showing double or triple the amount of infiltrating DCs in the hydrogel after only four days, highlighting its tremendous recruiting ability [19,202]. In one study, researchers varied the GM-CSF concentration within the hydrogel and revealed a dose-dependent effect on total cell infiltration, with higher doses attracting more cells overall. However, DC recruitment plateaued between 1.0 and 1.5 µg, suggesting that higher doses may primarily increase the influx of other inflammatory cell types rather than DCs [205]. One particularly notable example of integrating GM-CSF with antigen delivery is the alginate hydrogel-based mRNA cancer vaccine developed by Zhou et al. This alginate-based system was engineered with porogen beads that upon degradation left behind macropores in the alginate to facilitate DC homing [212]. The gel was also loaded with mRNA lipoplexes encoding tumor antigens for DC uptake. Upon subcutaneous injection, the macroporous architecture allowed efficient cell entry, with significantly higher DC numbers in the hydrogel within several days post-implantation. Additional analysis further found a significantly higher number of hydrogel-recruited DCs in the dLNs compared to non-dLNs, as measured by eGFR expression induced by the mRNA. In two prophylactic murine tumor models (E.G7-OVA and 4T1), treatment with the hydrogel vaccine elicited robust antigen-specific CD8^+^ T cell responses and conferred significant tumor growth inhibition compared to all other controls. An additional tumor treatment model also saw delayed tumor growth and improved survival, albeit with less pronounced effects.

The incorporation of GM-CSF into an mRNA vaccine platform represents a particularly innovative approach, as it simultaneously amplifies DC recruitment and ensures potent in situ antigen presentation. Notably, researchers have found that the majority of lipid nanoparticles used for mRNA vaccines remain localized at the injection site and are not efficiently transported to lymph nodes via DCs, pointing to opportunities for strategies that enhance DC targeting and uptake [215,216]. By combining sustained GM-CSF release from hydrogels with mRNA delivery, this approach not only holds promise to improve cancer immunotherapy but could also enhance the efficacy of mRNA vaccines more broadly, including those for infectious diseases.

Despite extensive research, some questions still remain regarding which signaling factors influence DC recruitment. A few researchers, drawing on results from their hydrogel-based DC vaccines, have proposed that tumor lysate alone may serve as a chemotactic signal; these findings, however, may be confounded by the concurrent use of adoptively transferred DCs in their systems, which could release additional factors that attract endogenous DCs [192,193]. Other researchers have also explored the use of TLR agonists, molecules that bind TLRs to initiate intracellular activation signaling on DCs, as potential tools for recruitment, though reported outcomes have been inconsistent. For example, incorporation of the TLR3 agonist polyinosinic/polycytidylic acid [poly(I:C)] into an antigen-loaded polypeptide hydrogel enhanced DC migration in an in vitro transwell migration assay, suggesting a chemotactic effect under controlled conditions [201]. In separate work, Ali et al. was able to support this finding when they investigated the in vivo effects of the TLR agonists CpG, poly(I:C), or monophosphoryl lipid A alongside GM-CSF within a polymeric scaffold system and showed that they significantly increased DC accumulation at the in vivo implantation site more than GM-CSF alone [217]. However, these results are challenged by Bhatta et al. who found that hydrogels incorporating tumor-derived extracellular vesicles and CpG did not increase DC recruitment compared to the CpG-free control in their in vivo system [203].

Collectively, these findings indicate that signaling factors beyond just cytokines and GM-CSF are likely to promote DC recruitment, but their effects are not consistent across delivery platforms or experimental settings. A clearer understanding of how variables such as dosage and release kinetics influence DC trafficking will be essential for designing formulations that use these components to reliably enhance endogenous DC recruitment and activation.

#### 3.2.4. Promotion of Cross-Presentation

Cross-presentation by DCs is crucial for initiating CD8^+^ T cell responses against extracellular antigens, which are normally not presented on MHC class I molecules. Typically, MHC class I displays peptides derived from intracellular proteins to activate CD8^+^ CTLs, whereas MHC class II presents extracellular antigens to CD4^+^ helper T cells. Through the process of cross-presentation, however, APCs can process extracellular antigens for display on MHC class I, thereby enabling activation of CD8^+^ T cells with non-intracellular components [218,219]. Given its central role in generating potent cytotoxic T cell responses, enhancing cross-presentation of antigens is a key objective in cancer DC vaccine development.

As noted previously, TLR agonists play an important role in activating and, in some cases, recruiting DCs, and have therefore been incorporated into a variety of hydrogel-based DC vaccine platforms [91,193,201,203,217,220]. Among them, the TLR9 agonist, CpG-ODN, has been studied extensively for use in cancer treatments. This short piece of single-stranded synthetic DNA has been shown not only to induce DC maturation and proinflammatory cytokine secretion, but also has the ability to enhance cross-presentation [221,222]. With this in mind, researchers that have incorporated CpG-ODN into hydrogel-based platforms have been able to further improve anti-tumor responses.

For example, Liang et al. designed a spontaneous, multifunctional hydrogel vaccine, termed Ncom Gel, that co-assembles CpG-modified carboxymethyl chitosan with partially oxidized mannan [223]. In a murine model, Ncom Gel markedly increased the percentage of CD80^+^ and CD86^+^ DCs in TDLNs, triggering an expansion of IFN-γ–producing CD8^+^ and CD4^+^ T cells and leading to a significant delay in tumor growth. Further evaluation revealed that the hydrogel also elevated the proportion of CD103^+^ CD8α^+^ DCs in TDLNs, a subset known for superior cross-priming of CD8^+^ T cell responses [224,225]. The observed increase suggests that Ncom Gel not only enhances DC activation, but may also enrich the most competent antigen-presenting subsets. Similarly, Yang et al. employed a biodegradable thermosensitive poly(d, l-lactide)-PEG-poly(d, l-lactide) hydrogel to co-deliver CpG, GM-CSF, and tumor lysate [214]. This strategy was shown to significantly enhance the number of CD11c^+^ DCs and to elevate the percentage of CD11c^+^CD86^+^ and CD11c^+^MHCI^+^ DCs in the inguinal LNs of both healthy and tumor-bearing mice three days following vaccination, indicating enhanced maturation and an increased capacity for cross-presentation of extracellular antigens to CD8^+^ T cells.

DCs consist of several subsets with specialized functions, including cDCs (comprising cDC1 and cDC2), pDCs, monocyte-derived DCs, and Langerhans cells [226]. Each subset exhibits distinct capabilities and marker expression that dictate their roles in immune activation. Among these, cDCs excel at priming T cells, with cDC1 being particularly effective at cross-presenting antigens to CD8^+^ T cells. Researchers have shown that the cytokine FLT3L, normally used to support the development and functional maintenance of DCs, also has the ability to recruit cDC1s into the TME, thus expanding overall cross-presentation capacity. Building on this principle, Gao et al. developed an injectable hydrogel composed of Pluronic F-127 and F68 polymers to encapsulate and locally release photosensitizer Ce6, FLT3L, poly(I:C), and STAT3 inhibitor Napabucasin (Nap) [208]. In this system, Ce6 was used in conjunction with photodynamic therapy (PDT) to induce tumoral antigen release, FLT3L promoted the recruitment and expansion of cDC1s, poly(I:C) served to activate DCs, and Nap facilitated CTL-mediated tumor killing. In vitro functional evaluation revealed that BMDCs matured in the presence of FLT3L expressed high levels of CD103, Clec9, and MHC II, classical markers of the cDC1 subset. Moreover, when FLT3L-induced BMDCs were co-cultured with Nap-Ce6–pretreated CT26 cells, the proportion of MHCI^+^ DCs doubled, indicating enhanced cross-presentation potential and suggesting that this hydrogel platform effectively augments the capacity of cDC1s to prime CD8^+^ T cells. Supporting this, intratumoral injection of the hydrogel platform in a B16-F10 murine tumor model significantly suppressed tumor growth and generated higher levels of CD8^+^ TILs compared to hydrogels loaded with GM-CSF instead of FLT3L.

By actively promoting cross-presentation, these approaches provide a powerful framework for the development of next-generation cancer vaccines that more effectively harness cytotoxic T cell-mediated immunity.

#### 3.2.5. Reversing Tolerogenicity

Although methods for promoting tolerogenic DCs have been extensively studied in the context of immune suppression for autoimmunity and allogeneic transplantation, intentionally reversing their phenotype to bolster antitumor immunity has received far less attention [227,228]. In a novel study, Fu et al. developed a programmable, ROS-responsive hydrogel platform (PIVOT) designed to reprogram tolerogenic DCs toward an immunostimulatory state within the TME [229]. To achieve these effects, PIVOT was injected peritumorally after being loaded with three components: (1) oxaliplatin, to induce ICD for antigen generation; (2) Flt-3L, to recruit and expand DC populations locally; and (3) anti-TIM-3, an antibody that blocks the TIM-3 immune checkpoint on DCs, a pathway that normally contributes to an immunosuppressive, tolerogenic state. By inhibiting TIM-3 signaling, anti-TIM-3 shifted DCs toward an activated phenotype, increasing their uptake of tumor cell DNA and promoting secretion of Th1-attracting chemokine CXCL9. Altogether, treatment with PIVOT led to a robust anti-tumor response, as evidenced by enhanced functional CD8^+^ TIL activity, increased numbers of CD11C^+^MHCII^+^ DCs at the tumor site, and improved tumor regression in a murine B16-F10 model. These findings demonstrate the potential for hydrogel-based strategies to not only recruit and activate DCs, but also to overcome the immunosuppressive conditions that limit their function in cancer.

### 3.3. Macrophages

Macrophages are innate immune cells that serve as highly adaptable regulators of immunity, capable of both initiating and resolving immune responses [230]. Upon encountering diverse signals in their environment (i.e., pathogen-associated molecular patterns (PAMPs), DAMPs, cytokines, and growth factors), macrophages undergo functional activation that tunes their phenotype and effector roles. Broadly, this spectrum of activation is often simplified into two categories: pro-inflammatory M1 macrophages, which phagocytose pathogens, secrete inflammatory mediators (e.g., TNF-α, IL-12, and reactive oxygen/nitrogen species), and present antigens to T cells via MHC molecules, and anti-inflammatory M2 macrophages, which produce cytokines such as IL-10 and TGF-β and release growth factors that promote tissue repair, angiogenesis, and remodeling. This remarkable plasticity allows macrophages to maintain tissue homeostasis, respond to acute challenges, and shape downstream immune outcomes. However, the same flexibility that underlies their beneficial roles also makes them highly susceptible to dysregulation in pathological contexts. In particular, the TME provides a complex network of signals that can profoundly alter macrophage function, often in ways that suppress immunity and facilitate tumor progression [231].

#### 3.3.1. Adoptively Transferred Macrophages

Compared to T cells and DCs, adoptive transfer of macrophages has received far less attention in recent decades, with studies mainly focusing on disease contexts other than cancer or specifically on the use of CAR-M therapies [232,233,234,235]. Previously, a number of researchers attempted human clinical trials using adoptively transferred macrophages to treat cancer in the 1990s and early 2000s, but had varying degrees of success [236,237,238,239]. Building on this earlier work, and motivated by positive outcomes observed with their own direct injections of pro-inflammatory macrophages, Guerra et al. developed a thiolated gelatin and PEGDA crosslinked hydrogel to facilitate the adoptive transfer of M1-polarized macrophages [240]. In a murine HCC model, hydrogel-delivered M1 macrophages markedly inhibited tumor growth compared to either unloaded hydrogel or untreated controls. Mechanistic analyses revealed increased secretion of pro-inflammatory cytokines such as TNF-α (9.5-fold increase) and IL-6 (5.8-fold increase) by hydrogel-delivered M1 cells relative to untreated controls, along with significantly elevated caspase-3 activity within tumor tissues, indicative of enhanced apoptotic cell death. Collectively, these findings suggest that hydrogel-based delivery not only improves the functional output of adoptively transferred macrophages, but also amplifies their ability to trigger tumor cell apoptosis, offering a promising strategy to revitalize macrophage therapies.

#### 3.3.2. Manipulation of Tumor-Associated M1 Macrophages

Given their ability to drive pro-inflammatory responses and directly mediate tumor cell killing, M1 macrophages represent an appealing therapeutic target within the TME. To this end, Jiang et al. developed a PEG-PLGA-PEG triblock copolymer hydrogel designed to harness the effector functions of M1 TAMs [241]. The hydrogel was loaded with L-arginine-rich nanoparticles (G4-Arg) that encapsulated the chemotherapeutic drug DOX (G4-Arg/DOX). This system sought to leverage the high iNOS activity of M1 macrophages enabling conversion of L-arginine into nitric oxide (NO), a potent cytotoxic mediator that promotes tumor cell apoptosis. To evaluate nanoparticle effectiveness, RAW 264.7 macrophages were co-cultured in vitro with G4-Arg or control non-L-arginine-based nanoparticles, and intracellular NO levels were quantified. Macrophages treated with G4-Arg or G4-Arg/DOX nanoparticles displayed significantly elevated NO production compared to all other groups, and functional assays demonstrated 79% tumor cell death in a transwell co-culture model with G4-Arg/DOX. The in vivo efficacy was then assessed in a 4T1 murine breast cancer model by subcutaneously injecting gel-encapsulated nanoparticles near the tumor and monitoring tumor growth over 24 days. The Gel/G4-Arg/DOX formulation achieved the greatest tumor suppression relative to all controls, including gel alone, Gel/G4-Lys, and Gel/DOX. Remarkably, a single administration of Gel/G4-Arg/DOX outperformed four systemic doses of intravenously delivered DOX. Immunohistochemical analysis further confirmed that Gel/G4-Arg/DOX treatment was associated with reduced expression of the M2 macrophage marker CD163 and increased expression of the M1 marker CD11c, indicating a favorable shift in the ratio of anti-tumor to pro-tumor macrophage phenotypes. All in all, these findings highlight the potential of hydrogel-based delivery systems to utilize M1-specific effector functions to enhance therapeutic efficacy of macrophage-based treatments for solid tumors.

#### 3.3.3. Converting M2 to M1

While strategies to boost M1 macrophage function have shown promise, the majority of TAMs in solid tumors are skewed toward an immunosuppressive M2-like phenotype that fosters tumor growth and dampens anti-tumor immunity. Consequently, a complementary line of research has focused on reprogramming these M2-like TAMs into an M1-like state using a wide range of strategies. For example, Yang et al. engineered a PLL-PEG hydrogel to co-deliver polyphyllin (PP2), a steroidal saponin previously found to modulate macrophage phenotype, and resiquimod (R848), a TLR agonist [242,243,244]. To test the effectiveness of the hydrogel system, termed PR-Gel, M2-skewed RAW 264.7 cells were treated with PR-Gel for 48 h in vitro and subsequently stained for phenotypic markers to evaluate the M1/M2 ratio. PR-Gel was shown to significantly increase the expression of iNOS and significantly decrease the expression of M2 marker CD206 compared to cells treated with single drugs or free PP2 + R848 indicating a dramatic shift towards an M1 phenotype in the population. Moreover, analysis of cytokine secretion found overall higher levels of pro-inflammatory cytokines TNF-α and IL-6 than immunosuppressive cytokine IL-10. When tested in vivo, mice bearing MFC gastric tumors were treated peritumorally with PR-Gel or drug/hydrogel controls. The TME from PR-Gel–treated mice displayed the same expression trends observed in vitro, with elevated iNOS and TNF-α and reduced CD206 and IL-10, confirming effective macrophage reprogramming within the TME. PR-Gel-treated mice also exhibited the strongest tumor growth inhibition and significantly prolonged survival compared to all control groups.

Another innovative approach was reported by Dai et al., who designed a hybrid peptide melittin-(RADA)6 hydrogel, which not only exhibited direct tumor killing, but also served as a depot for cargo targeting Ca^2+^/calmodulin-dependent protein kinase II (CaMKII), a signaling molecule implicated in modulation of macrophage functionality [245]. In vitro studies of the CAMKII inhibitor KN93 drug-loaded hydrogel (MRK) demonstrated that TAMs exposed to the system upregulated transcription of M1-related markers such as CD86, CD40, and Nos2, while reducing transcription of M2-related markers like CCL5, Vegfa, and Tgfb3 in a concentration-dependent manner. Follow-up in vivo testing in a B16-F10 mouse melanoma model confirmed these findings, showing a robust tumor inhibition rate of 79% after 13 days. Additionally, the percentage of M2 macrophages in the TME was significantly reduced compared to mice treated with only hydrogel or KN93, highlighting the synergistic effects of the treatment. Interestingly, while not directly investigated by the authors, the use of melittin as a hydrogel backbone may have also contributed to M2-to-M1 reprogramming, as reported in other studies [197,246,247].

Using a more complex strategy, Li et al. engineered a hydrogel system incorporating three distinct macrophage-modulatory components [248]. First, the hydrogel matrix itself was derived from artificial exosomes generated by genetically engineered M1-type macrophages and chemically coated with sodium alginate oxide to form a gelator. As a result of the extrusion process used to fabricate artificial exosomes, the exosomes carried an increased load of pro-inflammatory proteins and RNAs, equipping them to reprogram M2 macrophages into an M1 state. Next, to further enhance macrophage activity, the engineered macrophages were modified to overexpress SM1Aexo, which competitively blocks CD24 on the surface of tumor cells, thereby promoting greater phagocytosis by TAMs. Finally, the hydrogel was loaded with MRX-2843, a small molecule inhibitor of efferocytosis—a process that typically suppresses inflammatory responses and limits cross-presentation by macrophages. In vitro studies confirmed the macrophage-reprogramming capacity of this system, showing that exosome-treated macrophages upregulated the M1 marker CD86 and downregulated the M2 marker CD206. Additional assays demonstrated that inclusion of SM1Aexo and MRX-2843 further enhanced anti-tumor activity, as evidenced by increased tumor cell phagocytosis and effective blockade of CD24. Extending these findings in vivo, the hydrogel system was tested in an advanced ovarian cancer mouse model. Intraperitoneal injection of the exosome-hydrogel followed by a single dose of X-ray radiation significantly increased the percentage of M1 macrophages in the TME, consistent with successful macrophage reprogramming, and led to marked suppression of tumor growth compared to controls.

Collectively, these studies underscore the versatility of hydrogel platforms for TAM repolarization. By incorporating diverse modalities, hydrogels can simultaneously disrupt M2-polarizing cues and reinforce pro-inflammatory signaling. However, while hydrogel-based strategies to convert M2-like TAMs into M1-like phenotypes have demonstrated encouraging preclinical outcomes, it is important to recognize that the traditional M1/M2 framework oversimplifies macrophage biology. In reality, TAMs exist along a continuum of activation states, often co-expressing features of both classical and alternative polarization [249]. Recent work has highlighted additional macrophage subtypes with distinct transcriptional and functional profiles, suggesting that more nuanced classifications will be critical for guiding therapeutic design [231,250,251]. Moving forward, refining our understanding of macrophage heterogeneity in the TME will be essential to fully harness their therapeutic potential in cancer.

## 4. Reshaping the Immune Environment

While direct activation of immune effector cells can enhance antitumor responses, the broader TME often dictates whether these cells can sustain their function once inside tumors. The TME is composed of stromal elements, vasculature, ECM, and a diverse array of immune cells, many of which are co-opted to support tumor growth and dampen anti-tumor immunity. Factors such as hypoxia, aberrant vasculature, dense ECM, and the accumulation of suppressive cell populations collectively create physical and biochemical barriers that blunt immune activity [252]. Hydrogel-based systems have therefore been developed not only to modulate cells, but also as tools to re-engineer the TME itself. In this section, we review hydrogel-based strategies aimed at reshaping hostile tumor niches including the delivery of immunoregulatory molecules (Section 4.1), TME remodeling (Section 4.2), and induction of tertiary lymphoid-like structures (Section 4.3). Together, these approaches highlight how hydrogels can transform suppressive tumor environments into sites more conducive to immune infiltration, persistence, and function.

### 4.1. Delivery of Immunoregulatory Molecules

#### 4.1.1. Immune Checkpoint Blockade

Due to the potent effector functions of activated T cells, tumors often develop strategies to subvert or resist T cell-mediated immunity. Such resistance can arise through multiple methods including upregulation of immune checkpoint ligands, downregulation of antigen presentation, or remodeling of the TME to suppress T cell infiltration and function. To counter these immune escape mechanisms, many conventional immunotherapies have focused on reactivating or augmenting T cell responses. Immune checkpoint blockade (ICB) exemplifies this approach by employing antibodies to target inhibitory receptors such as PD-1 and CTLA-4 on T cells and PD-L1 on tumors, thereby preventing tumor-driven suppression of T cell activity. Unfortunately, while these therapies have shown remarkable clinical success in certain cancers, particularly melanoma and lung cancer, their efficacy remains limited with variable response rates, and their systemic distribution can cause off-target effects or systemic immune activation leading to severe toxicity [2,253].

To mitigate challenges associated with ICB, researchers have developed hydrogel-based delivery systems that localize ICB agents within or near tumors, thereby enhancing therapeutic efficacy while minimizing systemic exposure [19,96,254,255]. For example, Kim et al. demonstrated that local ipsilateral injection of a F127/PEG hydrogel loaded with aPD-1 and anti-CTLA-4 significantly increased antibody retention at the injection site and reduced both tumor burden and liver toxicity compared to bolus delivery in a murine breast cancer model [256]. Building on this approach, combinatorial strategies using ICB in conjunction with other immunomodulators have also been employed to improve the synergistic effects of multi-agent immune treatments [113,257,258,259,260]. One group utilized an alginate-based hydrogel to support the sustained release of aPD-1 alongside various ICD–inducing chemotherapeutics. Ultimately, they found that intratumoral injection of alginate hydrogel loaded with oxaliplatin, R837, and aPD-L1 not only eradicated the primary tumor, but also elicited a robust abscopal effect on distant tumors and protected against tumor rechallenge. In contrast, the same drug combination delivered intratumorally without the hydrogel led to greater tumor progression of both primary and secondary tumors, revealing a diminished effectiveness without the sustained and localized release provided by the hydrogel matrix [113].

Other platforms have further leveraged bioresponsive hydrogels to synchronize ICB antibody release with tumor-associated microenvironmental conditions. One such formulation, composed of a spontaneously assembling in situ hydrogel (P-NT) delivered via intratumoral injection, was designed to respond to MMPs in the TME to control the release of aPD-1 (P-NT-aPD1) [261]. This system was shown to reduce the systemic dissemination of aPD-1 observed after free antibody injection while simultaneously prolonging its intratumoral retention to at least 7 days. Treatment with P-NT-aPD1 also increased the proportion of tumor-infiltrating CD4^+^ and CD8^+^ T cells and decreased the percentage of PD-L1-expressing non-immune cells after 25 days. Functionally, the platform, which additionally included camptothecin treatment, achieved 100% tumor regression in two separate mouse models.

Overall, hydrogel-based encapsulation approaches represent a promising direction for enhancing the precision, potency, and safety of ICB therapies by enabling sustained, localized, and co-modulated delivery within immunologically relevant compartments.

#### 4.1.2. Diversion of Metastatic Tumor Cells

Metastasis, the spread of tumor cells from a primary site to distant organs, is the leading cause of cancer-related mortality. This multistep process involves local invasion, intravasation into the bloodstream or lymphatics, survival during circulation, extravasation into secondary tissues, and colonization to form metastatic lesions. Each step is tightly regulated by both intrinsic tumor cell properties and extrinsic signals from the microenvironment [262]. Chemokines, while most often associated with immune cell chemotaxis, have also been implicated in guiding tumor cell migration and contributing to the establishment of pre-metastatic niches [209,263]. Consequently, intercepting CTCs by exploiting chemokine gradients represents a promising strategy to limit metastatic progression. With this in mind, researchers have developed hydrogel-based platforms that act as localized “traps,” limiting systemic tumor spread while providing opportunities for therapeutic intervention.

One example of this approach is the modular hydrogel developed by Ji et al., designed to function both as a physical sink for CTCs and as an immune training hub [206]. The system combined GelMA with nanoclay, tumor cell-derived exosomes containing GM-CSF mRNA, the sonosensitizer Ce6, and encapsulated CCL21a. Cargo release was programmed in stages, with CCL21a released first to establish a chemokine gradient that diverted CTCs away from TDLNs and toward the hydrogel. In a murine model, treatment with this system revealed a marked reduction in lung metastases relative to controls, a finding further corroborated by flow cytometry analysis, showing the lowest numbers of CTCs in peripheral blood. Additionally, by capturing disseminating tumor cells in situ, the hydrogel was able to effectively convert them into a continuous source of tumor antigen for presentation to DCs recruited by GM-CSF. Collectively, the system produced the strongest therapeutic outcomes, as treated mice exhibited prolonged survival, with half remaining tumor-free at day 54.

Along the same lines, other researchers have specifically focused on exploiting the CXCL12–CXCR4 axis, another driver of tumor cell migration and metastatic colonization [209]. In one study, Ierano et al. utilized an HA-based hydrogel loaded with CXCL12 (CLG) to act as a trap for circulating melanoma cells expressing CXCR4 [264]. Subcutaneous injection of CLG in a syngeneic model of melanoma lung metastasis resulted in significantly greater tumor cell recruitment compared to empty gels (120 ± 36.7 vs. 16 ± 6.3 respectively) and significantly fewer metastatic lung sites (2.5 vs. 5.9 lesions per section). Similarly, Chen et al. developed a “bait-and-hook” hydrogel (BH-gel) using PVA and borax that coupled sustained CXCL12 release with embedded DOX, enabling simultaneous sequestration of CTCs and localized tumor cell killing [265]. In a metastatic xenograft model of lung carcinoma, BH-gel treatment was able to significantly reduce both overall tumor burden and the number of metastatic nodules in the mesentery compared to free DOX or saline controls.

Altogether, these promising results suggest that hydrogel-based interception of CTCs represents a powerful and underexplored approach, warranting further investigation to fully realize its potential in limiting metastasis and improving patient outcomes.

### 4.2. Tumor Microenvironment Remodeling

Under normal physiological conditions, tissue homeostasis is preserved through balanced regulation of factors such as pH, oxygen tension, metabolic activity, inflammatory signaling, and intercellular communication [266,267]. Tumors, however, hijack these same processes to establish an immunosuppressive milieu—driving acidosis, inducing hypoxia, promoting chronic inflammation, and rewiring local signaling networks to impair effective immune surveillance [268]. These maladaptive conditions not only sustain tumor growth, but also limit the success of immunotherapies that rely on robust immune activation [269]. Hydrogel platforms, with their capacity for localized delivery, dynamic responsiveness, and structural versatility, offer a promising means to reprogram these features within the TME, creating conditions that support robust and sustained anti-tumor immunity.

#### 4.2.1. pH

A defining feature of the TME is its acidic extracellular pH, which arises from aberrant metabolic activity, high glycolytic flux, and inadequate vascular perfusion. This acidification exerts profound immunosuppressive effects, impairing T cell proliferation and cytotoxicity, limiting DC activation, and skewing macrophages toward tumor-promoting phenotypes. Consequently, therapeutic approaches that neutralize acidity hold considerable promise for restoring immune activity [270].

Towards this end, Huo et al. designed a biomineralized hydrogel vaccine aimed at reversing acidity-driven immune suppression while simultaneously amplifying DC activation [199]. The hydrogel was generated by fusing fixed membrane proteins from 4T1 cells with DCs (FP), mixing them with CaCl_2_ to form a silk fibroin-based matrix (SH), and subsequently soaking the scaffold in Na_2_CO_3_ to yield the final SH@FP@CaCO_3_ hydrogel. In this multi-component construct, the researchers aimed not only to simultaneously deliver tumor antigens and costimulatory molecules to promote activation of T cells, but also most notably, CaCO_3_ particles, which served to neutralize the acidic TME. Under acidic conditions, macrophages are preferentially polarized toward an M2-like, tumor-supportive phenotype; by buffering local pH, the released CaCO_3_ particles aimed to counteract this effect, driving repolarization toward an M1 state and thereby relieving one of the major suppressive barriers to antitumor immunity [271]. Flow cytometry confirmed this effect in vitro, showing that the addition of CaCO3 to the media of M2-polarized macrophages induced their repolarization and increased secretion of IL-12 and TNF-α by 1.7- and 2.8-fold, respectively, compared to untreated controls. In vivo, treatment with SH@FP@CaCO_3_ in a 4T1 tumor model led to a TME enriched with 150% more M1 macrophages and 75% fewer M2 macrophages relative to controls, demonstrating effective reprogramming of macrophage phenotypes.

In a similar approach, Liang et al. sought to reprogram the acidic TME using a multi-component hydrogel system also incorporating CaCO_3_ [272]. Specifically, the group developed an in situ-forming Pluronic F-127/HA hydrogel (TCCaGM) co-loaded with CaCO_3_ nanoparticles, tunicamycin (Tuni; an endoplasmic reticulum stress inducer), GM-CSF, and catalase (CAT). In this formulation, CaCO_3_ served to buffer the acidic milieu, once again mitigating macrophage polarization toward an M2 phenotype and supporting repolarization toward an antitumor M1 state. To further prime the immune response, CAT alleviated hypoxia by decomposing H_2_O_2_, and Tuni enhanced tumor immunogenicity by inducing ER stress to upregulate the expression of “eat me” signal calreticulin (CRT) on tumor cells. The combined effect was a remodeled TME more permissive to immune activation, characterized by increased intratumoral M1 macrophages, reduced M2 prevalence, elevated CRT expression, and decreased Ki67 (a marker of proliferation). In vivo, treatment with TCCaGM in a 4T1 tumor model resulted in the most pronounced delay in tumor growth and the greatest survival benefit compared to control formulations.

Together, these studies highlight the capacity of pH-responsive hydrogels to directly remodel acidic conditions that suppress anti-tumor immunity. Although both approaches primarily targeted macrophage polarization, future efforts could extend this strategy to other immune populations influenced by acidity, including T cells, NK cells, and myeloid-derived suppressor cells.

#### 4.2.2. Hypoxia

Hypoxia represents another hallmark of the TME that drives immune suppression and therapeutic resistance. Oxygen deprivation not only stabilizes hypoxia-inducible factors (HIFs) that promote tumor progression, but also directly impairs the effector functions of T cells and NK cells, while favoring the recruitment of immunosuppressive cell types such as TAMs and MDSCs [273].

To counter these effects, several groups have leveraged CAT-based strategies to locally generate oxygen and relieve hypoxia. In one example, Zhang et al. developed an in situ injectable chitosan/dextran hydrogel co-loaded with CAT and HA-based transferomes carrying chlorogenic acid (CHA) [274]. In vitro, CAT catalyzed the decomposition of endogenous H_2_O_2_ into O_2_ in an H_2_O_2_ concentration–dependent manner, as evidenced by bubble formation assays. Hydrogel treatment also successfully alleviated hypoxia within the TME when employed in vivo, as reflected by downregulation of hypoxia marker HO-1. Beyond oxygen restoration, inclusion of CHA in the system further promoted TAM repolarization toward an M1 phenotype, thereby enhancing local antitumor immunity. Mice treated with the hydrogel exhibited not only reduced hypoxic burden but also increased IFN-γ and TNF-α secretion from macrophages in the blood, spleen, and TME, culminating in significantly suppressed tumor growth. As previously mentioned, Liang et al. also incorporated CAT into their multi-component TCCaGM hydrogel in order to relieve hypoxia [272]. Notably, CAT retained high enzymatic activity for at least 6 h when encapsulated in the TCCaGM, whereas free CAT lost most of its activity within 3 h, indicating that the hydrogel both preserved and protected enzymatic function. In vivo, treatment with TCCaGM significantly reduced HIF-1α levels, further validating its capacity to alleviate hypoxia in the TME.

Building on the strategy of leveraging CAT activity to mitigate tumor hypoxia, researchers have also explored the use of inorganic nanozymes that mimic CATfunction. Zhang et al. focused on this strategy when they developed an oxygen-generating F127/F68 hydrogel system incorporating HA- and L-arginine-modified cerium oxide nanozymes (HCePAs) loaded with photosensitizers [275]. Within the TME, HCePAs were able to catalyze the reaction of introduced CaO_2_ with water to produce O_2_, thereby relieving local hypoxia. Remarkably, intratumoral injection of the hydrogel increased oxygen levels 3.3-fold across the entire tumor area within only 4 h, and elevated O_2_ concentrations were sustained for 3 days and remained higher than controls out to at least 7 days. To evaluate its therapeutic potential, the authors combined the hydrogel with PDT, which is otherwise strongly limited by hypoxic tumor conditions [276]. The system enabled localized and sustained release of photosensitizers while generating NO and O_2_, leading to significant inhibition of tumor growth. Notably, repeated irradiation produced the strongest effects, with the smallest tumor volumes, highest levels of apoptosis, and largest evidence of necrosis, all achieved without systemic toxicity.

In contrast to oxygen-supplementing strategies, other groups have instead sought to alleviate hypoxia by reducing oxygen consumption within the TME. Chao et al. demonstrated this approach by engineering a lyophilized alginate hydrogel scaffold capable of co-delivering metformin, a mitochondrial complex I inhibitor known to suppress tumor cell respiration, and CAR-T cells (CAR-T@Met/gel) directly into postsurgical tumor cavities [277]. When combined with CAR-T therapy, this intervention also improved CAR-T persistence and effector function, resulting in markedly enhanced tumor control compared with CAR-T treatment alone. Metformin treatment was shown to inhibit glycolysis and reduce oxygen consumption rates in tumor cells by approximately 33% relative to untreated cells, significantly relieving hypoxia. CAR-T cells on the other hand exhibited increased basal oxidative phosphorylation and maximal respiration capacity after metformin exposure, indicating enhanced metabolic fitness. Functionally, this dual approach translated into superior local tumor control, with CAR-T@Met/gel outperforming both i.v. CAR-T plus metformin and CAR-T-loaded hydrogel without metformin.

#### 4.2.3. Inflammation

Chronic inflammation is a hallmark of many tumors and plays a complex role in shaping the TME. While acute inflammatory signals can recruit and activate immune cells, persistent inflammatory cues often promote immunosuppression, tumor progression, and therapeutic resistance. In particular, sustained inflammatory signaling can drive the accumulation of immunosuppressive Tregs and MDSCs, which dampen antitumor immune responses [278]. Accordingly, strategies that selectively modulate inflammation within the TME have the potential to restore immune function and enhance the efficacy of immunotherapies.

Towards this end, Chen et al. developed an injectable anti-inflammatory nanofiber hydrogel (BetP-Gel) designed to reprogram the TME while enhancing systemic immune responses [279]. The hydrogel served a dual purpose: it functioned both as a local depot for sustained release of aPD-L1 and simultaneously delivered steroidal anti-inflammatory agents. The hydrogel was constructed from betamethasone phosphate (BetP), a widely used anti-inflammatory drug, which can self-assemble into nanofibers via non-covalent interactions. Remarkably, the researchers found that BetP-Gel alone was able to modulate multiple inflammatory factors. Upon in vitro assessment, it was shown to reduce the production of pro-inflammatory cytokines IFN-γ and TNF-α in bone marrow-derived macrophages. When studied in vivo in a CT26 murine colon tumor model, Western blot analysis of tumors revealed decreased levels of inflammation-related factors NF-κB p65 and MMP2. Additional flow cytometry analysis further showed a reduction in ROS, increased overall proportion of immune cells, a reduction in inflammation-associated MDSCs, and enhanced adaptive immune responses, including higher infiltration of CD8^+^ TILs, increased IFN-γ–positive CD8^+^ TILs, and elevated levels of antigen-presenting DCs. Building on these effects, the researchers next evaluated the synergistic antitumor activity of aPD-L1-loaded BetP-Gel. In the CT26 tumor model, aPDL1@BetP-Gel was found to significantly delay tumor growth and prolong survival compared to either BetP-Gel or free aPD-L1 alone. Notably, the treatment also induced systemic immune responses: in a bilateral tumor model, local injection of aPDL1@BetP-Gel suppressed growth of distant, untreated tumors, with enhanced granzyme B positive-CD8^+^ T cell infiltration observed at the secondary site.

This innovative approach demonstrates that anti-inflammatory hydrogels can effectively reprogram the TME, reducing chronic inflammation while simultaneously enhancing antitumor adaptive immune responses. Additionally, by combining local TME modulation with sustained immunotherapy delivery, these materials can potentiate both primary and systemic antitumor immunity. Given these promising results, further exploration of hydrogel-based strategies to selectively modulate inflammation warrants greater investigation.

#### 4.2.4. Intra and Extracellular Signaling

The TME is sustained by a web of intra- and extracellular signaling pathways that dictate immune cell positioning, activation, and function. Strategies that directly target these pathways, whether through blocking suppressive cues or amplifying stimulatory ones, can help shift the balance in favor of effective anti-tumor immunity.

One notable strategy has been to modulate cytokine signaling within the TME to reprogram local immune dynamics. In one example, researchers engineered a bacteria-derived hydrogel that displayed a triple-negative breast cancer-specific antigen (α-lactalbumin) and released both the chemokine CXCL10 (for T cell recruiting) and the CXCR4 ectodomain (CXCR4E) [178,182]. The inclusion of CXCR4E was intended to block the suppressive pathway driven by cancer-associated fibroblast secretion of CXCL12, which binds CXCR4 on T cells to hinder their tumor engagement and dampen responsiveness to CXCL10. In migration assays, hydrogel-released CXCL10 markedly enhanced T cell chemotaxis, while CXCR4E reversed the inhibitory effects of CXCL12, thereby restoring T cell responsiveness. Along similar lines, Xu et al. developed a sodium alginate hydrogel loaded with linagliptin, which inhibits CXCL10 degradation mediated by dipeptidyl peptidase 4 (DPP4) and promotes greater T cell infiltration, as well as BMS-202 particles to block PD-L1 [181]. In vivo evaluation demonstrated that the hydrogel effectively reduced DPP4 activity, resulting in higher local concentrations of CXCL10. This effect translated to enhanced anti-tumor activity in a mouse bilateral tumor model, particularly when combined with exogenous CXCL10 administration. Complementing these strategies, Gao et al. developed an injectable hydrogel containing RNA-loaded lipid nanoparticles designed to reshape the pancreatic TME [280]. The nanoparticles were loaded with immune regulatory factor 5 (IRF5) mRNA to increase the polarization of M2 macrophages to an M1 phenotype and CCL5 siRNA to inhibit the recruitment of TAMs to the area. Western blot analysis of hydrogel-treated tumors in mice showed both an increase in IRF5 and a decrease in CCL5 compared to controls, which coincided with a significant reduction in tumor growth.

Building on the idea of signal-targeted strategies, hydrogels have also been used to manipulate metabolic pathways within the TME to support immune activation. In one study by Wang et al., a supramolecular in situ hydrogel was co-loaded with both DOX and kynureninase (KYNU) to disrupt tryptophan catabolite kynurenine immunosuppressive pathways in the TME [281]. In vitro experiments demonstrated that the hydrogel could effectively eliminate kynurenine within 3 days of treatment, with sustained depletion observed for up to 10 days post-treatment depending on the hydrogel composition. When studied in vivo, tumors treated with KYNU alone in the highest-percentage hydrogel formulation showed significant growth inhibition, and mice exhibited approximately a 28% increase in median survival relative to those receiving three doses of free KYNU. In a separate study, another group targeted a different metabolic checkpoint by reshaping the adenosinergic axis, a pathway through which extracellular adenosine accumulates in the TME and dampens antitumor immunity [282,283]. Towards this aim, Gao et al. designed an injectable sodium alginate hydrogel containing adenosine deaminase (ADA), the autophagy inducer benzene-1,2,3-tricarboxylic acid (BTC), and DOX. Upon injection, DOX and BTC synergized to induce ICD and ATP release, while ADA catalyzed the conversion of adenosine into inosine, an alternative energy source for T cells, thereby both reducing immunosuppressive adenosine accumulation and generating an immune-potentiating metabolite. In tumor-bearing mice, this strategy successfully reversed the negative feedback of the adenosinergic axis, increasing inosine levels ninefold, while also enhancing T cell activation, and producing significant tumor regression.

In addition to cytokine and metabolic pathways, hydrogels have also been applied to modulate signaling networks that control tumor vascularization and immune checkpoint activity. For example, a thermo-sensitive PLGA-PEG-PLGA triblock hydrogel was used to locally deliver lenvatinib, a multikinase inhibitor targeting VEGFR1–3 alongside a PD-1 monoclonal antibody (P&L@Gel) [260]. By inhibiting VEGFR1–3, researchers aimed to suppress angiogenesis and normalize tumor vasculature, facilitating improved immune cell infiltration. In balb/c mice bearing CT26 tumors, multiple doses of P&L@Gel led to a pronounced reduction in CD31 on vascular endothelium, a 2.2-fold increase in levels of CD8^+^ T cells, as well as marked polarization of TAMs from the M2 to the M1 phenotype.

In another approach, Li et al. developed an injectable hydrogel to create a PD-L1 checkpoint-regulatable immune niche that simultaneously targets both tumor-associated and circulating exosomal PD-L1 [284]. The hydrogel, formed from oxidized sodium alginate-armored tumor membrane vesicles (O-TMV) in combination with a Ca^2+^ channel inhibitor (DMA) and a Cdk5 inhibitor, roscovitine (ROSCO), acted as both an antigen depot and as a modulator of Ca^2+^-dependent exosome secretion. This strategy utilized DMA to inhibit cellular Ca^2+^ influx into tumor cells, reducing exosomal release, while ROSCO downregulated PD-L1 expression in both tumor cells and exosomes and disrupted the feedback loop between PD-L1 upregulation and interferon-γ signaling. In vitro, O-TMV@DR hydrogels significantly reduced both surface and exosomal PD-L1 levels, even under IFN-γ stimulation, demonstrating its ability to overcome adaptive PD-L1 resistance mechanisms. This effect was further supported by measured reductions in both intracellular Ca^2+^ and exosome secretion. In vivo, this comprehensive reprogramming of PD-L1 and exosomal PD-L1 alleviated systemic immunosuppression, enhanced CD8^+^ T cell infiltration and activation, and significantly inhibited tumor growth.

Overall, these studies illustrate the diverse strategies by which hydrogels can modulate intra- and extracellular signaling within the TME. By targeting cytokine gradients, metabolic checkpoints, angiogenic pathways, and immune checkpoint signaling, hydrogel platforms can reprogram tumor promoting immunosuppressive networks. Such approaches not only enhance immune cell infiltration and activation, but also overcome adaptive resistance mechanisms, highlighting the potential of signal-targeted hydrogel therapies to improve antitumor immunity.

### 4.3. Formation of Tertiary Lymphoid-like Structures

Tertiary lymphoid structures (TLS), also known as tertiary lymphoid organs or ectopic lymphoid structures, are organized aggregates of immune cells that form in non-lymphoid tissues in response to persistent, localized chronic inflammation. While they share structural and functional similarities with secondary lymphoid organs such as lymph nodes, TLS do not exist under normal physiological conditions, but rather arise transiently in response to pathogenic or malignant threats [285]. Once established, TLSs facilitate local antigen presentation, T cell priming, and B cell maturation, and their presence has been correlated with improved prognosis and responsiveness to immunotherapy [285,286]. Hydrogels have emerged as particularly promising platforms for promoting the formation of TLS-like structures, which contain a variety of immune cell types. They can serve not only as physical scaffolds, but also as active immune niches that deliver chemotactic cues and structural support to recruit and organize diverse immune cell populations.

One strategy for inducing TLS formation with hydrogels involves the controlled delivery of immune signals to recruit and organize host immune cells. For example, Kuwentrai et al. developed an injectable hydrogel formulated from supramolecular interactions between 4-(4-chlorophenyl)pyridine modified HA (HA-CPP) and cucurbit[8]uril (CB [8]) designed to promote TLS generation through delivery of soluble signals [287]. The hydrogel released TLS-inducing cytokines CXCL13 and LIGHT to recruit B and T cells respectively. After i.p. injection of both the hydrogel and the tumor, this formulation successfully recruited T cells and B cells to the tumor site, where they organized into TLS-like structures characterized by distinct inner and outer B and T-cell zones, with greater than 50% of these structures showing indicators of mature TLS formation. Functionally, the presence of these TLSs increased IFN-γ production by splenocytes and improved immune responses against the primary tumor.

**Figure 5:**
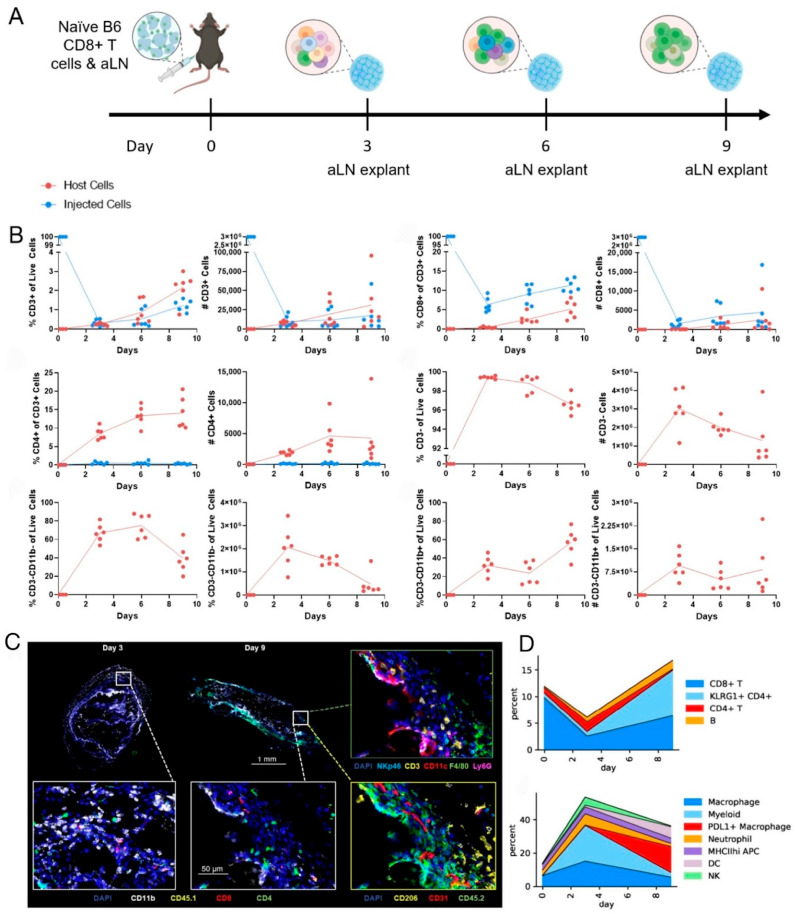
Example of tertiary-like lymphoid structure generation over time: (**A**) Murine treatment protocol showing injection of aLN with naive multiclonal CD8 T cells (multi-color cells) on day 0 with continued enrichment of tumor specific cells (green cells) over the course of 9 days (**B**) Evaluation of host versus transferred cells by flow cytometry on days 0, 3, and 9 by cell type. (**C**) Representative CODEX image of explanted aLN on days 3 and 9. (**D**) Stacked line chart showing percentage of immune cells present in aLN on days 0, 3, and 9. Reprinted with permission from Livingston, N.K. et al. “In Vivo Stimulation of Therapeutic Antigen-Specific T Cells in an Artificial Lymph Node Matrix” Advanced Materials, (2024) 36(23) © Copyright [169] Wiley-VCH GmbH or related companies. All rights reserved, including rights for text and data mining and training of artificial intelligence technologies or similar technologies.

Another approach taken by researchers leverages hydrogels as active niches to directly present activating signals to T cells, which in turn recruit and organize additional immune cells. For instance, Livingston et al. developed an artificial lymph node (aLN) matrix by encapsulating naïve T cells within an injectable thiolated-HA hydrogel crosslinked with PEGDA [169]. The aLN was functionalized with immobilized T cell–activating cues, including anti-CD3, anti-CD28, and IL-2-bound antibodies, allowing the hydrogel to serve as both a structural scaffold and an activation platform. Crucially, upon subcutaneous injection, the encapsulated T cells became activated and proliferated, releasing chemokines and cytokines that recruited host immune cells into the matrix. Over time, the structure transitioned from an initial wound-healing–like niche, characterized by high myeloid and macrophage infiltration on day 3, into a more organized anti-tumor immune niche with increased T cell percentages and a small population of B cells by day 9 (Figure 5). This strategy was shown to improve antigen-specific T cell responses and demonstrated significant inhibition of tumor growth in multiple tumor models, highlighting the potential of combining cellular delivery with hydrogel-based immune programming to generate TLS-like structures.

In addition to delivering soluble or cellular cues, the intrinsic chemical and physical properties of hydrogels themselves can be tuned to influence the formation of TLS-like immune niches and immune cell organization. For example, Ding et al. investigated chiral polypeptide hydrogels composed of poly(γ-ethyl-L-glutamate) (L-Gel) or poly(γ-ethyl-D-glutamate) (D-Gel), finding that chirality significantly affected the local immune microenvironment [288]. While D-Gel recruited more immune cells, these cells exhibited higher levels of suppressive markers (PD-L1 and PD-1) and signs of T cell exhaustion (PD-1, LAG-3, and TIM-3), resulting in a chronically inflamed but immunosuppressive environment with limited anti-tumor activity. In contrast, L-Gel induced a milder immune response that supported tumor inhibition. Similar trends were observed in comparisons of PEG-poly(L-alanine) versus mPEG-poly(D-alanine) and mPEG-poly(L-valine) versus mPEG-poly(D-valine)-based hydrogels. Adopting a complementary strategy, a different group engineered immune hydrogel niches (iHGs) using PVA supplemented with one of five distinct chemical moieties: alginate (iHG-ALG), chitosan (iHG-CS), galactomannan (iHG-GM), gelatin (iHG-GE), or polyethyleneimine (iHG-EI) to study how variations in the hydrogel affected the immune cell response [289]. Implanted at a site distant from the TME, these hydrogels recruited a broad range of immune populations, including DCs, macrophages, NK cells, B cells, and T cells. Notably, the specific chemical composition of each hydrogel strongly influenced the cellular makeup. For example, five days after implantation, neutrophils ranged anywhere from 14.8–60.4% and macrophages from 7.4–37.2% of total hydrogel infiltrating cells. These differences were reflected in systemic cytokine profiles as well as local levels of pro-inflammatory cytokines such as IL-1β, TNF-α, and IL-6 within the gels. Ultimately, these results demonstrate that hydrogel composition is not a passive scaffold feature, but one that can be harnessed to shape the formation of artificial TLS-like structures.

Together, these studies highlight the versatility of hydrogel-based scaffolds in programming de novo immune niches. By guiding TLS-like formation, hydrogels influence both the cellular architecture and functional output of these niches, promoting more effective local immune responses against tumors. This strategy represents a powerful new frontier for hydrogel engineering, and future work should aim to fine-tune hydrogel properties to control TLS persistence, spatial organization, and integration with clinical immunotherapies.

## 5. Human Clinical Trials

Hydrogel-based platforms have entered the clinical landscape for oncology both as approved products and through a growing range of human trials. Importantly, many of the hydrogel systems currently in clinical use in cancer patients are not designed to directly modulate the immune system, functioning instead as drug depots, protective spacers, fiducial markers, or topical protectants. However, newer hydrogel–cell combination products are also emerging that do aim to engage or modulate immune responses.

Several hydrogel formulations have already received regulatory approval in cancer-related settings. One notable example is Jelmyto (UroGen Pharma, Princeton, NJ, USA), a thermosensitive hydrogel formulation containing Pluronic F-127, PEG-400, and HPMC), loaded with mitomycin, which gained FDA approval in 2020 for the treatment of low-grade upper tract urothelial carcinoma. By enabling catheter-based instillation into the urinary tract, Jelmyto allows localized chemotherapy exposure while limiting systemic distribution [290,291]. Another approved product, Vantas (Endo Pharmaceuticals, Malvern, PA, USA), is a hydrogel depot composed of hydroxyethyl methacrylate (HEMA) and hydroxypropyl methacrylate (HPMA), with trimethylolpropane trimethacrylate (TMPTMA) as the crosslinker, that releases histrelin acetate for the palliative management of advanced prostate cancer [291]. It was approved by the FDA in 2004 and by the EMA in 2005, and provides up to 12 months of continuous hormone suppression following a single subcutaneous administration. Additionally, SpaceOAR Hydrogel (Augmenix, Malborough, MA, USA), a PEG-based spacer, was approved by the FDA in 2015 and EMA in 2010 for use in protecting vulnerable tissues during prostate cancer radiotherapy. By creating a temporary physical barrier between the prostate and rectum, SpaceOAR significantly reduces rectal irradiation, thereby lowering rectal toxicity during high-dose radiation [291,292]. Collectively, these examples highlight the diverse strategies by which hydrogels have already reached patients, either as drug depots, protective spacers, or local delivery matrices.

Some hydrogels that are already approved continue to be studied for expanded indications, while other platforms are still progressing through evaluation. For example, SpaceOAR is currently being investigated in six ongoing clinical trials in prostate radiotherapy, focusing on dosimetry, safety, and clinical outcomes [291]. Previous studies have already confirmed reductions in long-term rectal toxicity and improvements in urinary and sexual quality of life, and ongoing investigations are assessing its use across conventional and stereotactic regimens, although evidence in hypofractionated settings remains limited [291,293]. Additionally, TraceIT (Augmenix), a PEG-based hydrogel fiducial marker approved by the FDA in 2013 for soft-tissue marking, is undergoing clinical evaluation in pancreatic radiotherapy where it is being tested for tumor imaging and alignment, including studies marking pancreas duodenum interface for image guided stereotactic body radiotherapy [294].

In parallel, several hydrogel platforms are undergoing clinical evaluation. LifePearl, from Grupo Espanol Multidisciplinario del Cancer Digestivo (Madrid, Spain), is a PEG-based hydrogel microsphere suspension currently in a Phase II trial for the intra-arterial delivery of irinotecan in patients with colorectal liver metastases (NCT04595266) [291,295]. Topical hydrogels for the prevention and management of radiation dermatitis include StrataXRT from Stratapharma AG (Basel, Switzerland) (topical silicone; non-injectable hydrogel; NCT05553392) for radiation dermatitis and RadiaAce from AceTech (Tel Aviv-Yafo, Israel) (topical acemannan; non-injectable hydrogel; NCT04481802) for breast cancer radiation dermatitis, both listed as phase not applicable [291]. At an earlier stage, therapeutic depot systems are only now entering human testing, exemplified by TumoCure from IntraGel Therapeutics (Nazareth, Israel), an intratumoral cisplatin delivery hydrogel currently in a Phase I first-in-human study (NCT05200650) [291].

Beyond these hydrogel systems, which are primarily used for chemokine delivery or designed as non-injectable formulations, several hydrogel platforms that encapsulate and deliver cells are also being evaluated in clinical trials across diverse therapeutic areas. CartiLife from Biosolution (Gaithersburg, MD, USA) is a fibrin microsphere hydrogel encapsulating chondrocytes, currently in a Phase III trial for intraarticular treatment of articular cartilage defects (NCT05051332) [291]. SygeLIX-F and SygeLIX-G from TBF Genie Tissulaire (Mions, France) employ a Wharton’s Jelly–based gel plug carrying stem cells for the treatment of anal fistula and are being tested in a Phase I study (NCT05638139) [291]. BioVAT from University Medical Center Göttingen (Göttingen, Germany) delivers iPSC-derived cardiomyocytes within a bulk collagen hydrogel implanted into the ventricular myocardium, now in Phase I/II testing for heart failure (NCT04396899) [291]. A placental MSC-loaded dural ECM hydrogel graft, developed by the California Institute for Regenerative Medicine, delivers mesenchymal stem cells and is under Phase I/II evaluation for myelomeningocele (NCT04652908) [291]. Finally, ALLO-ACS-DFU from Anterogen (Seoul, Republic of Korea) is a hydrogel sheet dressing seeded with allogeneic mesenchymal stem cells in a Phase II trial for diabetic foot ulcers (NCT03754465) [291]. A summary of FDA/EMA-approved hydrogel systems in cancer therapy and those still under clinical evaluation are provided in Table 2 and Table 3, respectively.

Taken together, these clinical experiences suggest that hydrogels are poised to become an integral component of cancer therapy. Approved products already demonstrate clinical benefit by reducing collateral tissue injury and prolonging local drug action, while ongoing trials continue to explore hydrogels as drug depots, fiducial markers, radiosensitizer adjuncts, topical protectants, and platforms for cell delivery. The translation of these systems from bench to bedside underscores their clinical potential in reshaping oncologic treatment paradigms, particularly by enhancing precision and mitigating toxicity.

## 6. Conclusions

Hydrogel-based platforms represent a highly versatile and modular approach for cancer immunotherapy, capable of integrating principles of material design with precise immune modulation. By leveraging biocompatibility and tunable biodegradability, these systems can provide safe and transient scaffolds that support localized immune activation while minimizing off-target effects. Control over degradation kinetics, mechanical properties, and spatiotemporal release allows for the sustained and coordinated presentation of cytokines, chemokines, antigens, or checkpoint inhibitors. Injectability and in vivo gelation facilitate minimally invasive administration and integration into the TME. Immune-specific functionalization enables hydrogels to directly engage T cells, DCs, or macrophages, enhancing cellular activation, recruitment, and cross-talk. Preclinical studies demonstrate that such platforms can improve T cell infiltration, promote DC maturation and cross-presentation, intercept CTCs, and reprogram suppressive tumor niches. Taken together, these findings highlight how rationally designed hydrogels that combine material properties with precise immunological cues offer a powerful strategy to overcome key barriers in cancer therapy. Future work focused on optimization, mechanistic understanding, and translational considerations will be essential to fully realize their potential in clinical immunoengineering.

## Figures and Tables

**Table 1:** Representative chemical structures of commonly used natural and synthetic hydrogel backbones. The table also compares their biocompatibility, biodegradability, and degradation by-products.

Property	Natural Hydrogels	Synthetic Hydrogels
Backbone	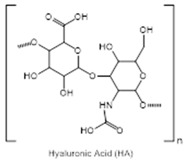 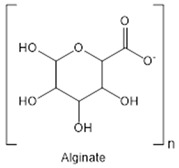 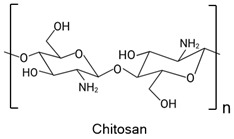	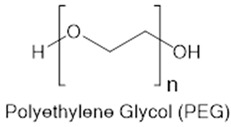 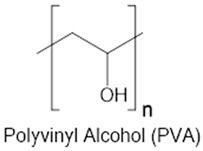 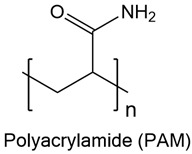
Biocompatibility	High, mimic extracellular matrix, support cell adhesion and signaling Examples: HA, collagen, alginate.	Inert, require functionalization Examples: PEG, PVA improved cell compatibility with RGD peptides
Biodegradability	Enzymatic degradation (e.g., HA by hyaluronidases; collagen by collagenases; alginate by lyases).	Non-degradable unless cleavable linkers included (ester, peptide, disulfide)
Degradation by-products	Bioactive fragments, may trigger immune responses (e.g., HA < 500 kDa activates TLR2/4)	Usually inert or low-molecular-weight alcohols/acids, immunologically quiescent but potential osmotic stress if accumulated

**Table 2:** FDA/EMA-approved hydrogel systems in cancer therapy.

Name (Company)	Material/Composition	Role in Therapy	Indication(s)	Regulatory Status
Jelmyto (UroGen Pharma)	Pluronic F-127 + PEG-400 + HPMC/mitomycin delivery	Chemotherapy localized depot	Low-grade upper tract urothelial carcinoma	FDA (2020) approved
Vantas (Endo Pharmaceuticals)	HEMA + HPMA + TMPTMA/histrelin acetate delivery	Hormone suppression depot	Advanced prostate cancer	FDA (2004), EMA (2005) approved
SpaceOAR (Augmenix)	PEG hydrogel spacer	Radioprotective spacer on vulnerable tissues	Prostate cancer radiotherapy	FDA (2015), EMA (2010) approved
TraceIT (Augmenix)	PEG hydrogel	Precise alignment of soft tissues	Soft-tissue marking in image-guided radiotherapy	FDA (2013) approved

**Table 3:** Hydrogel systems under clinical evaluation in cancer therapy.

Name (Company)	Material/Composition	Role in Therapy	Indication(s)	Trial Phase
LifePearl (Grupo Espanol Multidisciplinario del Cancer Digestiv)	PEG hydrogel microspheres	Intraarterial embolic chemotherapy	Colorectal liver metastases	Phase II
StrataXRT (Stratapharma)	Silicone hydrogel barrier	Topical protectant against dermatitis	Radiation dermatitis (breast, head & neck cancer)	NA
RadiaAce (AceTech)	Acemannan-containing hydrogel	Anti-inflammatory/epithelial repair	Radiation dermatitis (breast, head & neck cancer)	NA
TumoCure (IntraGel Therapeutics)	Bulk polymeric depot/cisplatin	Intratumoral chemotherapy depot	Head and neck cancers	Phase I

## Data Availability

No new data were created or analyzed in this study. Data sharing is not applicable to this article.

## Abbreviations

The following abbreviations are used in this manuscript:

ICI	immune checkpoint inhibitors
aPD-1	anti-PD-1
ACT	adoptive cell therapy
TME	tumor microenvironment
DC	dendritic cell
CTC	circulating tumor cell
DAMP	damage-associated molecular patterns
TLR	Toll-like receptor
HA	hyaluronic acid
PEG	poly(ethylene glycol)
FBR	foreign-body response
PVA	polyvinyl alcohol
PAM	polyacrylamide
ECM	extracellular matrix
MMP-2	matrix metalloproteinase-2
aPD-L1	anti-PD-L1
DOX	doxorubicin
ICD	immunogenic cell death
ROS	reactive oxygen species
CTL	cytotoxic T lymphocyte
GelMA	methacrylate-gelatin
iNOS	inducible nitric oxide synthase
cDC	conventional DCs
pDC	plasmacytoid DC
TAM	Tumor-associated macrophage
dLN	draining lymph node
G′	storage moduli
G″	loss modulus
HCC	hepatocellular carcinoma
P407	poloxamer 407
RGD	Arg–Gly–Asp
MHC	major histocompatibility complex
Tregs	regulatory T cells
aTM	artificial T-cell stimulating matrix
TCR	T cell receptor
e-beam	electron beam
HCP	host cell protein
TIL	tumor-infiltrating lymphocyte
CAR	chimeric antigen receptor
HPMC	hydroxypropyl methylcellulose
GATI	gel anti-tumor T cell injection
BiTE	bispecific T cell engager
TDLN	tumor-draining lymph node
PEGDA	poly(ethylene glycol) diacrylate
BPB	biomimetic physical barrier
BMDC	bone marrow-derived DC
FLT3L	FMS-like tyrosine kinase 3 ligand
GM-CSF	granulocyte–macrophage colony-stimulating factor
CpG-ODN	CpG oligodeoxynucleotide
poly(I:C)	polyinosinic/polycytidylic acid
Nap	Napabucasin
PDT	photodynamic therapy
PAMP	pathogen-associated molecular pattern
NO	nitric oxide
PP2	polyphyllin
R848	resiquimod
CAMKII	Ca2+/calmodulin-dependent protein kinase II
ICB	Immune checkpoint blockade
Tuni	tunicamycin
CAT	catalase
CRT	calreticulin
HIF	hypoxia-inducible factor
CHA	chlorogenic acid
CXCR4E	CXCR4 ectodomain
DPP4	dipeptidyl peptidase 4
IRF5	immune regulatory factor 5
KYNU	kynureninase
ADA	adenosine deaminase
BTC	benzene-1,2,3-tricarboxylic acid
ROSCO	roscovitine
TLS	tertiary lymphoid structures
aLN	artificial lymph node
HEMA	hydroxyethyl methacrylate
HPMA	hydroxypropyl methacrylate
TMPTMA	trimethylolpropane trimethacrylate

## References

[1] Wang R., Lan C., Benlagha K., Camara N.O.S., Miller H., Kubo M., Heegaard S., Lee P., Yang L., Forsman H. (2024). The interaction of innate immune and adaptive immune system. MedComm.

[2] Hossain M.A. (2024). A comprehensive review of immune checkpoint inhibitors for cancer treatment. Int. Immunopharmacol..

[3] Zhao D., Zhu D., Cai F., Jiang M., Liu X., Li T., Zheng Z. (2023). Current Situation and Prospect of Adoptive Cellular Immunotherapy for Malignancies. Technol. Cancer Res. Treat..

[4] Kichloo A., Albosta M., Dahiya D., Guidi J.C., Aljadah M., Singh J., Shaka H., Wani F., Kumar A., Lekkala M. (2021). Systemic adverse effects and toxicities associated with immunotherapy: A review. World J. Clin. Oncol..

[5] Parums D.V. (2024). Editorial: First Regulatory Approval for Adoptive Cell Therapy with Autologous Tumor-Infiltrating Lymphocytes (TILs)—Lifileucel (Amtagvi). Med. Sci. Monit. Int. Med. J. Exp. Clin. Res..

[6] Kuczek D.E., Larsen A.M.H., Thorseth M.-L., Carretta M., Kalvisa A., Siersbæk M.S., Simões A.M.C., Roslind A., Engelholm L.H., Noessner E. (2019). Collagen density regulates the activity of tumor-infiltrating T cells. J. Immunother. Cancer.

[7] Laoui D., Keirsse J., Morias Y., Van Overmeire E., Geeraerts X., Elkrim Y., Kiss M., Bolli E., Lahmar Q., Sichien D. (2016). The tumour microenvironment harbours ontogenically distinct dendritic cell populations with opposing effects on tumour immunity. Nat. Commun..

[8] Kim W., Chu T.H., Nienhüser H., Jiang Z., Del Portillo A., Remotti H.E., White R.A., Hayakawa Y., Tomita H., Fox J.G. (2020). PD-1 Signaling Promotes Tumor-Infiltrating Myeloid-Derived Suppressor Cells and Gastric Tumorigenesis in Mice. Gastroenterology.

[9] Rodríguez-Cid J.R., Chards S.C.-C., González-Espinoza I.R., García-Montes V., Garibay-Díaz J.C., Hernández-Flores O., Riera-Sala R., Gozalishvili-Boncheva A., Alatorre-Alexander J.A., Martínez-Barrera L.M. (2021). A comparative study of immunotherapy as second-line treatment and beyond in patients with advanced non-small-cell lung carcinoma. Lung Cancer Manag..

[10] Falchero L., Meyer N., Molinier O., Al Freijat F., Pegliasco H., Lecuyer E., Stoven L., Belmont L., Loutski S., Maincent C. (2024). Real-life nationwide characteristics and outcomes of small cell lung cancer over the last 20 years: Impact of immunotherapy on overall survival in a real-life setting. Eur. J. Cancer.

[11] Anassi E., Ndefo U.A. (2011). Sipuleucel-T (provenge) injection: The first immunotherapy agent (vaccine) for hormone-refractory prostate cancer. Pharm. Ther..

[12] Zhu J., Marchant R.E. (2011). Design properties of hydrogel tissue-engineering scaffolds. Expert Rev. Med. Devices.

[13] Cha B., Shin S.R., Leijten J., Li Y., Singh S., Liu J.C., Annabi N., Abdi R., Dokmeci M.R., Vrana N.E. (2017). Integrin–Mediated Interactions Control Macrophage Polarization in 3D Hydrogels. Adv. Healthc. Mater..

[14] Martin K.E., García A.J. (2021). Macrophage phenotypes in tissue repair and the foreign body response: Implications for biomaterial-based regenerative medicine strategies. Acta Biomater..

[15] Saraswathibhatla A., Indana D., Chaudhuri O. (2023). Cell–extracellular matrix mechanotransduction in 3D. Nat. Rev. Mol. Cell Biol..

[16] Liu Y., Xiao L., Joo K.-I., Hu B., Fang J., Wang P. (2014). In Situ Modulation of Dendritic Cells by Injectable Thermosensitive Hydrogels for Cancer Vaccines in Mice. Biomacromolecules.

[17] Liu Q., Zhang D., Qian H., Chu Y., Yang Y., Shao J., Xu Q., Liu B. (2020). Superior Antitumor Efficacy of IFN-α2b-Incorporated Photo-Cross-Linked Hydrogels Combined with T Cell Transfer and Low-Dose Irradiation Against Gastric Cancer. Int. J. Nanomed..

[18] Liu Y., Liu F., Zeng Y., Lin L., Yu H., Zhang S., Yang W. (2024). Hydrogel systems for spatiotemporal controlled delivery of immunomodulators: Engineering the tumor immune microenvironment for enhanced cancer immunotherapy. Front. Cell Dev. Biol..

[19] Song H., Yang P., Huang P., Zhang C., Kong D., Wang W. (2019). Injectable polypeptide hydrogel-based co-delivery of vaccine and immune checkpoint inhibitors improves tumor immunotherapy. Theranostics.

[20] Medina J.D., Barber G.F., Coronel M.M., Hunckler M.D., Linderman S.W., Quizon M.J., Ulker V., Yolcu E.S., Shirwan H., García A.J. (2022). A hydrogel platform for co-delivery of immunomodulatory proteins for pancreatic islet allografts. J. Biomed. Mater. Res. Part A.

[21] Chen Y., Sun J., Huang Y., Lu B., Li S. (2018). Improved Cancer Immunochemotherapy via Optimal Co-delivery of Chemotherapeutic and Immunomodulatory Agents. Mol. Pharm..

[22] Moura D., Rohringer S., Ferreira H.P., Pereira A.T., Barrias C.C., Magalhães F.D., Bergmeister H., Gonçalves I.C. (2024). Long-term in vivo degradation and biocompatibility of degradable pHEMA hydrogels containing graphene oxide. Acta Biomater..

[23] Spoddig V., Murkar R.S., Kopp S., Walles H. (2025). Biomimetic hydrogel scaffolds for stimulating fibrotic responses: Development of an in-vitro assay for implant material testing. Front. Bioeng. Biotechnol..

[24] Kyriakides T.R., Kim H.-J., Zheng C., Harkins L., Tao W., Deschenes E. (2022). Foreign body response to synthetic polymer biomaterials and the role of adaptive immunity. Biomed. Mater..

[25] Carnicer-Lombarte A., Chen S.-T., Malliaras G.G., Barone D.G. (2021). Foreign Body Reaction to Implanted Biomaterials and Its Impact in Nerve Neuroprosthetics. Front. Bioeng. Biotechnol..

[26] Gil-Cantero S., Künig S., Aigner-Radakovics K., Steinberger P., Boccaccini A., Stöckl J. (2026). Recognition of degradation-derived products from biomedical scaffolds by Toll-like receptors. Biomaterials.

[27] Lee J.-H., Shin S.-J., Knowles J.C., Lee H.-H., Kim H.-W. (2024). Adaptive immunity of materials: Implications for tissue healing and regeneration. Bioact. Mater..

[28] Yang D., Xiao J., Wang B., Li L., Kong X., Liao J. (2019). The immune reaction and degradation fate of scaffold in cartilage/bone tissue engineering. Mater. Sci. Eng. C.

[29] Coutinho-Wolino K.S., Almeida P.P., Mafra D., Stockler-Pinto M.B. (2022). Bioactive compounds modulating Toll-like 4 receptor (TLR4)-mediated inflammation: Pathways involved and future perspectives. Nutr. Res..

[30] Ma M., Jiang W., Zhou R. (2024). DAMPs and DAMP-sensing receptors in inflammation and diseases. Immunity.

[31] Kolb J.P., Oguin T.H., Oberst A., Martinez J. (2017). Programmed Cell Death and Inflammation: Winter Is Coming. Trends Immunol..

[32] Powell J.D., Horton M.R. (2005). Threat matrix. Immunol. Res..

[33] Tavianatou A.G., Caon I., Franchi M., Piperigkou Z., Galesso D., Karamanos N.K. (2019). Hyaluronan: Molecular size-dependent signaling and biological functions in inflammation and cancer. FEBS J..

[34] Yang D., Jones K.S. (2008). Effect of alginate on innate immune activation of macrophages. J. Biomed. Mater. Res. Part A.

[35] Lee K.Y., Mooney D.J. (2012). Alginate: Properties and biomedical applications. Prog. Polym. Sci..

[36] Ménard M., Dusseault J., Langlois G., Baille W.E., Tam S.K., Yahia L., Zhu X.X., Hallé J. (2010). Role of protein contaminants in the immunogenicity of alginates. J. Biomed. Mater. Res. Part B Appl. Biomater..

[37] Ibrahim M., Ramadan E., Elsadek N.E., Emam S.E., Shimizu T., Ando H., Ishima Y., Elgarhy O.H., Sarhan H.A., Hussein A.K. (2022). Polyethylene glycol (PEG): The nature, immunogenicity, and role in the hypersensitivity of PEGylated products. J. Control. Release.

[38] Padín-González E., Lancaster P., Bottini M., Gasco P., Tran L., Fadeel B., Wilkins T., Monopoli M.P. (2022). Understanding the Role and Impact of Poly (Ethylene Glycol) (PEG) on Nanoparticle Formulation: Implications for COVID-19 Vaccines. Front. Bioeng. Biotechnol..

[39] Charles P.T., Stubbs V.R., Soto C.M., Martin B.D., White B.J., Taitt C.R. (2009). Reduction of Non-Specific Protein Adsorption Using Poly(ethylene) Glycol (PEG) Modified Polyacrylate Hydrogels In Immunoassays for Staphylococcal Enterotoxin B Detection. Sensors.

[40] Swartzlander M.D., Barnes C.A., Blakney A.K., Kaar J.L., Kyriakides T.R., Bryant S.J. (2015). Linking the foreign body response and protein adsorption to PEG-based hydrogels using proteomics. Biomaterials.

[41] Qi Y., Chilkoti A. (2015). Protein–polymer conjugation—Moving beyond PEGylation. Curr. Opin. Chem. Biol..

[42] Ewald J., Blankenburg J., Worm M., Besch L., Unger R.E., Tremel W., Frey H., Pohlit H. (2020). Acid-Cleavable Poly(ethylene glycol) Hydrogels Displaying Protein Release at pH 5. Chem. A Eur. J..

[43] Zhang P., Sun F., Liu S., Jiang S. (2016). Anti-PEG antibodies in the clinic: Current issues and beyond PEGylation. J. Control. Release.

[44] Yang Q., Lai S.K. (2015). Anti-PEG immunity: Emergence, characteristics, and unaddressed questions. WIREs Nanomed. Nanobiotechnol..

[45] Cao H., Duan L., Zhang Y., Cao J., Zhang K. (2021). Current hydrogel advances in physicochemical and biological response-driven biomedical application diversity. Signal Transduct. Target. Ther..

[46] Hennink W., van Nostrum C. (2012). Novel crosslinking methods to design hydrogels. Adv. Drug Deliv. Rev..

[47] Tripathi A.S., Zaki M.E.A., Al-Hussain S.A., Dubey B.K., Singh P., Rind L., Yadav R.K. (2023). Material matters: Exploring the interplay between natural biomaterials and host immune system. Front. Immunol..

[48] Satchanska G., Davidova S., Petrov P.D. (2024). Natural and Synthetic Polymers for Biomedical and Environmental Applications. Polymers.

[49] Joyce K., Fabra G.T., Bozkurt Y., Pandit A. (2021). Bioactive potential of natural biomaterials: Identification, retention and assessment of biological properties. Signal Transduct. Target. Ther..

[50] Isaac A.H., Phillips S.Y.R., Ruben E., Estes M., Rajavel V., Baig T., Paleti C., Landsgaard K., Lee R.H., Guda T. (2024). Impact of PEG sensitization on the efficacy of PEG hydrogel-mediated tissue engineering. Nat. Commun..

[51] Lynn A.D., Blakney A.K., Kyriakides T.R., Bryant S.J. (2011). Temporal progression of the host response to implanted poly(ethylene glycol)-based hydrogels. J. Biomed. Mater. Res. Part A.

[52] Kim K.M., D’Elia A.M., Rodell C.B. (2024). Hydrogel-based approaches to target hypersensitivity mechanisms underlying autoimmune disease. Adv. Drug Deliv. Rev..

[53] Pereira R.V.S., EzEldeen M., Ugarte-Berzal E., Martens E., Malengier-Devlies B., Vandooren J., Vranckx J.J., Matthys P., Opdenakker G. (2023). Physiological fibrin hydrogel modulates immune cells and molecules and accelerates mouse skin wound healing. Front. Immunol..

[54] Cui R., Wu Q., Wang J., Zheng X., Ou R., Xu Y., Qu S., Li D. (2021). Hydrogel-By-Design: Smart Delivery System for Cancer Immunotherapy. Front. Bioeng. Biotechnol..

[55] Wang Z., Ye Q., Yu S., Akhavan B. (2023). Poly Ethylene Glycol (PEG)-Based Hydrogels for Drug Delivery in Cancer Therapy: A Comprehensive Review. Adv. Healthc. Mater..

[56] Zhou Y., Chen K., Cheng H., Zhang S. (2025). Recent Advances in Polysaccharide-Based Hydrogels for Tumor Immunotherapy. Gels.

[57] Liu M., Wang T., Wang X., Ren W., Zhang Y., Li L., Diao H. (2025). Multifunctional hydrogels: Therapeutic strategies and advances in inflammation. Eur. Polym. J..

[58] Zeng T., Chen L., Yoshitomi T., Kawazoe N., Yang Y., Chen G. (2025). Research Strategies and Methods of Hydrogels for Antitumor Drug Delivery. Biomedicines.

[59] Zhu B., Chen Y., Yang X., Zhu Y., Zhao Y., Liu Q., Wang B., Martin-Saldaña S., Wang Y., Duan W. (2025). Succinamide ester-containing adhesive hydrogels with controllable degradation for biomedical applications. Cell Biomater..

[60] Singh A., Qin H., Fernandez I., Wei J., Lin J., Kwak L.W., Roy K. (2011). An injectable synthetic immune-priming center mediates efficient T-cell class switching and T-helper 1 response against B cell lymphoma. J. Control. Release.

[61] Irvine D.J., Swartz M.A., Szeto G.L. (2013). Engineering synthetic vaccines using cues from natural immunity. Nat. Mater..

[62] Blume K.G., Forman S.J., Appelbaum F.R. (2008). Thomas’ Hematopoietic Cell Transplantation.

[63] Bentley E.R., Little S.R. (2021). Local delivery strategies to restore immune homeostasis in the context of inflammation. Adv. Drug Deliv. Rev..

[64] Zhang Q., Hu W., Guo M., Zhang X., Zhang Q., Peng F., Yan L., Hu Z., Tangthianchaichana J., Shen Y. (2024). MMP-2 Responsive Peptide Hydrogel-Based Nanoplatform for Multimodal Tumor Therapy. Int. J. Nanomed..

[65] Gu J., Zhao G., Yu J., Xu P., Yan J., Jin Z., Chem S., Wang Y., Zhang L.W., Wang Y. (2022). Injectable pH-responsive hydrogel for combinatorial chemoimmunotherapy tailored to the tumor microenvironment. J. Nanobiotechnol..

[66] Wang M., Hu Q., Huang J., Zhang F., Yao Z., Shao S., Zhao X., Liang T. (2023). In Situ Formed ROS-Responsive Hydrogel with STING Agonist and Gemcitabine to Intensify Immunotherapy Against Pancreatic Ductal Adenocarcinoma. Adv. Healthc. Mater..

[67] Zhuang Z., Zhang Y., Sun S., Li Q., Chen K., An C., Wang L., Van den Beucken J.J.J.P., Wang H. (2020). Control of Matrix Stiffness Using Methacrylate–Gelatin Hydrogels for a Macrophage-Mediated Inflammatory Response. ACS Biomater. Sci. Eng..

[68] Butenko S., Nagalla R.R., Guerrero-Juarez C.F., Palomba F., David L.-M., Nguyen R.Q., Gay D., Almet A.A., Digman M.A., Nie Q. (2024). Hydrogel crosslinking modulates macrophages, fibroblasts, and their communication, during wound healing. Nat. Commun..

[69] Guenther C. (2024). Stiffness regulates dendritic cell and macrophage subtype development and increased stiffness induces a tumor–associated macrophage phenotype in cancer co–cultures. Front. Immunol..

[70] Wu C., Zhang H., Guo Y., Sun X., Hu Z., Teng L., Zeng Z. (2024). Porous Hydrogels for Immunomodulatory Applications. Int. J. Mol. Sci..

[71] Eggermont L.J., Rogers Z.J., Colombani T., Memic A., Bencherif S.A. (2020). Injectable Cryogels for Biomedical Applications. Trends Biotechnol..

[72] Ben Djemaa I., Auguste S., Drenckhan-Andreatta W., Andrieux S. (2021). Hydrogel foams from liquid foam templates: Properties and optimisation. Adv. Colloid Interface Sci..

[73] Griffin D.R., Weaver W.M., Scumpia P.O., Di Carlo D., Segura T. (2015). Accelerated wound healing by injectable microporous gel scaffolds assembled from annealed building blocks. Nat. Mater..

[74] Mayer D.P., Nelson M.E., Andriyanova D., Filler R.B., Ökten A., Antao O.Q., Chen J.S., Scumpia P.O., Weaver W.M., Wilen C.B. (2024). A novel microporous biomaterial vaccine platform for long-lasting antibody mediated immunity against viral infection. J. Control. Release.

[75] Verbeke C.S., Mooney D.J. (2015). Injectable, Pore-Forming Hydrogels for In Vivo Enrichment of Immature Dendritic Cells. Adv. Healthc. Mater..

[76] Zetao C., Travis K., Rachael M., Ross C., Jiang C., Chengtie W., Yin X. (2016). Osteoimmunomodulation for the development of advanced bone biomaterials. Mater. Today.

[77] Karageorgiou V., Kaplan D. (2005). Porosity of 3D biomaterial scaffolds and osteogenesis. Biomaterials.

[78] Mukasheva F., Adilova L., Dyussenbinov A., Yernaimanova B., Abilev M., Akilbekova D. (2024). Optimizing scaffold pore size for tissue engineering: Insights across various tissue types. Front. Bioeng. Biotechnol..

[79] López-Serrano C., Côté-Paradis Y., Habenstein B., Loquet A., Le Coz C., Ruel J., Laroche G., Durrieu M.-C. (2024). Integrating Mechanics and Bioactivity: A Detailed Assessment of Elasticity and Viscoelasticity at Different Scales in 2D Biofunctionalized PEGDA Hydrogels for Targeted Bone Regeneration. ACS Appl. Mater. Interfaces.

[80] Roca-Arroyo A.F., Gutierrez-Rivera J.A., Morton L.D., Castilla-Casadiego D.A. (2025). Hydrogel Network Architecture Design Space: Impact on Mechanical and Viscoelastic Properties. Gels.

[81] Crandell P., Stowers R. (2023). Spatial and Temporal Control of 3D Hydrogel Viscoelasticity through Phototuning. ACS Biomater. Sci. Eng..

[82] Darnell M., Young S., Gu L., Shah N., Lippens E., Weaver J., Duda G., Mooney D. (2017). Substrate Stress-Relaxation Regulates Scaffold Remodeling and Bone Formation In Vivo. Adv. Healthc. Mater..

[83] Mai Y., Wang H., Lu J., Shi S., Cai Y., Zhang W., Xie S., Huang R., Ji S., Qu X. (2025). Catalyst-modulated hydrogel dynamics for decoupling viscoelasticity and directing macrophage fate for diabetic wound healing. Bioact. Mater..

[84] Tao D., Wang H., Chang S., Cheng J., Da N., Zhang L., Yang J., Wang W., Xu F., Li B. (2025). Matrix Viscoelasticity Orchestrates Osteogenesis via Mechanotransduction Mediated Metabolic Switch in Macrophages. Adv. Healthc. Mater..

[85] Kleponis J., Skelton R., Zheng L. (2015). Fueling the engine and releasing the break: Combinational therapy of cancer vaccines and immune checkpoint inhibitors. Cancer Biol. Med..

[86] Chua B.Y., Sekiya T., Al Kobaisi M., Short K.R., Mainwaring D.E., Jackson D.C. (2015). A single dose biodegradable vaccine depot that induces persistently high levels of antibody over a year. Biomaterials.

[87] Liu J., Fu M., Wang M., Wan D., Wei Y., Wei X. (2022). Cancer vaccines as promising immuno-therapeutics: Platforms and current progress. J. Hematol. Oncol..

[88] Cheng S.-L., Lee H.-M., Li C.-P., Lin M.-W., Chou M.-Y., Yen Y.-T., Wu T.-H., Lian Y.-C., Shih Y.-C., Chiang C.-S. (2024). Robust and Sustained STING Pathway Activation via Hydrogel-Based In Situ Vaccination for Cancer Immunotherapy. ACS Nano.

[89] Segovia N., Pont M., Oliva N., Ramos V., Borrós S., Artzi N. (2014). Hydrogel Doped with Nanoparticles for Local Sustained Release of siRNA in Breast Cancer. Adv. Healthc. Mater..

[90] Harui A., McLachlan S.M., Rapoport B., Zarembinski T.I., Roth M.D. (2020). Peri-tumor administration of controlled release anti-CTLA-4 synergizes with systemic anti-PD-1 to induce systemic antitumor immunity while sparing autoimmune toxicity. Cancer Immunol. Immunother..

[91] Yang A., Dong X., Bai Y., Sheng S., Zhang Y., Liu T., Zhu D., Lv F. (2021). Doxorubicin/CpG self-assembled nanoparticles prodrug and dendritic cells co-laden hydrogel for cancer chemo-assisted immunotherapy. Chem. Eng. J..

[92] Bhatta R., Han J., Liu Y., Bo Y., Wang H. (2023). T Cell-Responsive Macroporous Hydrogels for In Situ T Cell Expansion and Enhanced Antitumor Efficacy. Biomaterials.

[93] Lu P., Ruan D., Huang M., Tian M., Zhu K., Gan Z., Xiao Z. (2024). Harnessing the potential of hydrogels for advanced therapeutic applications: Current achievements and future directions. Signal Transduct. Target. Ther..

[94] Zhang Y., Wu B.M. (2023). Current Advances in Stimuli-Responsive Hydrogels as Smart Drug Delivery Carriers. Gels.

[95] Falcone N., Ermis M., Gangrade A., Choroomi A., Young P., Mathes T.G., Monirizad M., Zehtabi F., Mecwan M., Rodriguez M. (2024). Drug-Eluting Shear-Thinning Hydrogel for the Delivery of Chemo- and Immunotherapeutic Agents for the Treatment of Hepatocellular Carcinoma. Adv. Funct. Mater..

[96] Chung C.K., Fransen M.F., van der Maaden K., Campos Y., García-Couce J., Kralisch D., Chan A., Ossendorp F., Cruz L.J. (2020). Thermosensitive hydrogels as sustained drug delivery system for CTLA-4 checkpoint blocking antibodies. J. Control. Release.

[97] Cabanaa A., Ait-Kadi A., Juhász J. (1997). Study of the Gelation Process of Polyethylene Oxidea–Polypropylene Oxideb–Polyethylene OxideaCopolymer (Poloxamer 407) Aqueous Solutions. J. Colloid Interface Sci..

[98] Chen X., Wang M., Yang X., Wang Y., Yu L., Sun J., Ding J. (2019). Injectable hydrogels for the sustained delivery of a HER2-targeted antibody for preventing local relapse of HER2+ breast cancer after breast-conserving surgery. Theranostics.

[99] Chenite A., Chaput C., Wang D., Combes C., Buschmann M., Hoemann C., Leroux J., Atkinson B., Binette F., Selmani A. (2000). Novel injectable neutral solutions of chitosan form biodegradable gels in situ. Biomaterials.

[100] Qu J., Zhao X., Ma P.X., Guo B. (2017). pH-responsive self-healing injectable hydrogel based on N-carboxyethyl chitosan for hepatocellular carcinoma therapy. Acta Biomater..

[101] Gao K., Xu K. (2025). Advancements and Prospects of pH-Responsive Hydrogels in Biomedicine. Gels.

[102] Patroklou G., Triantafyllopoulou E., Goula P.-E., Karali V., Chountoulesi M., Valsami G., Pispas S., Pippa N. (2025). pH-Responsive Hydrogels: Recent Advances in Pharmaceutical Applications. Polymers.

[103] Lutolf M.P., Lauer-Fields J.L., Schmoekel H.G., Metters A.T., Weber F.E., Fields G.B., Hubbell J.A. (2003). Synthetic matrix metalloproteinase-sensitive hydrogels for the conduction of tissue regeneration: Engineering cell-invasion characteristics. Proc. Natl. Acad. Sci. USA.

[104] Yang Z., Ma M., Xu B. (2009). Using matrix metalloprotease-9 (MMP-9) to trigger supramolecular hydrogelation. Soft Matter.

[105] Carlini A.S., Gaetani R., Braden R.L., Luo C., Christman K.L., Gianneschi N.C. (2019). Enzyme-responsive progelator cyclic peptides for minimally invasive delivery to the heart post-myocardial infarction. Nat. Commun..

[106] Coulter S.M., Pentlavalli S., An Y., Vora L.K., Cross E.R., Moore J.V., Sun H., Schweins R., McCarthy H.O., Laverty G. (2024). In Situ Forming, Enzyme-Responsive Peptoid-Peptide Hydrogels: An Advanced Long-Acting Injectable Drug Delivery System. J. Am. Chem. Soc..

[107] Fernando I.P.S., Lee W., Han E.J., Ahn G. (2020). Alginate-based nanomaterials: Fabrication techniques, properties, and applications. Chem. Eng. J..

[108] Gleichmann M., Mattson M.P. (2011). Neuronal Calcium Homeostasis and Dysregulation. Antioxidants Redox Signal..

[109] Robertson W.G., Marshall R.W., Bowers G.N. (1981). Ionized Calcium in Body Fluids. CRC Crit. Rev. Clin. Lab. Sci..

[110] Schmitt C. (2022). Viscoelasticity Characterization of Cellink Alginate Gels Using ElastoSensTM Bio. https://rheolution.com/application-notes/mechanical-testing-of-cellink-alginate-gels/.

[111] Kaliampakou C., Lagopati N., Pavlatou E.A., Charitidis C.A. (2023). Alginate–Gelatin Hydrogel Scaffolds; An Optimization of Post-Printing Treatment for Enhanced Degradation and Swelling Behavior. Gels.

[112] Ferreira N.N., Ferreira L.M., Miranda-Gonçalves V., Reis R.M., Seraphim T.V., Borges J.C., Baltazar F., Gremião M.P.D. (2017). Alginate hydrogel improves anti-angiogenic bevacizumab activity in cancer therapy. Eur. J. Pharm. Biopharm..

[113] Chao Y., Liang C., Tao H., Du Y., Wu D., Dong Z., Jin Q., Chen G., Xu J., Xiao Z. (2020). Localized cocktail chemoimmunotherapy after in situ gelation to trigger robust systemic antitumor immune responses. Sci. Adv..

[114] Said N.S., Olawuyi I.F., Lee W.Y. (2023). Pectin Hydrogels: Gel-Forming Behaviors, Mechanisms, and Food Applications. Gels.

[115] Zhao X., Pan Y., Zhou Z., Gao Y., Li A., Shi B., Hu J., Wang L. (2025). Effect of Potassium-Ion-Triggered Double Helix Aggregation on Shakedown Behavior of κ-Carrageenan/Polyacrylamide Hydrogel. Gels.

[116] Salehi S., Naghib S.M., Garshasbi H.R., Ghorbanzadeh S., Zhang W. (2023). Smart stimuli-responsive injectable gels and hydrogels for drug delivery and tissue engineering applications: A review. Front. Bioeng. Biotechnol..

[117] Rinaudo M. (2006). Chitin and chitosan: Properties and applications. Prog. Polym. Sci..

[118] Berger J., Reist M., Mayer J., Felt O., Gurny R. (2004). Structure and interactions in chitosan hydrogels formed by complexation or aggregation for biomedical applications. Eur. J. Pharm. Biopharm..

[119] Jia S., Wang J., Wang X., Liu X., Li S., Li Y., Li J., Wang J., Man S., Guo Z. (2023). Genetically encoded in situ gelation redox-responsive collagen-like protein hydrogel for accelerating diabetic wound healing. Biomater. Sci..

[120] He J., Zhang A., Zhang Y., Guan Y. (2011). Novel Redox Hydrogel by in Situ Gelation of Chitosan as a Result of Template Oxidative Polymerization of Hydroquinone. Macromolecules.

[121] Ali A., Saroj S., Saha S., Rakshit T., Pal S. (2023). In Situ-Forming Protein-Polymer Hydrogel for Glucose-Responsive Insulin Release. ACS Appl. Bio Mater..

[122] Guan Y., Zhang Y. (2013). Boronic acid-containing hydrogels: Synthesis and their applications. Chem. Soc. Rev..

[123] Wang J., Wang Z., Yu J., Kahkoska A.R., Buse J.B., Gu Z. (2020). Glucose-Responsive Insulin and Delivery Systems: Innovation and Translation. Adv. Mater..

[124] Wang S., Chen Y., Ling Z., Li J., Hu J., He F., Chen Q. (2022). The role of dendritic cells in the immunomodulation to implanted biomaterials. Int. J. Oral Sci..

[125] Jha A., Moore E. (2024). Laminin-derived peptide, IKVAV, modulates macrophage phenotype through integrin mediation. Matrix Biol. Plus.

[126] Fernandez-Yague M.A., Hymel L.A., Olingy C.E., McClain C., Ogle M.E., García J.R., Minshew D., Vyshnya S., Lim H.S., Qiu P. (2022). Analyzing immune response to engineered hydrogels by hierarchical clustering of inflammatory cell subsets. Sci. Adv..

[127] Beskid N.M., Kolawole E.M., Coronel M.M., Nguyen B., Evavold B., García A.J., Babensee J.E. (2022). IL-10-Functionalized Hydrogels Support Immunosuppressive Dendritic Cell Phenotype and Function. ACS Biomater. Sci. Eng..

[128] Olson B.A., Mellody M., Soemardy C., Mao Z., Mei A., Lippert K., Hoffmann M.A.G., Di Carlo D., Mayo S.L. (2025). Functionalizing hydrogel nanovials with vesicles mimicking antigen-presenting vesicles and cancer exosomes improves T cell capture and activation. bioRxiv.

[129] Hickey J.W., Dong Y., Chung J.W., Salathe S.F., Pruitt H.C., Li X., Chang C., Fraser A.K., Bessell C.A., Ewald A.J. (2019). Engineering an Artificial T-Cell Stimulating Matrix for Immunotherapy. Adv. Mater..

[130] Galante R., Pinto T.J.A., Colaço R., Serro A.P. (2018). Sterilization of hydrogels for biomedical applications: A review. J. Biomed. Mater. Res. Part B Appl. Biomater..

[131] Tohfafarosh M., Baykal D., Kiel J.W., Mansmann K., Kurtz S.M. (2016). Effects of gamma and e-beam sterilization on the chemical, mechanical and tribological properties of a novel hydrogel. J. Mech. Behav. Biomed. Mater..

[132] Raina N., Pahwa R., Bhattacharya J., Paul A.K., Nissapatorn V., Pereira M.d.L., Oliveira S.M.R., Dolma K.G., Rahmatullah M., Wilairatana P. (2022). Drug Delivery Strategies and Biomedical Significance of Hydrogels: Translational Considerations. Pharmaceutics.

[133] Alonso J.M., Andrade del Olmo J., Perez Gonzalez R., Saez-Martinez V. (2021). Injectable Hydrogels: From Laboratory to Indus-trialization. Polymers.

[134] Correa S., Grosskopf A.K., Lopez Hernandez H., Chan D., Yu A.C., Stapleton L.M., Appel E.A. (2021). Translational Applications of Hydrogels. Chem. Rev..

[135] Segneanu A.E., Bejenaru L.E., Bejenaru C., Blendea A., Mogoşanu G.D., Biţă A., Boia E.R. (2025). Advancements in Hydrogels: A Comprehensive Review of Natural and Synthetic Innovations for Bio-medical Applications. Polymers.

[136] Erfani A., Diaz A.E., Doyle P.S. (2023). Hydrogel-enabled, local administration and combinatorial delivery of immunotherapies for cancer treatment. Mater. Today.

[137] Mandal A., Clegg J.R., Anselmo A.C., Mitragotri S. (2020). Hydrogels in the clinic. Bioeng. Transl. Med..

[138] Office of Regulatory Affairs (2018). Bacterial Endotoxins/Pyrogens.

[139] Williams K.L. (2007). Endotoxins: Pyrogens, LAL Testing and Depyrogenation.

[140] Jawa V., Joubert M.K., Zhang Q., Deshpande M., Hapuarachchi S., Hall M.P., Flynn G.C. (2016). Evaluating Immunogenicity Risk Due to Host Cell Protein Impurities in Antibody-Based Biotherapeutics. AAPS J..

[141] Rezaei Z., Yilmaz-Aykut D., Tourk F.M., Bassous N., Barroso-Zuppa M., Shawl A.I., Ashraf S.S., Avci H., Hassan S. (2022). Immunomodulating Hydrogels as Stealth Platform for Drug Delivery Applications. Pharmaceutics.

[142] Rana M., Demirkaya C., De la Hoz Siegler H. (2025). Beyond Needles: Immunomodulatory Hydrogel-Guided Vaccine Delivery Systems. Gels.

[143] Huang M., Huang Y., Liu H., Tang Z., Chen Y., Huang Z., Xu S., Du J., Jia B. (2022). Hydrogels for the treatment of oral and maxillofacial diseases: Current research, challenges, and future directions. Biomater. Sci..

[144] Boddupalli B.M., Mohammed Z.N.K., Nath R.A., Banji D. (2010). Mucoadhesive drug delivery system: An overview. J. Adv. Pharm. Technol. Res..

[145] Galassi C., Chan T.A., Vitale I., Galluzzi L. (2024). The hallmarks of cancer immune evasion. Cancer Cell.

[146] Sun L., Su Y., Jiao A., Wang X., Zhang B. (2023). T cells in health and disease. Signal Transduct. Target. Ther..

[147] Ugur M., Labios R.J., Fenton C., Knöpper K., Jobin K., Imdahl F., Golda G., Hoh K., Grafen A., Kaisho T. (2023). Lymph node medulla regulates the spatiotemporal unfolding of resident dendritic cell networks. Immunity.

[148] Gerner M.Y., Casey K.A., Kastenmuller W., Germain R.N. (2017). Dendritic cell and antigen dispersal landscapes regulate T cell immunity. J. Exp. Med..

[149] Chimen M., Apta B.H.R., Mcgettrick H.M., Rainger G.E., Mcgettrick H.M. (2017). Introduction: T Cell Trafficking in Inflammation and Immunity. T-Cell Trafficking: Methods and Protocols.

[150] Parums D.V. (2025). A Review of CAR T Cells and Adoptive T-Cell Therapies in Lymphoid and Solid Organ Malignancies. Med. Sci. Monit..

[151] Parsonidis P., Papasotiriou I. (2022). Adoptive Cellular Transfer Immunotherapies for Cancer. Cancer Treat. Res. Commun..

[152] Wang K., Chen Y., Ahn S., Zheng M., Landoni E., Dotti G., Savoldo B., Han Z. (2020). GD2-specific CAR T cells encapsulated in an injectable hydrogel control retinoblastoma and preserve vision. Nat. Cancer.

[153] Atik A.F., Suryadevara C.M., Schweller R.M., West J.L., Healy P., Ii J.E.H., Congdon K.L., Sanchez-Perez L., McLendon R.E., Archer G.E. (2018). Hyaluronic acid based low viscosity hydrogel as a novel carrier for Convection Enhanced Delivery of CAR T cells. J. Clin. Neurosci..

[154] Grosskopf A.K., Labanieh L., Klysz D.D., Roth G.A., Xu P., Adebowale O., Gale E.C., Jons C.K., Klich J.H., Yan J. (2022). Delivery of CAR-T cells in a transient injectable stimulatory hydrogel niche improves treatment of solid tumors. Sci. Adv..

[155] Chao Y., Wei T., Li Q., Liu B., Hao Y., Chen M., Wu Y., Song F., Chen Q., Liu Z. (2023). Metformin-containing hydrogel scaffold to augment CAR-T therapy against post-surgical solid tumors. Biomaterials.

[156] Li G., Du R., Wang D., Zhang X., Wang L., Pu S., Li X., Wang S., Zhang J., Liu B. (2025). Improved Efficacy of Triple-Negative Breast Cancer Immunotherapy via Hydrogel-Based Co-Delivery of CAR-T Cells and Mitophagy Agonist. Adv. Sci..

[157] Jie J., Mao D., Cao J., Feng P., Yang P. (2022). Customized Multifunctional Peptide Hydrogel Scaffolds for CAR-T-Cell Rapid Proliferation and Solid Tumor Immunotherapy. ACS Appl. Mater. Interfaces.

[158] Zhang P., Zhang G., Wan X. (2023). Challenges and new technologies in adoptive cell therapy. J. Hematol. Oncol..

[159] Zhou W., Lei S., Liu M., Li D., Huang Y., Hu X., Yang J., Li J., Fu M., Zhang M. (2022). Injectable and photocurable CAR-T cell formulation enhances the anti-tumor activity to melanoma in mice. Biomaterials.

[160] Agliardi G., Dias J., Rampotas A., Garcia J., Roddie C. (2025). Accelerating and optimising CAR T-cell manufacture to deliver better patient products. Lancet Haematol..

[161] Tian Y., Wang K., Xu C., Feng J., Zhang Z.-L. (2022). Non-invasive T cells adoptive immunotherapy for solid tumor with gel anti-tumor T-cell injections. Chem. Eng. J..

[162] Inozume T., Hanada K.-I., Wang Q.J., Ahmadzadeh M., Wunderlich J.R., Rosenberg S.A., Yang J.C. (2010). Selection of CD8+PD-1+ lymphocytes in fresh human melanomas enriches for tumor-reactive T-cells. J. Immunother..

[163] Li T., Zhao L., Yang Y., Wang Y., Zhang Y., Guo J., Chen G., Qin P., Xu B., Ma B. (2021). T Cells Expanded from PD-1+ Peripheral Blood Lymphocytes Share More Clones with Paired Tumor-Infiltrating Lymphocytes. Cancer Res..

[164] Li S., Yao Z.-C., Wang H., Ecker J.A., Omotoso M.O., Lee J., Kong J., Feng H., Chaisawangwong W., Kang S.-S. (2024). Ex vivo expansion and hydrogel-mediated in vivo delivery of tissue-resident memory T cells for immunotherapy. Sci. Adv..

[165] Savas P., Virassamy B., Ye C., Salim A., Mintoff C.P., Caramia F., Salgado R., Byrne D.J., Teo Z.L., Dushyanthen S. (2018). Single-cell profiling of breast cancer T cells reveals a tissue-resident memory subset associated with improved prognosis. Nat. Med..

[166] Duhen T., Duhen R., Montler R., Moses J., Moudgil T., de Miranda N.F., Goodall C.P., Blair T.C., Fox B.A., McDermott J.E. (2018). Co-expression of CD39 and CD103 identifies tumor-reactive CD8 T cells in human solid tumors. Nat. Commun..

[167] Edwards J., Wilmott J.S., Madore J., Gide T.N., Quek C., Tasker A., Ferguson A., Chen J., Hewavisenti R., Hersey P. (2018). CD103^+^ Tumor-Resident CD8^+^ T Cells Are Associated with Improved Survival in Immunotherapy-Naïve Melanoma Patients and Expand Significantly During Anti–PD-1 Treatment. Clin. Cancer Res..

[168] Cui H., Zhao Y.-Y., Han Y.-H., Lan Z., Zou K.-L., Cheng G.-W., Chen H., Zhong P.-L., Chen Y., Ma L.-M. (2024). Lymph node targeting strategy using a hydrogel sustained-release system to load effector memory T cells improves the anti-tumor efficacy of anti-PD-1. Acta Biomater..

[169] Livingston N.K., Hickey J.W., Sim H., Salathe S.F., Choy J., Kong J., Silver A.B., Stelzel J.L., Omotoso M.O., Li S. (2024). In Vivo Stimulation of Therapeutic Antigen-Specific T Cells in an Artificial Lymph Node Matrix. Adv. Mater..

[170] Wei P.-S., Chou P.-Y., Hsu H.-Y., Chen M., Chen Y.-J., Tsai T.-H., Wen B.-Y., Sheu M.-T., Chuang K.-H., Lin H.-L. (2025). Nonshrinkable Thermosensitive Hydrogels Combined with Bispecific Anti-PSMA/CD3 T-Cell Engager for Effective Against Tumors in Mice Model. Int. J. Nanomed..

[171] Shou X., Wu D., Chen C., Shi L., Shang L., Zhao Y., Shen X. (2024). Biomimetic cascade-released hydrogel scaffolds from microfluidics for efficient T cell recruitment and expansion. Chem. Eng. J..

[172] He J., Niu J., Wang L., Zhang W., He X., Zhang X., Hu W., Tang Y., Yang H., Sun J. (2024). An injectable hydrogel microsphere-integrated training court to inspire tumor-infiltrating T lymphocyte potential. Biomaterials.

[173] Wei P.-S., Chen Y.-J., Lin S.-Y., Chuang K.-H., Sheu M.-T., Ho H.-O. (2021). In situ subcutaneously injectable thermosensitive PEG-PLGA diblock and PLGA-PEG-PLGA triblock copolymer composite as sustained delivery of bispecific anti-CD3 scFv T-cell/anti-EGFR Fab Engager (BiTEE). Biomaterials.

[174] Paredes-Moscosso S.R., Nathwani A.C. (2024). 10 years of BiTE immunotherapy: An overview with a focus on pancreatic cancer. Front. Oncol..

[175] Pinto E., Lione L., Compagnone M., Paccagnella M., Salvatori E., Greco M., Frezza V., Marra E., Aurisicchio L., Roscilli G. (2025). From ex vivo to in vivo chimeric antigen T cells manufacturing: New horizons for CAR T-cell based therapy. J. Transl. Med..

[176] Chen T., Wang M., Chen Y., Liu Y. (2024). Current challenges and therapeutic advances of CAR-T cell therapy for solid tumors. Cancer Cell Int..

[177] Zhu C., Ke L., Ao X., Chen Y., Cheng H., Xin H., Xu X., Loh X., Li Z., Lyu H. (2024). Injectable Supramolecular Hydrogels for In Situ Programming of Car-T Cells toward Solid Tumor Immunotherapy. Adv. Mater..

[178] Ozga A.J., Chow M.T., Luster A.D. (2021). Chemokines and the immune response to cancer. Immunity.

[179] Zhu Y., Jin L., Chen J., Su M., Sun T., Yang X. (2023). Promoting the Recruitment, Engagement, and Reinvigoration of Effector T Cells via an Injectable Hydrogel with a Supramolecular Binding Capability for Cancer Immunotherapy. Adv. Mater..

[180] Li J., Ding Z., Liu J., Li G., Li Y., Wang W., Nundlall K., Deng Y., Miao J., Hu M. (2025). Reshaping tumor immune microenvironment and modulating T cell function based on hierarchical nanotherapeutics for synergistically inhibiting osteosarcoma. Mater. Today Bio.

[181] Xu G., Liu K., Chen X., Lin Y., Yu C., Nie X., He W., Karin N., Luan Y. (2024). Hydrogel-mediated tumor T cell infiltration and immune evasion to reinforce cancer immunotherapy. Nanoscale Horiz..

[182] Jia G., Li Q., Tang M., He W., Jiang Y., Tian X., Luan Y. (2025). Genetically Engineered Bacteria-Derived Hydrogel Orchestrates T Cell Spatial Dynamics for Triple-Negative Breast Cancer Immunotherapy. Adv. Funct. Mater..

[183] Chen H., Cong X., Wu C., Wu X., Wang J., Mao K., Li J., Zhu G., Liu F., Meng X. (2020). Intratumoral delivery of CCL25 enhances immunotherapy against triple-negative breast cancer by recruiting CCR9+ T cells. Sci. Adv..

[184] Baessler A., Vignali D.A.A. (2024). T Cell Exhaustion. Annu. Rev. Immunol..

[185] Zhang D., Li Q., Chen X., Nie X., Xue F., Xu W., Luan Y. (2022). An Injectable Hydrogel to Modulate T Cells for Cancer Immunotherapy. Small.

[186] Chen X., Jiang Z., Lin Y., Yu C., Nie X., Xu G., Xu W., Jiang Y., Luan Y. (2023). Tumor lysates-constructed hydrogel to potentiate tumor immunotherapy. J. Control. Release.

[187] Zhang Y., Wang J., Qing G., Wang Y., Li X., Luo T., Wang Y.-F., Liu L., Wang Y., Ni Q. (2025). Controlling T cell-tumor cell interaction with a biomimetic physical barrier for cancer immunotherapy. Proc. Natl. Acad. Sci. USA.

[188] Moon C.Y., Belabed M., Park M.D., Mattiuz R., Puleston D., Merad M. (2025). Dendritic cell maturation in cancer. Nat. Rev. Cancer.

[189] Wculek S.K., Cueto F.J., Mujal A.M., Melero I., Krummel M.F., Sancho D. (2020). Dendritic cells in cancer immunology and immunotherapy. Nat. Rev. Immunol..

[190] Fu C., Ma T., Zhou L., Mi Q.-S., Jiang A. (2022). Dendritic Cell-Based Vaccines Against Cancer: Challenges, Advances and Future Opportunities. Immunol. Investig..

[191] Cheever M.A., Higano C.S. (2011). PROVENGE (Sipuleucel-T) in Prostate Cancer: The First FDA-Approved Therapeutic Cancer Vaccine. Clin. Cancer Res..

[192] Yang P., Song H., Qin Y., Huang P., Zhang C., Kong D., Wang W. (2018). Engineering Dendritic-Cell-Based Vaccines and PD-1 Blockade in Self-Assembled Peptide Nanofibrous Hydrogel to Amplify Antitumor T-Cell Immunity. Nano Lett..

[193] Yang A., Bai Y., Dong X., Ma T., Zhu D., Mei L., Lv F. (2021). Hydrogel/nanoadjuvant-mediated combined cell vaccines for cancer immunotherapy. Acta Biomater..

[194] Ashour D., Arampatzi P., Pavlovic V., Förstner K.U., Kaisho T., Beilhack A., Erhard F., Lutz M.B. (2020). IL-12 from endogenous cDC1, and not vaccine DC, is required for Th1 induction. J. Clin. Investig..

[195] Dekaban G.A., Hamilton A.M., Fink C.A., Au B., de Chickera S.N., Ribot E.J., Foster P.J. (2013). Tracking and evaluation of dendritic cell migration by cellular magnetic resonance imaging. WIREs Nanomed. Nanobiotechnol..

[196] Merad M., Sathe P., Helft J., Miller J., Mortha A. (2013). The Dendritic Cell Lineage: Ontogeny and Function of Dendritic Cells and Their Subsets in the Steady State and the Inflamed Setting. Annu. Rev. Immunol..

[197] Wan C., Sun Y., Hu Y., Huang J., Lu L., Gao Y., Zi H., He Q., Sun J., Lovell J.F. (2021). Peptide hydrogels loaded with irradiated tumor cell secretions enhance cancer immunotherapy. Nano Today.

[198] Gu Z., Chen G., Gao N., Yao S., Zhang X., Xu Q., Xiong W., Liu L., Liu Q., Yin D. (2025). In-situ trichosanthin-IL2/pectin dynamic hydrogel activates dendritic cells and reverses T cell exhaustion for post-operative cancer therapy. Chem. Eng. J..

[199] Huo W., Yang X., Wang B., Cao L., Fang Z., Li Z., Liu H., Liang X.-J., Zhang J., Jin Y. (2022). Biomineralized hydrogel DC vaccine for cancer immunotherapy: A boosting strategy via improving immunogenicity and reversing immune-inhibitory microenvironment. Biomaterials.

[200] Bandola-Simon J., Roche P.A. (2019). Dysfunction of antigen processing and presentation by dendritic cells in cancer. Mol. Immunol..

[201] Song H., Huang P., Niu J., Shi G., Zhang C., Kong D., Wang W. (2018). Injectable polypeptide hydrogel for dual-delivery of antigen and TLR3 agonist to modulate dendritic cells in vivo and enhance potent cytotoxic T-lymphocyte response against melanoma. Biomaterials.

[202] Han J., Bhatta R., Liu Y., Bo Y., Wang H. (2022). In Situ Dendritic Cell Recruitment and T Cell Activation for Cancer Immunotherapy. Front. Pharmacol..

[203] Bhatta R., Han J., Liu Y., Bo Y., Wang Y., Nguyen D., Chen Q., Wang H. (2025). Injectable extracellular vesicle hydrogels with tunable viscoelasticity for depot vaccine. Nat. Commun..

[204] Singh A., Suri S., Roy K. (2009). In-situ crosslinking hydrogels for combinatorial delivery of chemokines and siRNA–DNA carrying microparticles to dendritic cells. Biomaterials.

[205] Ke Y., Zhu J., Chu Y., Cen L., Fu Y., Fan X., Shao J., Li R., Yu L., Liu B. (2022). Bifunctional Fusion Membrane-Based Hydrogel Enhances Antitumor Potency of Autologous Cancer Vaccines by Activating Dendritic Cells. Adv. Funct. Mater..

[206] Ji P., Sun W., Zhang S., Xing Y., Wang C., Wei M., Li Q., Ji G., Yang G. (2023). Modular Hydrogel Vaccine for Programmable and Coordinate Elicitation of Cancer Immunotherapy. Adv. Sci..

[207] Pradhan P., Qin H., Leleux J.A., Gwak D., Sakamaki I., Kwak L.W., Roy K. (2014). The effect of combined IL10 siRNA and CpG ODN as pathogen-mimicking microparticles on Th1/Th2 cytokine balance in dendritic cells and protective immunity against B cell lymphoma. Biomaterials.

[208] Gao T., Yuan S., Liang S., Huang X., Liu J., Gu P., Fu S., Zhang N., Liu Y. (2024). In Situ Hydrogel Modulates cDC1-Based Antigen Presentation and Cancer Stemness to Enhance Cancer Vaccine Efficiency. Adv. Sci..

[209] Do H.T.T., Lee C.H., Cho J. (2020). Chemokines and their Receptors: Multifaceted Roles in Cancer Progression and Potential Value as Cancer Prognostic Markers. Cancers.

[210] Cheng X.-S., Li Y.-F., Tan J., Sun B., Xiao Y.-C., Fang X.-B., Zhang X.-F., Li Q., Dong J.-H., Li M. (2014). CCL20 and CXCL8 synergize to promote progression and poor survival outcome in patients with colorectal cancer by collaborative induction of the epithelial–mesenchymal transition. Cancer Lett..

[211] Jiao X., Shu G., Liu H., Zhang Q., Ma Z., Ren C., Guo H., Shi J., Liu J., Zhang C. (2019). The Diagnostic Value of Chemokine/Chemokine Receptor Pairs in Hepatocellular Carcinoma and Colorectal Liver Metastasis. J. Histochem. Cytochem..

[212] Zhou J., Liu Y., Xu W., Bhatta R., Han J., Baskaran D., Devmal S., Leal C., Wang H. (2025). Macroporous hydrogel-based mRNA cancer vaccine for in situ recruitment and modulation of dendritic cells. Acta Biomater..

[213] MC Lee K., Achuthan A.A., Hamilton J.A. (2020). GM-CSF: A Promising Target in Inflammation and Autoimmunity. ImmunoTargets Ther..

[214] Yang F., Shi K., Jia Y., Hao Y., Peng J., Yuan L., Chen Y., Pan M., Qian Z. (2020). A biodegradable thermosensitive hydrogel vaccine for cancer immunotherapy. Appl. Mater. Today.

[215] Di J., Du Z., Wu K., Jin S., Wang X., Li T., Xu Y. (2022). Biodistribution and Non-linear Gene Expression of mRNA LNPs Affected by Delivery Route and Particle Size. Pharm. Res..

[216] Clemente B., Denis M., Silveira C.P., Schiavetti F., Brazzoli M., Stranges D. (2023). Straight to the point: Targeted mRNA-delivery to immune cells for improved vaccine design. Front. Immunol..

[217] Ali O.A., Verbeke C., Johnson C., Sands R.W., Lewin S.A., White D., Doherty E., Dranoff G., Mooney D.J. (2014). Identification of Immune Factors Regulating Antitumor Immunity Using Polymeric Vaccines with Multiple Adjuvants. Cancer Res..

[218] Embgenbroich M., Burgdorf S. (2018). Current Concepts of Antigen Cross-Presentation. Front. Immunol..

[219] Villadangos J.A., Schnorrer P. (2007). Intrinsic and cooperative antigen-presenting functions of dendritic-cell subsets in vivo. Nat. Rev. Immunol..

[220] Yang K., Zhou Y., Huang B., Zhao G., Geng Y., Wan C., Jiang F., Jin H., Ye C., Chen J. (2023). Sustained release of tumor cell lysate and CpG from an injectable, cytotoxic hydrogel for melanoma immunotherapy. Nanoscale Adv..

[221] de Brito C., Tomkowiak M., Ghittoni R., Caux C., Leverrier Y., Marvel J. (2011). CpG promotes cross-presentation of dead cell-associated antigens by pre-CD8α+ dendritic cells. J. Immunol..

[222] Dongye Z., Li J., Wu Y. (2022). Toll-like receptor 9 agonists and combination therapies: Strategies to modulate the tumour immune microenvironment for systemic anti-tumour immunity. Br. J. Cancer.

[223] Liang X., Li L., Li X., He T., Gong S., Zhu S., Zhang M., Wu Q., Gong C. (2021). A spontaneous multifunctional hydrogel vaccine amplifies the innate immune response to launch a powerful antitumor adaptive immune response. Theranostics.

[224] Li L., Kim S., Herndon J.M., Goedegebuure P., Belt B.A., Satpathy A.T., Fleming T.P., Hansen T.H., Murphy K.M., Gillanders W.E. (2012). Cross-dressed CD8α+/CD103+ dendritic cells prime CD8+ T cells following vaccination. Proc. Natl. Acad. Sci. USA.

[225] Li B., Lu C., Oveissi S., Song J., Xiao K., Zanker D., Duan M.B., Chen J., Xu H., Zou Q. (2020). Host CD8α+ and CD103+ dendritic cells prime transplant antigen-specific CD8+ T cells via cross-dressing. Immunol. Cell Biol..

[226] Abbaszadeh M., Naseri B., Taghizadeh-Teymorloei M., Mardi A., Javan M.R., Masoumi J., Ghorbaninezhad F., Hatami-Sadr A., Tural Ş., Baradaran B. (2025). Overview of dendritic cells subsets and their involvement in immune-related pathological disease. BioImpacts BI.

[227] Morante-Palacios O., Fondelli F., Ballestar E., Martínez-Cáceres E.M. (2021). Tolerogenic Dendritic Cells in Autoimmunity and Inflammatory Diseases. Trends Immunol..

[228] Ochando J., Ordikhani F., Jordan S., Boros P., Thomson A.W. (2020). Tolerogenic dendritic cells in organ transplantation. Transpl. Int..

[229] Fu W., Li X., Li Y., Luo R., Ou C., Huang D., Liang X., You Y., Wu Q., Gong C. (2024). A programmable releasing versatile hydrogel platform boosts systemic immune responses via sculpting tumor immunogenicity and reversing tolerogenic dendritic cells. Biomaterials.

[230] Brancewicz J., Wójcik N., Sarnowska Z., Robak J., Król M. (2025). The Multifaceted Role of Macrophages in Biology and Diseases. Int. J. Mol. Sci..

[231] Strizova Z., Benesova I., Bartolini R., Novysedlak R., Cecrdlova E., Foley L.K., Striz I. (2023). M1/M2 macrophages and their overlaps—Myth or reality?. Clin. Sci..

[232] Parsa R., Andresen P., Gillett A., Mia S., Zhang X.-M., Mayans S., Holmberg D., Harris R.A. (2012). Adoptive Transfer of Immunomodulatory M2 Macrophages Prevents Type 1 Diabetes in NOD Mice. Diabetes.

[233] Czaja A.J. (2015). Adoptive cell transfer in autoimmune hepatitis. Expert Rev. Gastroenterol. Hepatol..

[234] Park K.S., Gottlieb A.P., Janes M.E., Prakash S., Kapate N., Suja V.C., Wang L.L.-W., Guerriero J.L., Mitragotri S. (2025). Adoptively transferred macrophages for cancer immunotherapy. J. Immunother. Cancer.

[235] Sloas C., Gill S., Klichinsky M. (2021). Engineered CAR-Macrophages as Adoptive Immunotherapies for Solid Tumors. Front. Immunol..

[236] Andreesen R., Hennemann B., Krause S.W. (1998). Adoptive immunotherapy of cancer using monocyte-derived macrophages: Rationale, current status, and perspectives. J. Leukoc. Biol..

[237] de Gramont A., Gangji D., Louvet C., Garcia M.L., Tardy D., Romet-Lemonne J.L. (2002). Adoptive Immunotherapy of Ovarian Carcinoma: To the Editor. Gynecol. Oncol..

[238] Lesimple T., Moisan A., Guillé F., Leberre C., Audran R., Drenou B., Toujas L. (2000). Treatment of Metastatic Renal Cell Carcinoma With Activated Autologous Macrophages and Granulocyte–Macrophage Colony-Stimulating Factor. J. Immunother..

[239] Thiounn N., Pages F., Mejean A., Descotes J.-L., Fridman W.-H., Romet-Lemonne J.-L. (2002). Adoptive Immunotherapy For Superficial Bladder Cancer With Autologous Macrophage Activated Killer Cells. J. Urol..

[240] Guerra A.D., Yeung O.W., Qi X., Kao W.J., Man K. (2017). The Anti-Tumor Effects of M1 Macrophage-Loaded Poly (ethylene glycol) and Gelatin-Based Hydrogels on Hepatocellular Carcinoma. Theranostics.

[241] Jiang L., Ding Y., Xue X., Zhou S., Li C., Zhang X., Jiang X. (2018). Entrapping multifunctional dendritic nanoparticles into a hydrogel for local therapeutic delivery and synergetic immunochemotherapy. Nano Res..

[242] Jiang Y., Wang L., Chen Y., Li Y., Chen G., Lu Y., Jiang C., Chai K., Wang Y. (2025). Polyphyllin I enhances the anti-tumor efficacy of Palbociclib by reversing M2 macrophage polarization in lung cancer. Biochem. Biophys. Res. Commun..

[243] Yuan S., Liu B.-H., Cheng W.-W., Meng H., Hou X.-T., Xue J.-C., Zhang H.-M., Zhang Q.-G. (2025). Polyphyllin VI modulates macrophage polarization through autophagy-NLRP3 inflammasome to alleviate inflammatory bowel disease. Phytomedicine.

[244] Yang Y., Yang Y., Chen M., Chen J., Wang J., Ma Y., Qian H. (2021). Injectable shear-thinning polylysine hydrogels for localized immunotherapy of gastric cancer through repolar-ization of tumor-associated macrophages. Biomater. Sci..

[245] Dai X., Meng J., Deng S., Zhang L., Wan C., Lu L., Huang J., Hu Y., Zhang Z., Li Y. (2020). Targeting CAMKII to reprogram tumor-associated macrophages and inhibit tumor cells for cancer immunotherapy with an injectable hybrid peptide hydrogel. Theranostics.

[246] Lee C., Bae S.-J.S., Joo H., Bae H. (2017). Melittin suppresses tumor progression by regulating tumor-associated macrophages in a Lewis lung carcinoma mouse model. Oncotarget.

[247] Pandey P., Khan F., Khan M.A., Kumar R., Upadhyay T.K. (2023). An Updated Review Summarizing the Anticancer Efficacy of Melittin from Bee Venom in Several Models of Human Cancers. Nutrients.

[248] Li Q., Song Q., Zhao Z., Lin Y., Cheng Y., Karin N., Luan Y. (2023). Genetically Engineered Artificial Exosome-Constructed Hydrogel for Ovarian Cancer Therapy. ACS Nano.

[249] Jablonski K.A., Amici S.A., Webb L.M., Ruiz-Rosado J.D.D., Popovich P.G., Partida-Sanchez S., Guerau-De-Arellano M. (2015). Novel Markers to Delineate Murine M1 and M2 Macrophages. PLoS ONE.

[250] Kovaleva O.V., Rashidova M.A., Sinyov V.V., Malashenko O.S., Gratchev A. (2025). M1 macrophages—Unexpected contribution to tumor progression. Front. Immunol..

[251] Wang Q., Ma W. (2025). Revisiting TAM polarization: Beyond M1- and M2-type TAM toward clinical precision in macrophage-targeted therapy. Exp. Mol. Pathol..

[252] de Visser K.E., Joyce J.A. (2023). The evolving tumor microenvironment: From cancer initiation to metastatic outgrowth. Cancer Cell.

[253] Waldman A.D., Fritz J.M., Lenardo M.J. (2020). A guide to cancer immunotherapy: From T cell basic science to clinical practice. Nat. Rev. Immunol..

[254] Heremans J., Awad R.M., Bridoux J., Ertveldt T., Caveliers V., Madder A., Hoogenboom R., Devoogdt N., Ballet S., Hernot S. (2024). Sustained release of a human PD-L1 single-domain antibody using peptide-based hydrogels. Eur. J. Pharm. Biopharm..

[255] Harui A., Roth M.D. (2022). Hyaluronidase Enhances Targeting of Hydrogel-Encapsulated Anti-CTLA-4 to Tumor Draining Lymph Nodes and Improves Anti-Tumor Efficacy. Gels.

[256] Kim J., Francis D.M., Thomas S.N. (2021). In Situ Crosslinked Hydrogel Depot for Sustained Antibody Release Improves Immune Checkpoint Blockade Cancer Immunotherapy. Nanomaterials.

[257] Kim J., Francis D.M., Sestito L.F., Archer P.A., Manspeaker M.P., O’melia M.J., Thomas S.N. (2022). Thermosensitive hydrogel releasing nitric oxide donor and anti-CTLA-4 micelles for anti-tumor immunotherapy. Nat. Commun..

[258] Li Y., Fang M., Zhang J., Wang J., Song Y., Shi J., Li W., Wu G., Ren J., Wang Z. (2016). Hydrogel dual delivered celecoxib and anti-PD-1 synergistically improve antitumor immunity. OncoImmunology.

[259] Liu M., Cao Z., Zhang R., Chen Y., Yang X. (2021). Injectable Supramolecular Hydrogel for Locoregional Immune Checkpoint Blockade and Enhanced Cancer Chemo-Immunotherapy. ACS Appl. Mater. Interfaces.

[260] Zhai L., Shi Y., Yan Y., Lu A., Liu X., Lei L., Sun Y., Jiang L., Wang X., Qian H. (2023). Local sustained release of PD-1 monoclonal antibody and lenvatinib by thermo-sensitive hydrogel for improving tumor immunotherapy. Chin. Chem. Lett..

[261] Wang F., Xu D., Su H., Zhang W., Sun X., Monroe M.K., Chakroun R.W., Wang Z., Dai W., Oh R. (2020). Supramolecular prodrug hydrogelator as an immune booster for checkpoint blocker–based immunotherapy. Sci. Adv..

[262] Gerstberger S., Jiang Q., Ganesh K. (2023). Metastasis. Cell.

[263] Rezaeeyan H., Shirzad R., McKee T.D., Saki N. (2018). Role of chemokines in metastatic niche: New insights along with a diagnostic and prognostic approach. APMIS.

[264] Ieranò C., D’alterio C., Giarra S., Napolitano M., Rea G., Portella L., Santagata A., Trotta A.M., Barbieri A., Campani V. (2019). CXCL12 loaded-dermal filler captures CXCR4 expressing melanoma circulating tumor cells. Cell Death Dis..

[265] Chen C., Liu J., Zhang H., Zhang H., Liang Y., Ye Q., Shen W., Luo H., Guo L. (2024). A Bait-and-Hook Hydrogel for Net Tumor Cells to Enhance Chemotherapy and Mitigate Metastatic Dissemination. Pharmaceutics.

[266] The National Academies of Sciences, Engineering, and Medicine (2022). Tissue Homeostasis, Inflammation, and Repair. Understanding the Role of the Immune System in Improving Tissue Regeneration: Proceedings of a Workshop.

[267] Valls P.O., Esposito A. (2022). Signalling dynamics, cell decisions, and homeostatic control in health and disease. Curr. Opin. Cell Biol..

[268] Wang Y., Zhou H., Ju S., Dong X., Zheng C. (2025). The solid tumor microenvironment and related targeting strategies: A concise review. Front. Immunol..

[269] Sinha A., Ghosh D., Karati D. (2025). Tumor microenvironment and immunotherapy: From bench to bedside. Med. Oncol..

[270] Hosonuma M., Yoshimura K. (2023). Association between pH regulation of the tumor microenvironment and immunological state. Front. Oncol..

[271] Wu H., Yin Y., Hu X., Peng C., Liu Y., Li Q., Huang W., Huang Q. (2019). Effects of Environmental pH on Macrophage Polarization and Osteoimmunomodulation. ACS Biomater. Sci. Eng..

[272] Liang J.-L., Jin X.-K., Luo G.-F., Zhang S.-M., Huang Q.-X., Lin Y.-T., Deng X.-C., Wang J.-W., Chen W.-H., Zhang X.-Z. (2023). Immunostimulant Hydrogel-Guided Tumor Microenvironment Reprogramming to Efficiently Potentiate Macrophage-Mediated Cellular Phagocytosis for Systemic Cancer Immunotherapy. ACS Nano.

[273] Noman M.Z., Hasmim M., Messai Y., Terry S., Kieda C., Janji B., Chouaib S. (2015). Hypoxia: A key player in antitumor immune response. A Review in the Theme: Cellular Responses to Hypoxia. Am. J. Physiol. Cell Physiol..

[274] Zhang W., Shi Y., Li H., Yu M., Zhao J., Chen H., Kong M. (2022). In situ injectable nano-complexed hydrogel based on chitosan/dextran for combining tumor therapy via hypoxia alleviation and TAMs polarity regulation. Carbohydr. Polym..

[275] Zhang M., Liu X., Mao Y., He Y., Xu J., Zheng F., Tan W., Rong S., Chen Y., Jia X. (2022). Oxygen-Generating Hydrogels Overcome Tumor Hypoxia to Enhance Photodynamic/Gas Synergistic Therapy. ACS Appl. Mater. Interfaces.

[276] Wan Y., Fu L., Li C., Lin J., Huang P. (2021). Conquering the Hypoxia Limitation for Photodynamic Therapy. Adv. Mater..

[277] Wheaton W.W., Weinberg S.E., Hamanaka R.B., Soberanes S., Sullivan L.B., Anso E., Glasauer A., Dufour E., Mutlu G.M., Budigner G.S. (2014). Metformin inhibits mitochondrial complex I of cancer cells to reduce tumorigenesis. eLife.

[278] Wang D., DuBois R.N. (2015). Immunosuppression associated with chronic inflammation in the tumor microenvironment. Carcinogenesis.

[279] Chen M., Tan Y., Dong Z., Lu J., Han X., Jin Q., Zhu W., Shen J., Cheng L., Liu Z. (2020). Injectable Anti-inflammatory Nanofiber Hydrogel to Achieve Systemic Immunotherapy Post Local Administration. Nano Lett..

[280] Gao C., Cheng K., Li Y., Gong R., Zhao X., Nie G., Ren H. (2022). Injectable Immunotherapeutic Hydrogel Containing RNA-Loaded Lipid Nanoparticles Reshapes Tumor Microenvironment for Pancreatic Cancer Therapy. Nano Lett..

[281] Wang B., Chen J., Caserto J.S., Wang X., Ma M. (2022). An in situ hydrogel-mediated chemo-immunometabolic cancer therapy. Nat. Commun..

[282] Liu Z., Liu X., Shen H., Xu X., Zhao X., Fu R. (2022). Adenosinergic axis and immune checkpoint combination therapy in tumor: A new perspective for immunotherapy strategy. Front. Immunol..

[283] Zhao Z., Li Q., Qin X., Zhang M., Du Q., Luan Y. (2022). An Injectable Hydrogel Reshaping Adenosinergic Axis for Cancer Therapy. Adv. Funct. Mater..

[284] Li Q., Zhao Z., Qin X., Zhang M., Du Q., Li Z., Luan Y. (2021). A Checkpoint-Regulatable Immune Niche Created by Injectable Hydrogel for Tumor Therapy. Adv. Funct. Mater..

[285] Schumacher T.N., Thommen D.S. (2022). Tertiary lymphoid structures in cancer. Science.

[286] Sautès-Fridman C., Petitprez F., Calderaro J., Fridman W.H. (2019). Tertiary lymphoid structures in the era of cancer immuno-therapy. Nat. Rev. Cancer.

[287] Kuwentrai C., Tang W., Lin X., Chi T., Liu D., Song E., Webber M.J., Huang J.-D., Ye Z. (2025). Injectable hydrogel-based drug formulation for enhancing tertiary lymphoid structure formation and cancer immunotherapy efficacy. J. Control. Release.

[288] Ding J., Wang T., Lin Z., Li Z., Yang J., Li F., Rong Y., Chen X., He C. (2025). Chiral polypeptide hydrogels regulating local immune microenvironment and anti-tumor immune response. Nat. Commun..

[289] Muhammad S.N., Zakariya Z.T., Shaji S., Sunilkumar A.K., George A., Radhakrishnan S.P., Nair S.V., Koyakutty M. (2025). Injectable Immune-Engineered Hydrogel Niche Remote from the Immune Suppressed Tumor Microenvironment for Cancer Immunotherapy. Adv. Ther..

[290] Rafael D., Melendres M.M.R., Andrade F., Montero S., Martinez-Trucharte F., Vilar-Hernandez M., Durán-Lara E.F., Schwartz S., Abasolo I. (2021). Thermo-responsive hydrogels for cancer local therapy: Challenges and state-of-art. Int. J. Pharm..

[291] Clegg J.R., Adebowale K., Zhao Z., Mitragotri S. (2024). Hydrogels in the clinic: An update. Bioeng. Transl. Med..

[292] Mariados N., Sylvester J., Shah D., Karsh L., Hudes R., Beyer D., Kurtzman S., Bogart J., Hsi R.A., Kos M. (2015). Hydrogel Spacer Prospective Multicenter Randomized Controlled Pivotal Trial: Dosimetric and Clinical Effects of Perirectal Spacer Application in Men Undergoing Prostate Image Guided Intensity Modulated Radiation Therapy. Int. J. Radiat. Oncol..

[293] Armstrong N., Bahl A., Pinkawa M., Ryder S., Ahmadu C., Ross J., Bhattacharyya S., Woodward E., Battaglia S., Binns J. (2021). SpaceOAR Hydrogel Spacer for Reducing Radiation Toxicity During Radiotherapy for Prostate Cancer. A Systematic Review. Urology.

[294] Sidney Kimmel Comprehensive Cancer Center at Johns Hopkins (2024). Evaluation of a Novel Absorbable Radiopaque Hydrogel in Patients Undergoing Image-Guided Radiotherapy for Pancreatic Adenocarcinoma. https://clinicaltrials.gov/study/NCT03307564.

[295] de Baere T., Plotkin S., Yu R., Sutter A., Wu Y., Cruise G.M. (2016). An In Vitro Evaluation of Four Types of Drug-Eluting Microspheres Loaded with Doxorubicin. J. Vasc. Interv. Radiol..

